# Individual sedentary activities and cognitive function in middle-aged and older adults: A systematic review

**DOI:** 10.1177/13872877251394751

**Published:** 2025-12-22

**Authors:** Jiatong Chen, Kirsten Dillon-Rossiter, Lily Grigsby-Duffy, Anisa Morava, Adam Novic, Babac Salmani, Siobhan Smith, Harry Prapavessis, Paul A Gardiner

**Affiliations:** 1 The University of Queensland, School of Public Health, Brisbane, Australia; 2 Western University, London, Ontario, Canada; 3 Lung Foundation Australia, Brisbane, Australia; 4 Griffith University, Brisbane, Australia

**Keywords:** aging, Alzheimer's disease, cognition, cognitive dysfunction, sedentary activity, sedentary behavior

## Abstract

**Background:**

The influence of lifestyle factors on cognitive health, particularly among middle-aged and older adults, has garnered significant attention in gerontology and cognitive neuroscience. Currently, over 55 million people worldwide are living with dementia, including Alzheimer's disease with nearly 10 million new cases annually. Although sedentary behavior has been associated with cognitive decline, these studies often treat sitting time as a homogeneous entity, without considering the specific nature of sedentary activities.

**Objective:**

This systematic review aims to examine associations of individual sitting activities with cognitive function in middle-aged and older adults.

**Methods:**

Data were searched using eight electronic databases (EMBASE, Web of Science, PsycINFO, CINAHL, Medline, SPORTDiscus, PubMed, and Scopus) from inception to September 2024. Qualitative studies, reviews, conference abstracts, theses, and book chapters were excluded. The methodological quality of included studies was assessed using the QualSyst tool.

**Results:**

A total of 85 studies (n = 1,575,657) were included in this review. Of the 43 studies examining television viewing, 28 (65%) reported a negative association between prolonged television viewing and cognitive function. Conversely, among the 58 studies that included active sedentary activities, only five (8.6%) reported negative associations.

**Conclusions:**

The cognitive effects of sedentary behavior depend on the type of activity performed. Promoting cognitively engaging sedentary activities may support healthy cognitive aging, while excessive passive behaviors may increase risk of cognitive decline and Alzheimer's disease. Future research should focus on further clarifying the mechanisms behind these associations and explore interventions to enhance cognitive health in aging populations.

## Introduction

The global population is aging, such that by 2050, there will be 426 million people aged 80 years or older.^1^ With this global aging population, cognitive health, particularly among middle-aged and older adults, has attracted growing interest in gerontology, cognitive neuroscience, and public health. Cognitive functioning encompasses the mental processes involved in acquiring knowledge and understanding, including several domains such as attention, language, executive functioning, learning, memory, intelligence quotient, and visuospatial abilities, essential for performing daily tasks and maintaining independence.^2^ Cognitive abilities often exhibit a decline with aging. Declines in cognition beyond that due to aging may result in dementia. Dementia is an umbrella term that encompasses several conditions, with Alzheimer's disease (AD) being the most common. Dementia is characterized by a loss of cognitive functions such as thinking, remembering, and reasoning,^3^ such that it impairs daily life. Although dementia is not a normal part of aging, the risk of developing it significantly increases with age.^3^

Currently, more than 55 million people worldwide are living with dementia, with nearly 10 million new cases each year.^4^ Dementia is the seventh leading cause of death globally and one of the leading causes of disability and dependency among older people worldwide.^5^ The impact of dementia extends beyond individuals, affecting families and healthcare systems. In 2019, the global economic cost of dementia was estimated at US $1313.4 billion, involving 55.2 million individuals, equating to US $23,796 per person with dementia.^6^ There is currently no cure for dementia,^6^ making it critical to identify modifiable risk factors that can help prevent or delay cognitive decline and the onset of dementia. Several risk factors have been associated with the development of dementia. The 2024 Lancet Commission on dementia prevention, intervention, and care report has highlighted that approximately 45% of dementia cases can be prevented by targeting 14 modifiable risk factors throughout different life stages.^7^ The early life stage factor is lower level of education (5%). The midlife stage factors are hearing loss (7%), elevated HDL cholesterol (7%), depression (3%), traumatic brain injury (3%), lack of physical activity (2%), diabetes (2%), smoking (2%), hypertension (2%), obesity (1%), and excessive alcohol consumption (1%). The late life stage factors are social isolation (5%), exposure to air pollution (3%), and untreated vision problems (2%).^7^ Fifty-five percent of cases remain unclear, suggesting the need to investigate other risk factors.

In recent years, increasing time spent sedentary in mid-aged and older adults, has emerged as a crucial predictor of healthy aging.^8–10^ Sedentary time is defined as waking activities, while sitting or lying, with an energy expenditure of 1.5 metabolic equivalent tasks (MET) or less, such as watching television, working at a desk, and using a computer.^11^ Researchers can investigate the impact of both total sedentary time and specific types of sedentary activities to better understand their associations with various health outcomes. Sedentary time is highly prevalent among older adults and tends to increase with age, often exacerbated by comorbidities and cognitive decline.^12^ A meta-analysis involving 349,698 adults aged 60 years and older, encompassing 22 studies (10 national and one EU-wide), reported that older adults spend an average of 9.4 h per day in sedentary activities, accounting for 65–80% of their waking hours.^13^

Numerous studies have established a link between sedentary activities and heightened rates of all-cause mortality, cardiovascular mortality, cancer mortality, and type 2 diabetes mellitus in older populations.^14,15^ However, the association between sedentary activities and cognitive function remains unclear. Recent studies have shown that prolonged sedentary activities, particularly passive ones like watching television, are linked to faster cognitive decline and poorer performance in global cognition, immediate memory, and verbal fluency,^12,16,17^ while positive impacts on processing speed and executive function have also been observed in reading and playing card games.^18,19^ Therefore, it is critical to consider the quality and context of these activities, as the impact of sedentary activities on cognitive health may vary depending on the nature of the activity.

Previous systematic reviews have often aggregated all forms of sedentary activities, potentially obscuring the distinct effects of specific activities health outcomes. Dillon et al. (2020)'s meta-analysis of 23 studies found that overall, total sedentary time had no significant association with cognitive function, while subgroup analyses showed that device-measured sedentary time had a significant negative association with cognitive function and self-reported sedentary time showed a positive association.^20^ Olanrewaju et al. (2020) reviewed 18 studies and found mixed results in that some studies reported associations between higher sitting time and poorer cognitive outcomes, while others reported no significant association.^10^ Copeland et al. (2017) suggested that cognitively engaging sedentary activities may benefit cognition function, while more passive activities could be detrimental.^21^ Sedentary activities vary significantly in terms of cognitive engagement and an emerging body of research that have examined the impact of so called active and passive sedentary activities on depression.^22^ Research suggests that activities requiring active thinking, problem-solving, and continuous learning can build cognitive reserve, thereby mitigating cognitive decline. For instance, a longitudinal study of a 1946 British birth cohort found that individuals with higher cognitive reserve, measured by educational attainment, occupational complexity, and social and leisure activity participation, performed better on cognitive tests at age 69.^23^

Conversely, passive activities, particularly prolonged television viewing, have been linked to cognitive decline. A cross-sectional study by Guillot et al. (2012) demonstrated that regular computer use potentially helps to preserve cognitive function in older adults compared to television viewing.^24^ The Wingood study on cognitively active versus passive sedentary activities in older adults found that cognitively demanding activities were associated with better performance in memory and executive function than passive activities.^25^ Similarly, a longitudinal survey of healthy longevity in China found that playing card games or engaging in group activities provided social stimulation, reducing the risk of social isolation and its associated cognitive risks.^19^ Additionally, earlier reviews have highlighted significant heterogeneity in study designs, including varying measures of sedentary behavior and cognitive outcomes. This variability can lead to inconsistent findings, complicating the ability to draw definitive conclusions about the relationship between sedentary activities and cognitive function. For example, a current systematic review and meta-analysis reported a weak association between total sedentary time and cognitive function and varied depending on the sedentary activities measure and the cognitive domain assessed.^20^ Specifically, device-based measures of sedentary time showed a negative association with cognitive function, particularly with global cognitive function and processing speed, while self-reported measures showed a positive association with cognitive function, notably with processing speed.^20^ Furthermore, previous reviews have generally not distinguished between the cognitive domains affected by sedentary behavior, as different sedentary activities may influence cognitive functions such as memory, executive function, or attention in diverse ways.

This study aimed to conduct a systematic review to describe the associations between individual sedentary activities and cognitive function in middle-aged and older adults. A key aim was to examine the relationships between various sedentary behaviors and different cognitive domains, such as executive function and memory. Specifically, we aim to determine whether differences exist in the relationship between active sedentary behaviors (e.g., reading or using a computer) and passive sedentary behaviors (e.g., watching television) in relation to cognitive performance.

## Methods

The protocol for this systematic review is pre-registered with Prospero International Prospective Register of Systematic Reviews (registration number: CRD42018082384) - https://www.crd.york.ac.uk/prospero/display_record.php?ID = CRD42018082384). The present systematic review was conducted in accordance with the Preferred Reporting Items for Systematic Reviews and Meta-Analyses (PRISMA) statement guidelines. The PRISMA Checklist is displayed in Supplemental Table 1.

### Search strategy

We comprehensively searched the following electronic databases from inception to September 2024: EMBASE, Web of Science, PsycINFO, CINAHL, Medline, SPORTDiscus, PubMed, and Scopus. The final search strategy and selection of search terms for each database were collaboratively determined by all contributing authors and encompassed three key dimensions: participants (i.e., middle-aged, and older adults), exposure (i.e., individual sedentary activities), and outcome of interest (i.e., cognitive function or cognitive status, e.g., dementia). Searches were conducted using search terms and free-text terms. Each database search was tailored to account for specific database requirements (see Supplemental Table 2 for detailed search strategies). We also included references identified through manual searches in the study team's libraries. The initial search was conducted in February 2024, with additional search and review of sources conducted in September 2024.

### Types of participants

This review specifically focused on middle-aged and older adults, thus only studies with a population mean age of 40 years or older were included, with age referring to the timepoint at which participants were assessed for the analysis. Studies from all countries were considered. Studies including both healthy individuals and those with pre-existing conditions, such as mild cognitive impairment, were eligible for inclusion.

### Types of exposure

The exposure of interest in this review is defined as time spent in individual sedentary activities, including watching television, reading, games and puzzles, driving, and using computer. Although one element of sedentary activity is defined by posture, i.e., sitting or lying, and with a low energy expenditure,^11^ we included activities commonly deemed as sedentary even if posture or energy expenditure was not explicitly assessed. Sedentary activities are also classified into mental active sedentary activities (e.g., computer use, reading, driving, and playing games) and mentally passive sedentary activities (e.g., watching television). In this study, television viewing is the only sedentary behavior categorized as mentally passive, as it is typically associated with low cognitive engagement.^22^ Other activities, such as reading, computer use, or playing games, are considered mentally active due to their higher cognitive demands.^22^ In this review, different card games (poker games and mahjong) and puzzles are grouped together because while the specific content may vary, the cognitive engagement required for these activities is fundamentally similar, all involving problem-solving, memory, and strategic thinking, regardless of the exact nature of the game or puzzle. This is comparable to how people watch different types of content on TV—whether it's news, a movie, or a documentary—they are still engaging in the same passive activity of watching television. Measurement of these activities typically relies on self-report questionnaires or diaries, wherein participants document the duration and frequency of each activity. Studies were included in this review if they specifically reported the time spent on individual sedentary activities, such as the number of hours spent watching television, reading, games and puzzles, driving, or using a computer, either on a daily or weekly basis. Similarly, studies that reported the frequency of these activities (e.g., how often participants engaged in each sedentary activity) were also included. Therefore, any reference to ‘sedentary activities’ in this review refers to distinct and specific sedentary activities, unless otherwise specified.

Importantly, this review excluded studies that focused on cognitive training programs as part of sedentary behavior because such interventions have been reviewed in prior meta-analyses.^26–28^ The exclusion is based on the distinction between every day, non-structured sedentary activities, and structured, goal-oriented cognitive training, which aims to improve cognitive function directly. As our objective is to explore the effects of passive and mentally active sedentary activities on cognitive function in natural settings, studies that involved cognitive training programs were not suitable for this review. Studies that include composite measures of sedentary activities, combining multiple activities into a single exposure, were excluded because they do not allow for the isolation of the effects of individual sedentary activities on cognitive function, which is essential for the objectives of this review. Additionally, studies that reported only total sitting time or lack of physical activity, without distinguishing between specific sedentary activities, were excluded.

### Types of outcome measure

Only studies that assessed cognitive function and/or cognitive impairment/decline (e.g., dementia or mild cognitive impairment [MCI]) were included. The cognitive outcomes were required to be assessed using recognized methods or standardized cognitive tests to ensure consistency and reliability across studies. Each study's cognitive outcomes were categorized according to the authors’ reported assessments of different cognitive domains. These cognitive domains were classified into one or more of the following domains: (1) processing speed, (2) episodic memory, (3) global cognitive function, (4) motor skills and construction, (5) executive function, (6) cognitive flexibility, and (7) working memory. For definitions of each cognitive domain and the acceptable cognitive tests, please refer to Supplemental Table 3. For inclusion, studies had to clearly specify the cognitive domain(s) they assessed, and the assessment tools had to be recognized in the literature as valid and reliable measures for those specific domains. Studies were excluded if they measured cognitive function as defined by brain volume or cerebral blood flow using magnetic resonance imaging (MRI) or if they provided only subjective self-reported assessments of cognitive functioning without any formal testing. In this systematic review, dementia and cognitive impairment were measured and categorized using standard clinical definitions and diagnostic tools. Dementia is classified as a significant decline in cognitive function that interferes with daily living, with AD representing a major subcategory. Cognitive impairment encompasses a broader spectrum of difficulties in thinking, learning, memory, judgment, and decision-making. This category includes subtypes such as MCI, subjective cognitive impairment, and cognitive complaints. For detailed definitions of dementia and cognitive impairment and the measurements, please refer to Supplemental Table 4.

### Study selection

In addition to the aforementioned criteria, additional inclusion criteria for this analysis were studies that were (1) published in any year; (2) available in English language. The exclusion criteria include discussion articles, conference proceedings, book chapters, theses, or commentaries not presenting empirical research in a peer-reviewed journal. Two authors independently screened the titles and abstracts of the identified studies using the Covidence systematic review software, excluding studies that did not meet the inclusion criteria. Both authors then independently assessed the full texts to further identify studies that did not meet the criteria. At each stage of the screening process, any discrepancies between the authors were discussed, and consensus was reached to ensure consistency in selecting eligible studies. All additional articles identified through these alternative sources were evaluated using the same eligibility criteria and screening process as those retrieved from the electronic database searches.

### Data extraction

Data extraction was conducted by one review author, with a second author verifying the accuracy of the extracted data on 10% of included studies. The extracted data are presented in a series of tables, each addressing specific aspects of the studies included in the review. Table 1 provides a summary of the general characteristics of the selected studies, including (1) the author and publication year, (2) the country where the study was conducted, (3) the study design, (4) sample size, (5) mean age of participants, and (6) the percentage of female participants. Tables 2, 3, 4, 5, and 6 focus on the relationship between passive and active sedentary activities and cognitive function separately, including: (1) the types of passive or active sedentary activities examined, (2) how these activities were measured, (3) the cognitive domains assessed, (4) the tools used to measure cognitive function, (5) the covariates included in the analysis, and (6) the main results of each study. Finally, Tables 7 and 8 summarize the findings from studies that investigated associations of sedentary activities with dementia and cognitive impairment. Additional information extracted but not included for reporting in Tables 1–8 included: (1) study objectives, (2) recruitment sources and methods, (3) inclusion and exclusion criteria, (4) data types for exposure and outcome variables, (5) statistical methods, (6) study conclusions, (7) limitations identified by the authors, and (8) disclosure of conflicts of interest.

**Table 1.** Characteristics of included studies.

Author (year), Pinwheel number (PW)	Country	Study design (length of follow-up)	Participants (N)	Mean age	% Female
Allen et al. (2017),^104^ 1	United Kingdom	CS & LO (2 years)	9551	67.9	55.0
Anaraky et al. (2024),^60^ 76	United States	LO (5 years)	2769	74.9	56.2
Anaturk et al. (2021),^31^ 10	United Kingdom	CS	7152	63.9	54.5
Bakrania et al. (2018),^105^ 2	United Kingdom	CS & LO (9 years)	502,643	56.5	54.4
Bernstein et al. (2021),^33^ 67	United States	CS	60	73.4	27.0
Bernstein et al. (2022),^32^ 68	United States	CS	91	73.7	31.5
Brooker et al. (2019),^34^ 75	United Kingdom	CS	19,078	61.1	73.4
Brooker et al. (2019),^107^ 76	United Kingdom	CS	19,078	61.1	73.4
Cansino et al. (2024),^35^ 29	Mexico	CS	1652	51.0	50.3
Cegolon et al. (2022),^61^ 14	Europe	LO (4 years)	45,216		
Chen et al. (2021),^108^ 12	Japan	CS	3708	49.5	50.0
Choi et al. (2021),^62^ 13	United States	LO (4 years)	3904	70–79	59.0
Coelho et al. (2020),^36^ 41	Canada	CS	75	74.6	80.0
Covey et al. (2024),^37^ 61	United States	CS	42	45.3	81.0
Cutting et al. (2023),^38^ 37	United Kingdom	CS	482	49.5	61.6
Da Ronch et al. (2015),^39^ 50	Europe	CS	1381	73.1	47.6
Fajersztajn et al. (2021),^12^ 6	Brazil	LO (2 years)	1243	72.0	61.0
Fancourt et al. (2019),^17^ 7	United Kingdom	LO (6 years)	3590	67.1	56.3
Feng et al. (2020),^63^ 77	China	LO (18 years)	8236	63.0	53.1
Floud et al. (2021),^64^ 49	United Kingdom	LO (8 years)	851,307	60	100
Green et al. (2021),^65^ 22	Europe	LO (11 years)	2015	75.3	49
Hamer et al. (2014),^66^ 56	United Kingdom	LO (2 years)	6359	64.9	54.8
Hartanto et al. (2020),^67^ 23	United States	LO (9 years)	3294	54.54	54.9
Heisz et al. (2015),^41^ 46	Canada	CS	30	74	50
Hoang et al. (2016),^68^ 70	United States	LO (25 years)	3247	50.1	56.5
Hou et al. (2019),^42^ 80	China	CS	33	65.0	45.5
Ihle et al. (2020),^70^ 81	Switzerland	LO (6 years)	897	74.3	51.9
Ivleva et al. (2023),^71^ 30	Europe	LO (9 years)	2320	65–100	65.9
Janoutová et al. (2021),^102^ 82	Czech Republic	Case-controlled	2106	Case: 79.9 Control: 70.7	Case: 75.8 Control: 68.6
Jia et al. (2024),^72^ 83	United Kingdom	LO (4 years)	471,346	56.8	54.5
Jopp et al. (2007),^43^ 47	United States	CS	326	55.5	62.0
Jung et al. (2020),^44^ 39	Korea	CS	168	71.2	
Karsazi et al. (2024),^73^ 57	Iran	LO (3 years)	237	58.8	63.7
Kesse-Guyot et al. (2012),^24^ 53	France	CS & LO (6 years)	2179	65.4	45.0
Kim et al. (2022),^45^ 72	Korea	CS	29	36.9–43.79	24.1
Krell-Roesch et al. (2017),^75^ 24	United States	LO (10 years)	2000	77.8	50.1
Krell-Roesch et al. (2019),^46^ 78	United States	LO (4 years)	1929	77.0	50.4
Kurita et al. (2019),^46^ 28	Japan	CS	5300	75.0	52.9
Kurita et al. (2021),^76^ 66	Japan	LO (5 years)	2010	71.0	52.5
Lindstrom et al. (2005),^98^ 52	United States	Case-controlled	466	40–59	53.3
Lin et al. (2022),^109^ 58	China	LO (12 years)	4440	70–74	46.9
Maasakkers et al. (2020),^110^ 71	Greece	LO (2.7 years)	1551	72.5	60.2
Maasakkers et al. (2021),^77^ 4	Irish	LO (4 years)	1276	67.0	57.0
Mai et al. (2023),^78^ 79	China	LO (20 years)	12,852	84.0	54.6
Major et al. (2023),^16^ 5	United States	LO (2 years)	1261	75.1	52.2
Mao et al. (2020),^79^ 15	China	LO (3.4 years)	10,741	88.0	54.4
Mariano et al. (2021),^80^ 25a	United States	LO (8 years)	3404	64.41	60.2
Mariano et al. (2021),^80^ 25b	German	LO (3.4 years)	4871	63.9	50.8
Mariano et al. (2021),^81^ 64	German	LO (3 years)	3479	61.12	48.6
Mellow et al. (2022),^47^ 42	Australia	CS	384	65.5	68.5
Miller et al. (2024),^48^ 31	United States	CS	1426	77.6	71.2
Nemoto et al. (2022),^82^ 19	Japan	LO (5 years)	5323	74.7	54.5
Nemoto et al. (2018),^49^ 11	Japan	CS	5328	75	54.5
Olanrewaju et al. (2020),^10^ 3	Irish	CS & LO (2 years)	6395	66.4	
Raichlen et al. (2022),^83^ 44	United Kingdom	LO (4 years)	146,651	64.6	49.7
Ramos et al. (2021),^99^ 43	Spain	Case-controlled	497	50.0	73.4
Rawtaer et al. (2020),^50^ 38	Singapore	CS	59	73.0	67.0
Ringin et al. (2023),^51^ 35	United Kingdom	CS	59,653	56.9	47.9
Rosenberg et al. (2016),^52^ 51	United States	CS	307	83.6	72.3
Schaham et al. (2021),^111^ 85	Israel	CS	35	70	60
Sha et al. (2022),^84^ 20	China	LO (16 years)	7422	90.0	65.2
Shi et al. (2023),^53^ 73	China	CS	13,737	62.0	50.4
Shimada et al. (2018),^54^ 62	Japan	CS	4564	71.1	50.2
Shin et al. (2021),^85^ 8	United States	LO (14 years)	3793	73.0	55.9
Shuai et al. (2023),^55^ 9	China	CS & LO (2 years)	5356	70.9	53.7
Takeuchi et al. (2023),^87^ 21	United Kingdom	LO (13 years)	502,505	56.5	54.4
Takeuchi et al. (2022),^86^ 16	United Kingdom	LO (5 years)	502,505	55.9–64.1	39.7–52.8
Tan et al. (2024),^56^ 63	United Kingdom	CS	19,773	68.5	51.0
Tarawit et al. (2021),^57^ 54	Thailand	CS	295	79.0	79.0
Wanders et al. (2021),^88^ 26	Netherlands	LO (7 years)	2237	61.0	43.0
Wang et al. (2006),^89^ 55	China	LO (5 years)	5437	62.8–68.5	47.2–57.5
Wei et al. (2023),^90^ 40	China	LO (16 years)	5246	60.8	49.1
Wingood et al. (2024),^25^ 32	United States	LO (5 years)	2244	70–74	55.0
Woods et al. (2023),^100^ 59	United States	Case-controlled	186	HIV+: 56.5 HIV-: 57.1	HIV+: 14.6% HIV-: 26.3%
Wu et al. (2023),^91^ 27	Australia	LO (3 years)	10,318	73.8	52.6
Wu et al. (2019),^58^ 65	France	CS	323	75.9	65.5
Wu et al. (2023),^92^ 60	United Kingdom	LO (4 years)	473,184	57.0	56.0
Xiong et al. (2023),^93^ 36	United Kingdom	LO (5 years)	171,538	64.1	51.5
Xu et al. (2024),^94^ 33	United Kingdom	LO (12.6 years)	407,792	55.8	54.93
Yang et al. (2023),^95^ 17	United Kingdom	LO (12.4 years)	173,829	64.1	52.3
Yuan et al. (2023),^97^ 18	United Kingdom	LO (13.6 years)	462,524	58.0	45.8
Yuan et al. (2018),^59^ 45	China	CS	2617	69.1	45.9
Yu et al. (2020),^96^ 69	China	LO (13 years)	131,60	55–64	52.2
Zhao et al. (2015),^101^ 48	China	Case-controlled	404	Control:71.2 MCI: 84.6	Control: 76.3 MCI: 23.7
Zhang et al. (2023),^103^ 84	Japan	Intervention	9	79.2	77.8
Zhu et al. (2024),^106^ 34	China	LO (10 years)	7535	85.0	53.6

CS: Cross-Sectional; HIV: Human Immunodeficiency; LO: Longitudinal Observational; MCI: Mild Cognitive Impairment; N: Number.

**Table 2.** Summary of association between watching television with cognitive function.

Author (year), units, reference number, PW	Cognition		Adjusted Covariates	Results	Quality strength
	Domain	Tool			
Allen et al. (2017), (h/day/week),^104^ PW = 1	Episodic memory	Word list learning test	Age, sex, education level and ethnicity	**Cross-sectional** Watching more television is associated with lower memory performance: β = −0.068, p < 0.001 **Longitudinal** Watching more television is associated with lower memory performance: β = −0.029, p < 0.001	Good
Bakrania et al. (2018), (h/day),^105^ PW = 2	Episodic memory	Short-term numeric memory test	BMI, age, sex, ethnicity, social deprivation index, employment status, education level, number of cancers, number of noncancer illnesses, number of medications, smoking status, alcohol/drinking status, sleep duration, fruit and vegetable consumption, physical activity	**Cross-sectional** Prospective memory test: OR = 1.02 (99% CI: 1.00, 1.03), p = 0.001 Visual-spatial memory test: OR = 1.03 (99% CI: 1.02, 1.04), p < 0.001 Short-term numeric memory test: β = −0.09 (99% CI: −0.10, −0.07), p < 0.001 **Longitudinal** Visual-spatial memory test: OR = 1.02 (99% CI: 1.01, 1.04), p < 0.001 Short-term numeric memory test: OR = 1.07 (99% CI: 1.02, 1.12), p < 0.001	Strong
Cansino et al. (2024), (frequency),^35^ PW = 29	Working memory	MMSE	Age, years of education, vocabulary, MMSE score and BDI score.	**Verbal Discrimination:** B = 0.00/ SE B = 0.00/ β = 0.01 Reaction time: B = −0.50/ SE B = −0.59/ β = −0.02 **Spatial Discrimination:** B = 0.00/ SE B = 0.00/ β = 0.03 **Reaction time:** B = −0.17/ SE B = 0.59/ β = −001	Strong
Coelho et al. (2020), (h/weekday),^36^ PW = 41	Executive function	Behavioral Rating Inventory of Executive Function	Age and the LSI	GEC T score = 0.24, p = 0.05	Good
Fajersztajn et al. (2021), (h/day),^12^ PW = 6	Global cognition Working memory Executive function.	A list of 10 words CERAD battery and the CSI-D.	Cognitive performance at baseline, sociodemographic characteristics, and functional status.	**Immediate Memory:** Model 1: Coefficient = 0.02 (95% CI: −0.01, 0.05), p = 0.20 Model 2: Coefficient = 0.01 (95% CI: −0.02, 0.05), p = 0.40 **Verbal Fluency:** Model 1: Coefficient = −0.02 (95% CI: −0.10, 0.07), p = 0.60 Model 2: Coefficient = −0.02 (95% CI: −0.10, 0.07), p = 0.70	Strong
Fancourt et al. (2019), (h/day and h/weekend),^17^ PW = 7	Episodic memory Processing speed	Word learning task and animal naming task	Demographic Covariates and Health-Related Covariates.	**Verbal memory**: a linear relationship between television viewing and verbal memory (B = −0.13, SE = 0.04, p < 0.001, CI −0.20, −0.06) **Semantic fluency**: It was unclear whether there was a linear relationship between television viewing and semantic fluency (B = −0.13, SE = 0.07, p = 0.082, CI: −0.27, −0.02).	Strong
Hamer et al. (2014), (h/weekend),^66^ PW = 56	Global cognition	Three neuropsychological tests	Age, sex, smoking physical activity, alcohol, social class, disability, chronic illness, BMI, baseline CES-D score, and mutually for each sedentary behavior	**Daily TV viewing:** <2 h/d: coefficient = 0.60 (0.41, 0.78) 2 to <4 h/d: coefficient = 0.39 (0.26, 0.51) 4 to <6 h/d: coefficient = 0.20 (0.07, 0.33)	Good
Heisz et al. (2015), (frequency),^41^ PW = 46	Episodic memory	MoCA and American national adult reading test		TV viewing was negatively associated with episodic memory in older adults (B = −0.08)	Good
Hoang et al. (2016), (h/day),^68^ PW = 70	Processing speed and executive function	The Digit Symbol Substitution Test (DSST) and the Stroop test	Height, weight, BMI, hypertension, diastolic blood pressure, and diabetes mellitus.	Compared to participants with low television viewing, those with high television viewing over 25 years were more likely to exhibit poor cognitive performance on the DSST [OR = 1.64 (1.21, 2.23)] and Stroop test [OR = 1.56 (1.13, 2.14)].	Good
Jopp et al. (2007), (frequency),^43^ PW = 47	Episodic memory	A computer-controlled paired associate test of 30 unrelated word pairs	Education, health constraints, depressive affect, age	r = −0.02 (memory), r = 0.03 (speed), r = 0.04 (spatial), r = 0.01 (induct), r = 0.00 (fluency), r = −0.13* (verbal), r = −0.01 (general cog), r = 0.02 (memory concept), r = −0.15* (memory control), * p < 0.05	Strong
Karsazi et al. (2024), (h/week),^73^ PW = 57	Episodic memory working memory	Sepidar Cognitive Test (SCT) Battery, Corsi block-tapping test and the backward digit span memory task	Age, gender, education	**Episodic memory:** B = −0.001, SE = 0.001, p = 0.622 **Working memory:** B = −0.001, SE = 0.001, p = 0.082	Strong
Kesse-Guyot et al. (2012), (frequency),^24^ PW = 53	Executive function	A battery of 6 neuropsychological tests	Age, gender, supplementation group, education, occupational categories, retirement status, tobacco use status, MI, CES-D score, general health status, history of cardiovascular diseases, diabetes and hypertension, leisure- time physical activity, remaining sedentary behaviors.	**TV viewing and executive functioning:** **Cross-sectional:** T2: mean difference = −0.50, 95% CI: −1.43, −0.44, p = 0.03 T3: mean difference = −1.02, 95% CI −1.95, −0.10, p = 0.03 **Longitudinal:** Participants who increased their TV time between 2001 and 2007 showed better performance on executive functioning (mean difference = −0.36; 95% CI: −1.32, 0.59, p = 0.46) compared to those who decreased their TV time during that period.	Strong
Lin et al. (2022), (frequency),^109^ PW = 58	Global cognition and episodic memory	10-item Short Portable Mental Status Questionnaire (SPMSQ) and immediate word recall	Age, sex, marital status, occupation, residence, education, living arrangement, annual household income, satisfaction with one's economic status, and the number of chronic diseases.	**Global cognition** **Maintained cognition (vs. low):** Never: OR = 0.24 (0.12, 0.48), p < 0.001 Sometimes OR = 0.68 (0.39, 1.19), p = 0.177 **Episodic memory** Middle stable (vs. high declining): Never: OR = 0.17 (0.06, 0.49), p = 0.001 Sometimes OR = 0.43 (0.23, 0.81), p = 0.009 Improving (vs. high declining): Never: OR = 0.22 (0.07, 0.69), p = 0.009 Sometimes OR = 0.45 (0.21, 0.94), p = 0.033	Strong
Maasakkers et al. (2020), (h/weekend),^110^ PW = 71	Global cognition	MMSE and 3MS	Age, gender, ethnicity, education, income, BMI, morbidity count, perceived health, alcohol consumption, smoking status, marital status, living status, depression, sleep quality, blood pressure, and PA.	**Cross-sectional:** HELIAD: B = −0.028 (−0.092, 0.036), p = 0.40 **Longitudinal:** HELIAD: B = 0.028 (−0.021, 0.077), p = 0.26	Strong
Maasakkers et al. (2021), (h/week),^77^ PW = 4	Episodic memory	Four randomly selected word lists	Age, sex, education, depression, body mass index, morbidity count, smoking, alcohol consumption, subjective sleep quality, systolic and diastolic blood pressure, perceived health status, marital status, mobility	1. Every hour of objective SB was associated with a 0.01 (95% CI: −0.03, −0.00) decline on the MMSE per year in the fully adjusted model. 2. A worse decline in immediate recall over the preceding waves was related to slightly more TV time (B = −0.25 (95% CI: −0.48, −0.03) at the end of those four years.	Strong
Mai et al. (2023), (frequency),^78^ PW = 79	Global cognition	The Chinese version of the MMSE	Age, gender, current marital status, years of schooling, the total income of household last year, and current residence	Total effect of watching TV on global cognition: 0.281, p < 0.01	Good
Major et al. (2023), (h/day),^16^ PW = 5	Global cognition and processing speed	Teng Mini-Mental State Examination (3MS) and Digit Symbol Substitution Test (DSST)	Age, sex, race, and education level, smoking status body mass index, self-rated health), physical activity, and depressive symptoms	Participants who increased their TV watching time over 3 years had a significantly lower 3MS score (β = −1.45 ± 0.71; p < 0.05) at follow-up, compared with those who maintained a low level of TV time (referent).	Strong
Mellow et al. (2022), (h/day),^18^ PW = 42	Global cognition	Addenbrooke’s Cognitive Examination III	Age (years), sex (male, female), education (total years), and smoking status (current smoker, previous smoker, never smoker)	Global cognition: F(n,d) = 2.78(2372), p = 0.06, adj.p = 0.13	Good
Olanrewaju et al. (2020), (h/weekday),^10^ PW = 3	Episodic memory, executive function, and global cognition	10-word task list animal naming task and MMSE	age, sex, and social class, social participation, Physical activity, smoking, loneliness, alcohol and obesity, depression, disability, and chronic conditions.	**Cross-sectional** Verbal memory: 1.5–<2.5 h/day: 0.05 (−0.02, 0.13) 2.5–<3.5 h/day: 0.03 (−0.06, 0.12) >=3.5 h/day: −0.04 (−0.12, 0.04) h/day: −0.02 (−0.04, −0.003), p < 0.05 **Longitudinal (2 years follow-up)** Verbal memory: 1.5–<2.5 h/day: 0.01 (−0.09, 0.10) 2.5–<3.5 h/day: 0.001 (−0.10, 0.11) >=3.5 h/day: −0.03 (−0.14, 0.07) h/day: −0.001 (−0.02, 0.02) **Cross-sectional** Verbal fluency: 1.5–<2.5 h/day: 0.05 (−0.04, 0.13) 2.5–<3.5 h/day: 0.01 (−0.08, 0.09) >=3.5 h/day: −0.03 (−0.11, 0.06) h/day: −0.02 (−0.04, −0.002), p < 0.05 **Longitudinal (2 years follow-up)** Verbal fluency: 1.5–<2.5 h/day: −0.05 (−0.14, 0.05) 2.5–<3.5 h/day: −0.08 (−0.17, 0.02) >=3.5 h/day: −0.05 (−0.14, 0.05) h/day: −0.01 (−0.03, 0.01) **Cross-sectional** Global cognition: 1.5–<2.5 h/day: 0.07 (−0.01, 0.16) 2.5–<3.5 h/day: 0.04 (−0.05, 0.12) >=3.5 h/day: −0.01(−0.11, 0.10) h/day: −0.01 (−0.04, 0.01) **Longitudinal (2 years follow-up)** Global cognition: 1.5–<2.5 h/day: 0.03 -(0.07, 0.13) 2.5–<3.5 h/day: −0.04 (−0.15, 0.07) >=3.5 h/day: −0.02 (−0.14, 0.10) h/day: −0.01 (−0.04, 0.02)	Strong
Rosenberg et al. (2016), (h/weekday),^52^ PW = 51	Working memory	The Trial Making Test A & B	Age, gender, marital status, education status, device-measures physical activity	**No cognitive function association:** Trails A: Beta = −0.01 (SE = 0.01) p = 0.49 Trials B: Beta = −0.01 (SE = 0.02) p = 0.72 Trials A-B: Beta = −0.01 (SE = 0.03) p = 0.79	Strong
Shin et al. (2021), (h/week),^85^ PW = 8	Episodic memory	Immediate and delayed recall tests	Age, marital status, employment, household income, net worth, health insurance, ownership, number of children, self-reported health status, diagnoses of medical conditions, depression number of difficulties performing ADL and IADL, smoking, weight status, number of alcoholic drinks per week, and year-fixed effects	**Specification I:** Fluid Intelligence: Watching TV coefficient = 0.0015 (SE = 0.0130), z = 0.11 (−0.02, 0.03)	Strong
Tan et al. (2024), (h/day),^56^ PW = 63	Global cognition	A general cognitive ability score	Sociodemographic factors	Sedentary screen time (TV) h/d: effect estimate = −0.13 (p < 0.001)	Good
Wanders et al. (2021), (h/day),^88^ PW = 26	Global cognition	Validated Cognitive Online Self-Test Amsterdam (COST-A)	Age, sex, education level, BMI, working, smoking, alcohol consumption, health status, sleep disturbances, Geriatric Depression Scale, total MET-min/week, and other sedentary domains	T2: medium TV time: B = 0.03 (−0.01, 0.08), p = 0.15 T3: high TV time: B = 0.03 (−0.02, 0.08), p = 0.19	Strong
Wingood et al. (2024), (h/day),^25^ PW = 32	Executive function and episodic memory	Clock-drawing test and immediate and delayed recall	Age, race, ethnicity, education, physical function, and self-rated health	**Executive function:** Watching TV > 3h: SHR: 0.74 (0.34, 1.63), p = 0.46 **Episodic memory:** Watching TV > 3h: SHR: 0.43 (0.25, 0.75), p = 0.003	Strong
Yuan et al. (2018), (h/month),^59^ PW = 45	Executive function	MoCA	Age, gender, area, education, marital status), BMI, hypertension, diabetes, depression)	Modest TV watching (2∼4 /h/day) was conversely related to executive ability (OR (95% CI): 0.71 (0.52, 0.97)) for the elderly with a high education level	Strong

ADL: activities of daily living; BDI: Beck Depression Inventory; BMI: body mass index; B: beta-coefficient; β: standard beta-coefficient; CERAD: Consortium to Establish a Registry for Alzheimer's Disease; CES-D: Center for Epidemiologic Studies Depression; CI: confidence interval; CSI-D: Community Screening Instrument for Dementia; F: F-distribution; h/d hour/day; IADL: instrumental activities of daily living; LSI: Lifestyles Inventory; MET: Metabolic Equivalent of Task; MMSE: Mini-Mental State Examination; MoCA: Montreal Cognitive Assessment; OR: odds ratio; PA: physical activity; r: correlation coefficient; SE: standard error

*All the sedentary activities are measured by self-report.

**Table 3.** Summary of association between reading with cognitive function.

Author (year), units, reference number, PW	Cognition		Adjusted Covariates	Results	Quality strength
	Domain	Tool			
Cansino et al. (2024), (h/day),^35^ PW = 29	Working memory	MMSE	Age, years of education, vocabulary, MMSE score and BDI score	Verbal Discrimination: B = 0.00/ SE B = 0.00/ β = −0.01 Reaction time: B = −1.74/ SE B = 0.72/ β = −0.06 Spatial Discrimination: B = 0.00/ SE B = 0.00/ β = 0.10 Reaction time: B = −1.11/ SE B = 0.81/ β = −0.04	Strong
Cegolon et al. (2022), (h/day),^61^ PW = 14	Episodic memory and executive function	Rey’s Auditory Verbal and Learning Test (RAVLT) verbal fluency.	Age, gender, education and job situation, health, and social network variables	Reading would increase the memory score by about 0.080 SDs, and the verbal fluency score by about 0.069 SDs.	Good
Hamer et al. (2014), (h/day),^66^ PW = 56	Global cognition	Three neuropsychological tests	Age, sex, smoking physical activity, alcohol, social class, disability, chronic illness, BMI, baseline CES-D score, and mutually for each sedentary behavior	Reading daily newspaper (no): covariate adjusted coefficient = −0.06 (−0.16, 0.04)	Good
Ivleva et al. (2023), frequency,^71^ PW = 30	Episodic memory	A modified version of Rey’s Auditory Verbal Learning Test (RAVLT)	Age, gender, self-perceived health, country, years of education, leisure activities, and use of Internet	Beta (β) = 0.05, p < 0.001; B = 0.25 (−0.06, 0.56)	Good
Karsazi et al. (2024), (h/day),^73^ PW = 57	Episodic memory and working memory	Sepidar Cognitive Test (SCT) Battery, Corsi block-tapping test and the backward digit span memory task	Age, gender, education	**Episodic memory:** B = −0.007, SE = 0.005, p = 0.168 **Working memory:** B = −0.001, SE = 0.008, p = 0.991	Strong
Kesse-Guyot et al. (2012), (h/day),^24^ PW = 53	Executive function and verbal memory	A battery of 6 neuropsychological tests	Age, gender, supplementation group, education, occupational categories, retirement status, tobacco use status, BMI, CES-D score, general health status, history of cardiovascular diseases, diabetes and hypertension, leisure- time physical activity, remaining sedentary behaviors	**Executive function** **Cross-sectional:** T2: mean difference = −0.82, 95% CI: −1.73, 0.09, p = 0.06 T3: mean difference = −0.89, 95% CI: −1.81, 0.04, p = 0.06 **Longitudinal:** Changes in time spent reading over six years were not associated with executive functioning (mean difference = −0.61; 95% CI: −1.57, 0.36, p = 0.22). **Verbal memory** **Cross-sectional:** T2: mean difference = 0.57, 95% CI: −0.32, 1.47, p = 0.62 T3: mean difference = 0.54, 95% CI: −0.35, 1.43, p = 0.62 **Longitudinal:** Changes in time spent reading over six years were not associated with verbal memory (mean difference = 0.04; 95%CI: −0.92, 0.99, p = 0.94).	Strong
Lin et al. (2022), frequency,^109^ PW = 58	Global cognition and episodic memory	10-item Short Portable Mental Status Questionnaire (SPMSQ) and immediate word recall	Age, sex, marital status, occupation, residence, education, living arrangement, annual household income, satisfaction with one's economic status, and the number of chronic diseases	**Global cognition** **Maintained cognition (vs. low):** Never: OR = 0.16 (0.07, 0.36), p < 0.001 Sometimes OR = 4.94 (0.54, 45.46), p = 0.158 **Episodic memory** Middle stable (vs. high declining): Never: OR = 3.48 (2.48, 4.88), p < 0.001 Sometimes OR = 1.29 (0.84, 1.99), p = 0.241	Strong
Mao et al. (2020), frequency,^79^ PW = 15	Global cognition	MMSE	Age, sex, education level (year), body mass index, living pattern, residence, and current marital status, smoking status, alcohol consumption, regular exercise, regular fresh fruit consumption, and vegetable consumption; prevalence of diabetes mellitus, cerebrovascular disease, and heart disease, ADL, and housework	Compared with the participants who “never” engaged in reading books or newspapers, “almost every day” (HR: 0.64, 95% CI: 0.53, 0.78) was more strongly associated with a lower risk of cognitive impairment versus “sometimes” (HR: 0.82, 95% CI: 0.69, 0.98)	Strong
Major et al. (2023), (h/day),^16^ PW = 5	Global cognition and processing speed	Teng Mini-Mental State Examination (3MS) and Digit Symbol Substitution Test (DSST)	Age, sex, race, and education level, smoking status body mass index, self-rated health, physical activity, and depressive symptoms.	Reading time (β = 0.09 ± 0.03; p < 0.05 for 3MS score and β = 0.14 ± 0.04; p < 0.01 for DSST score)	Strong
Mai et al. (2023), (h/day),^78^ PW = 80	Global cognition	The Chinese version of the MMSE	Age, gender, current marital status, years of schooling, the total income of household last year, and current residence.	Total effect of reading on global cognition**:** 0.108, p < 0.01	Good
Shin et al. (2021), (h/day),^85^ PW = 8	Total cognition comprised of fluid and crystallized intelligence	Immediate and delayed recall tests, date and naming tasks	Age, marital status, employment, household income, net worth, health insurance ownership, number of children, self-reported health status, diagnoses of medical conditions, depression, number of difficulties performing ADL and IADL, smoking, weight status, number of alcoholic drinks per week, and year-fixed effects	**Total cognition:** Read papers/magazines Coefficient (SE) = 0.0188 (0.0326), p ≥ 0.05 z (95% CI) = 0.58 (−0.05, 0.08) Read books Coefficient (SE) = 0.1175 (0.0328), p < 0.001 z (95% CI) = 3.58 (0.05, 0.01) **Fluid intelligence:** Read papers/magazines Coefficient (SE) = 0.0147 (0.0308), p ≥ 0.05 z (95% CI) = 0.48 (−0.05, 0.08)	
				Read books Coefficient (SE) = 0.1095 (0.0310), p < 0.001 z (95% CI) = 3.54 (0.05, 0.17) **Crystallized intelligence:** Read papers/magazines Coefficient (SE) = 0.0139 (0.0073), p ≥ 0.05 z (95% CI) = 1.91 (0.00, 0.03) Read books Coefficient (SE) = 0.0140 (0.0070), p < 0.05 z (95% CI) = 2.00 (0.00, 0.03)	Strong
Wanders et al. (2021), (h/day),^88^ PW = 26	Global cognition	Validated Cognitive Online Self-Test Amsterdam (COST-A)	Age, sex, education level, BMI, working, smoking, alcohol consumption, health status, comorbidities/ polypharmacy, sleep disturbances, Geriatric Depression Scale, total MET-min/week, and other sedentary domains	T2: medium reading time: B = 0.03 (−0.02, 0.10), p = 0.22 T3: high reading time: B = 0.05 (−0.00, 0.10), p = 0.06	Strong

ADL: activities of daily living; B: beta-coefficient; BDI: Beck Depression Inventory; BMI: body mass index; β: standard beta-coefficient; CES-D: Center for Epidemiologic Studies Depression Scale; CI: confidence interval; HR: hazard ratio; IADL: instrumental activities of daily living; MET: Metabolic Equivalent of Task; MMSE: Mini-Mental State Examination; OR: odds ratio; SD: standard deviation; SE: standard error; z: standard score

*All the sedentary activities are measured by self-report.

**Table 4.** Summary of association between computer use with cognitive function.

Author (year), units, reference number, PW	Cognition		Adjusted Covariates	Results	Quality strength
	Domain	Tool			
Anaturk et al. (2021), (h/week),^31^ PW = 10	Global cognition	Test battery comprised of fluid intelligence, numeric/alphanumeric trail making, digit span, pairs matching, prospective memory, symbol digit matching, and simple reaction time	Age, sex, education, occupational status, assessment center, BMI, mean arterial pressure, frequency of alcohol intake, sleep duration, the presence of depressive or anxiety disorders and the number of individuals living in a household	Stable weekly computer use was linked to higher global cognitive performance, relative to stable low computer use (β = 0.62, 95% CI: 0.35, 0.89, FDR q = 1.16 × 10–4)	Good
Anaraky et al. (2024), (h/day),^60^ PW = 76	Episodic memory and executive function	Immediate and delayed recall tests and CDT	Age, gender, living arrangement, education, and race.	**Clock at t:** β = −0.042 (0.011), p < 0.001 **Immediate word recall at t:** β = −0.043 (0.009), p < 0.001	Good
Bakrania et al. (2018), (h/day),^105^ PW = 2	Episodic memory	Test battery designed for UK Biobank	BMI, age, sex, ethnicity, social deprivation index, employment status, education level, number of cancers, number of noncancer illnesses, number of medications, smoking status, alcohol/drinking status, sleep duration, fruit and vegetable consumption, physical activity.	**Cross-sectional:** Prospective memory test: OR = 0.92 (99% CI: 0.90, 0.94), p < 0.001 Visual-spatial memory test: OR = 0.92 (99% CI: 0.91, 0.93), p < 0.001 Short-term numeric memory test: β = 0.06 (99% CI = 0.04, 0.07), p < 0.001 **Longitudinal:** Visual-spatial memory test: OR = 0.96 (99% CI: 0.95, 0.98), p < 0.001 Short-term numeric memory test: OR = 0.92 (99% CI: 0.87, 0.98), p < 0.001	Strong
Bernstein et al. (2021), (h/day),^33^ PW = 67	Executive function and global cognition	Number span backward, Stroop color-word, TMT-B and z-scores		More total minutes spent using word processing was associated with better performance on executive function (r = 0.36, p < 0.01) and global cognition (r = 0.26, p < 0.05)	Good
Bernstein et al. (2022), (h/day),^32^ PW = 68	Episodic memory and executive function	Immediate and delayed recall, number span backward; Stroop color-word; and TMT-B		**Episodic memory:** Daily computer use: r = 0.11 **Executive function**: Daily computer use: r = 0.22	Adequate
Cansino et al. (2024), (h/day),^35^ PW = 29	Working memory	MMSE	Age, years of education, vocabulary, MMSE score and BDI score	**Verbal discrimination:** B = 0.00/ SE B = 0.00/ β = 0.07 **Reaction time:** B = −0.68/ SE B = 0.37/ β = −0.05 **Spatial discrimination:** B = 0.01/ SE B = 0.00/ β = 0.10 **Reaction time:** B = −0.86/ SE B = 0.41/ β = −0.05	Strong
Green et al. (2021), binary,^65^ PW = 22	Episodic memory	Delayed word recall test	Gender, age, country of survey, years of education, and years since retirement.	OLS estimates = 0.271 (0.095), p < 0.001	Good
Hamer et al. (2014), binary,^66^ PW = 56	Global cognition	Three neuropsychological tests	Age, sex, smoking physical activity, alcohol, social class, disability, chronic illness, BMI, baseline CES-D score, and mutually for each sedentary behavior	Use of internet (no): coefficient = −0.87 (−0.99, 0.76)	Good
Hartanto et al. (2020), (h/day),^67^ PW = 23	Executive function and episodic memory	Brief test of adult cognition by telephone	Age at assessment, sex, household income, education attainment, and subjective social status	**Executive functions**: estimate = 0.031 (p < 0.05) **Episodic memory**: estimate = −0.006	Strong
Hou et al. (2019), binary,^42^ PW = 80	Global cognition	MMSE	Age, gender, education level, marriage, family size, and log-transformed per capita household income.	**Video game participants:** M (SD) = 28.87 (1.64) **Non video game participants:** M (SD) = 27.72 (1.60) z = −1.38, p = 0.02	Poor
Ihle et al. (2020), ^70^ PW = 81	Working memory	TMT-B.		More frequent Internet use significantly correlated with a faster TMT accomplishment (i.e., better cognitive performance level, *r* = −0.22, *p* < 0.001, 95% *CI*: −5.51, −2.12 for corresponding raw estimate *b* of −3.78).	Good
Ivleva et al. (2023), (h/week),^71^ PW = 30	Episodic memory	A modified version of Rey’s Auditory Verbal Learning Test (RAVLT)	Age, gender, self-perceived health, country, years of education, leisure activities, and use of Internet.	Use of Internet in past 7 days with episodic memory: Beta (β) = −0.11, p < 0.001; B = −0.10 (−0.14, 0.06)	Good
Kesse-Guyot et al. (2012), (h/day),^24^ PW = 53	Executive function and verbal memory	A battery of 6 neuropsychological tests	Age, gender, supplementation group, education, occupational categories, retirement status, tobacco use status, BMI, CES-D score, general health status, history of cardiovascular diseases, diabetes and hypertension, leisure- time physical activity, remaining sedentary behaviors.	**Verbal memory** **Cross-sectional:** T2: mean difference = 1.53, 95% CI: 0.58, 2.48, p < 0.001 T3: mean difference = 1.86, 95% CI 0.95, 2.77, p < 0.001 **Longitudinal:** Increases in time spent using a computer over six years were associated with verbal memory (mean difference = 1.41; 95% CI: 0.55, 2.27, p = 0.001). **Executive function** **Cross-sectional:** T2: mean difference = 0.47, 95% CI: −0.49, 1.44, p < 0.0001 T3: mean difference = 2.15, 95% CI 1.22, 3.08, p < 0.0001 **Longitudinal:** Increases in time spent using a computer over six years were associated with executive functioning (mean difference=1.41; 95% CI: 0.53, 2.28, p = 0.002).	Strong
Kim et al. (2022), h/day),^45^ PW = 72	Working memory	Spatial span task		**Early middle-aged nongame group:** 5.25 (0.38) **Early middle-aged game group**: 5.93 (0.18)	Adequate
Kurita et al. (2021), binary,^76^ PW = 66	Executive function and processing speed	TMT-B and Symbol Digit Substitution Task (SDST)	Age, sex, years of education, hypertension, diabetes, hyperlipidemia, and heart disease.	The computer use group had a reduced adjusted odds ratio (aOR) of cognitive decline, after adjustment for covariates, in both the complete and imputed samples (complete samples: aOR = 0.71, 95% CI: 0.52, 0.97, p = 0.030; imputed samples: aOR = 0.67, 95% CI: 0.51, 0.88, p < 0.003).	Good
Mariano et al. (2021), (h/day),^80^ PW = 25a	Episodic memory	Two free recall tasks	Age, education, gender, marital status, employment status, income, subjective health, physical conditions, depressive symptoms, and loneliness	The autoregressive associations of self-perceptions of aging (*b* = 0.76, *p* < 0.001), cognitive functioning (*b* = 0.67, *p* < 0.001), and computer use behavior (*b* = 0.79, *p* < 0.001) were all significant, substantial, and positive.	Strong
Mariano et al. (2021), (h/day),^80^ PW = 25b	Processing speed	Digit Symbol Substitution Test	Age, education, gender, marital status, employment status, income, subjective health, physical conditions, depressive symptoms, and loneliness	The autoregressive associations of self-perceptions of aging (b = 0.82, p < 0.001), cognitive functioning (b = 0.73, p < 0.001), and computer use behavior (b = 0.79, p < 0.001) were all significant, substantial, and positive	Strong
Mariano et al. (2021), binary,^81^ PW = 64	Global cognition	Digit Symbol Substitution Test	Age, gender, marital status, occupational status, living arrangements, region, education, and income	General internet use (T1) was significantly and positively associated with cognitive and physical functioning (β = 0.031, b = 0.005, p = 0.018 and β = 0.030, b = 0.016, p = 0.017, respectively).	Strong
Ringin et al. (2023), binary, ^51^ PW = 35	Processing speed	A brief computerized battery	Sex, education, SES, sleep duration, alcohol use status, smoking status, and occupational status	There was a main effect of non-occupational computer use, with cognitive scores increasing by 0.13 (0.12, 0.15) for every 1-h increase in computer use. SE = 0.008 p < 0.0001	Good
Shin et al. (2021), (h/day),^85^ PW = 8	Total cognition comprised of fluid and crystallized intelligence	Immediate and delayed recall tests, date and naming tasks	Age marital status, employment, household income, net worth, health insurance ownership, number of children, self-reported health status, diagnoses of medical conditions, Depression number of difficulties performing ADL and IADL, smoking, weight status, number of alcoholic drinks per week, and year-fixed effects	**Total cognition** Coefficient (SE) = 0.1024 (0.0237), p < 0.001 z (95% CI) = 4.31 (0.06, 0.15) **Fluid intelligence:** Coefficient (SE) = 0.0883 (0.0224), p < 0.001 z (95% CI) = 3.94 (0.04, 0.13) **Crystallized intelligence:** Coefficient (SE) = 0.0171 (0.0048), p < 0.001 z (95% CI) = 3.59 (0.01, 0.03)	Strong
Shi et al. (2023), binary,^53^ PW = 73	Global cognition	Vocabulary measurement, numeracy measurement, and self-rated level of remembering important things	Age, gender, education level, marriage, family size, and places surveyed.	**No internet usage:** Estimate = 0.024, p = 0.001 **Internet usage:** Estimate = 0.013, p = 0.001	Good
Tan et al. (2024), (h/day),^56^ PW = 63	Global cognition	A general cognitive ability score	Sociodemographic factors	Effect estimate = 0.21 (p < 0.001)	Good
Wanders et al. (2021), (h/day),^88^ PW = 26	Global cognition	Validated Cognitive Online Self-Test Amsterdam (COST-A)	Age, sex, education level, BMI, working, smoking, alcohol consumption, health status, comorbidities/polypharmacy, sleep disturbances, Geriatric Depression Scale, total MET-min/week, and other sedentary domains.	T2: medium computer time: B = 0.04 (−0.00, 0.09), p = 0.07 T3: high computer time: B = 0.07 (0.02, 0.12), p = 0.01	Strong
Woods et al. (2023), (h/day),^100^ PW = 59	Episodic memory, executive function, and processing speed	Prospective and Retrospective Memory Questionnaire (PRMQ) and the Profile of Mood States (POMS)	Education and BCUAQ computer-related anxiety	Findings showed medium-sized, significant correlations for: memory (*r* = 0.30, *p* < 0.001) executive functions (*r* = 0.27, *p* < 0.001) domains, with smaller effect sizes for: motor (*r* = 0.12, *p* = 0.044) processing speed (*r* = 0.21, *p* < 0.001), attention (*r* = 0.17, *p* = 0.005) domains.	Good
Wu et al. (2019), (h/day),^58^ PW = 65	Global cognition, working memory, and processing speed	MMSE Digit span backward TMT-A	Age, education, and clinical diagnosis	**Computer daily use only:** MMSE: 27.3 (2.62), F = 4.328, p = 0.005 Digit span backward: 4.23 (1.04), F = 1.211, p = 0.306 TMT-A: 41.6 (12.3), F = 5.100, p = 0.002	Good
Yu et al. (2020), binary,^96^ PW = 69	Episodic memory	A word recall test	Age, gender, marital status, educational level, annual household, and health factors.	**Internet use (2015):** Cognitive score: coefficient: 0.30, p < 0.01	Strong

ADL: activities of daily living; BDI: Beck's Depression Inventory; BMI: body mass index; B: beta-coefficient; CES-D: Center for Epidemiologic Studies Depression; CI: confidence interval; CDT: Center for Doctoral Training; FDR: false discovery rate; F: F-distribution; HR: hazard ratio; ICD-9: International Classification of disease 10^th^ Revision; IADL: instrumental activities of daily living; MET: Metabolic Equivalent of Task; MMSE: Mini-Mental State Examination; M: mean; OR: odds ratio; r: correlation coefficient; SD: standard deviation; SES: socioeconomic status; TMT: Trail Making Test; z: standard score

*All the sedentary activities are measured by self-report, except one was measured by monitoring software.^32^

**Table 5.** Summary of association between playing games and puzzles with cognitive function.

Author (year), units, reference number, PW	Cognition		Adjusted Covariates	Results	Quality strength
	Domain	Tool			
Brooker et al. (2019), frequency,^34^ PW < 74	Working memory	The PROTECT Cognitive-Test Battery (PCTB)	Age, gender, education, and the number of times the tasks had been performed	Cognitive performance in the group who never performed number puzzles was notably poorer than in all other groups, with Cohen's *d* effect sizes for paired-associate learning ranging from 0.23 to 0.41, for digit span from 0.17 to 0.29, for spatial working memory from 0.28 to 0.47, and for verbal reasoning from 0.21 to 0.4.	Adequate
Brooker et al. (2019), frequency,^107^ PW < 75	Working memory	Spatial Working Memory (SWM)	Age, gender, education, and the number of times the tasks had been performed	For each task, the group who never performed word puzzles was significantly inferior to each other group (all p < 0.00001). The group who occasionally performed word puzzles was significantly poorer on each task than the three groups who performed puzzles weekly or more (p < 0.0294 to p < 0.00001), with effect sizes ranging from 0.05 to 0.25.	Adequate
Cegolon et al. (2022), (h/week),^61^ PW = 14	Episodic memory and executive function	Rey’s Auditory Verbal Learning Test (RAVLT) verbal fluency	Age, employment status, partnership status, and health (physical activity and alcohol consumption)	Doing word or number games (such as crosswords or Sudoku) and playing cards or chess have a similar impact on verbal fluency (respectively 0.053 SDs and 0.038 SDs) but very different effects on memory score (respectively 0.069 SDs and 0.026 SDs)	Good
Cutting et al. (2023), frequency,^38^ PW < 37	Working memory	Online WM task		The superior WMC for older adults in the puzzle group meant that equivalent WMC was seen for younger and older adults in this game group (t (58) < 1.08, p = 0.285, η2 < 0.29)	Adequate
Ivleva et al. (h/day),^71^ (2023), PW < 30	Episodic memory	A modified version of Rey’s Auditory Verbal Learning Test (RAVLT)	Age, gender, self-perceived health, country, years of education, lei- sure activities, and use of Internet.	Beta (β) < 0.00, B < 0.01 (−0.18, 0.19)	Good
Mao et al. (2020), frequency,^79^ PW < 15	Global cognition	MMSE	Age, sex, education level (year), body mass index, living pattern, residence, and current marital status, smoking status), alcohol consumption, regular exercise, regular fresh fruit consumption, and vegetable consumption; prevalence of diabetes mellitus, cerebrovascular disease, and heart disease, ADL, and housework	Compared with “never” engaging in playing cards or mahjong, “almost every day” (HR: 0.70, 95% CI: 0.56; 0.86) was similarly associated with a reduced risk of cognitive impairment versus “sometimes” (HR: 0.69, 95% CI: 0.60, 0.81).	Strong
Mai et al. (2023), (h/week),^78^ PW < 79	Global cognition	The Chinese version of the MMSE	Age, gender, current marital status, years of schooling, the total income of household last year, and current residence	Total effect of playing cards game on global cognition: 0.069, p < 0.01	Good
Schaham et al. (2017), session/intervention,^54^ PW = 85	Executive function	TMT-B		Puzzle Retreat: r < −0.61, p < 0.000; One Touch Drawing: r < 0.39, p < 0.02; Chocolate Fix: r < 0.39, p < 0.02.	Good
Shin et al. (2021), frequency,^85^ PW < 8	Episodic memory	Immediate and delayed recall tests	Age (at 50), marital status, employment, household income, net worth, health insurance ownership, number of children, self-reported health status, diagnoses of medical conditions, Depression number of difficulties performing ADL and IADL, smoking, weight status, number of alcoholic drinks per week, and year-fixed effects	**Total cognition** Coefficient (SE) < 0.1864 (0.0501), p < 0.001 z (95% CI) < 3.72 (0.09, 0.28) **Fluid intelligence:** Coefficient (SE) < 0.1747 (0.0475), p < 0.001 z (95% CI) < 3.68 (0.08, 0.27) **Crystallized intelligence:** Coefficient (SE) < 0.0207 (0.0111), p ≥ 0.05 z (95% CI) < 1.87 (0.00, 0.04)	Strong
Shuai et al. (2023), frequency,^55^ PW < 9	Global cognition	Chinese version of the MMSE	City, sex, age, education, marital status, annual income, living alone, BMI, drinking status, smoking status, self-rated sleeping quality, physical activity, hypertension, diabetes, and depression status	The participants who had play cards (or mahjong) sedentary duration had higher MMSE scores (β<1.132, 95% CI: 0.788, 1.476).	Good
Zhang et al. (2023), minutes/intervention,^103^ PW = 84	Global cognition	MMSI, MoCA-Japanese, TMT		Total MMSE Score: T1: 21.2 ± 3.0, T2: 20.8 ± 1.9, z < 0.300, p = 0.764 MoCA-J Score: T1: 18.7 ± 2.8, T2: 18.0 ± 3.0, z < 0.839, p = 0.401 TMT-B/A Score: T1: 3.0 ± 1.6, T2: 2.9 ± 1.4, z < 0.533, p = 0.594	Poor
Zhu et al. (2024), (h/day),^106^ PW < 34	Global cognition	MMSE	Sex, marital status, schooling years, residence, financial support, smoking status and alcohol consumption (drinking, no drinking)	Older people who play mahjong occasionally in 2011 and 2014 (*β* = 0.062, 0.055, p < 0.01) and those who played mahjong daily in 2011 and 2018 (*β* = 0.056, 0.067, p < 0.05) had higher scores in reaction ability. Older people who played mahjong occasionally and daily had higher scores in attention and calculation, recall, and self-coordination abilities in 2011, 2014 and 2018 (*p* < 0.01).	Strong

ADL: activities of daily living; B: beta-coefficient; CI: confidence interval; HR: hazard ratio; IADL: instrumental activities of daily living; MMSE: Mini-Mental State Examination; MoCA: Montreal Cognitive Assessment; OR: odds ratio; SD: standard deviation; SE: standard error; TMT: Trail Making Test; WM: working memory; WMC: working memory capacity; z: standard score.

*All the sedentary activities are measured by self-report.

**Table 6.** Summary of association between driving with cognitive function.

Author (year), units, reference number, PW	Cognition		Adjusted Covariates	Results	Quality strength
	Domain	Tool			
Bakrania et al. (2018), (h/day),^105^ PW = 2	Episodic memory	Short-term numeric memory test	BMI, age, sex, ethnicity, social deprivation index, employment status, education level, number of cancers, number of noncancer illnesses, number of medications, smoking status, alcohol/drinking status, sleep duration, fruit and vegetable consumption, and physical activity.	**Cross-sectional:** Prospective memory test: OR = 1.15 (99% CI: 1.13, 1.17), p < 0.001 Visual-spatial memory test: OR = 1.06 (99% CI: 1.05, 1.07), p < 0.001 Short-term numeric memory test: B = 0.06 (99% CI: 0.04, 0.07), p < 0.001 **Longitudinal:** Visual-spatial memory test: OR = 1.02 (99% CI: 1.01, 1.04), p < 0.001 Short-term numeric memory test: OR = 1.07 (99% CI: 1.02, 1.12), p < 0.001	Strong
Miller et al. (2024), (h/day),^48^ PW = 31	Episodic memory, global cognition, processing speed, working memory	Immediate and delayed recall trials, Symbol Digit Modalities Test (SDMT)	Life space, IADLs, depressive symptoms, sex, race, ethnicity, level of education, and SES.	Global cognition: B = −0.007 p < 0.05 Episodic memory: B = −0.009 p < 0.005 Working memory: B = −0.004 Processing speed: B = −0.007 p < 0.005	Strong

BMI: body mass index; B: beta-coefficient; CI: confidence interval; IADLs: instrumental activities of daily living; OR: odds ratio; SES: socioeconomic status

*All the sedentary activities are measured by self-report.

**Table 7.** Summary of association between individual sedentary activities with dementia.

Author (year), units, reference number, PW	Sedentary behavior	Tool	Adjusted Covariates	Results	Quality strength
Chen et al. (2021), (frequency),^108^ PW = 12	Watching television	The Tokyo Metropolitan Health and Longevity Medical Center Research Institute developed an exclusive checklist for the dementia diagnosis.	Age, sex, married, senior high school, working and diabetes.	Compared to the reference category (watching TV almost every day), correlation coefficients were: Never/hardly = 1.351, p < 0.01 Several times per year = 2.847, p < 0.01 Several times per month = 1.516, p < 0.01 Several times per week = 0.394, p < 0.05	Strong
Fajersztajn et al. (2021), (h/day),^12^ PW = 6	Watching television	A list of 10 words CERAD battery and the CSI-D	Cognitive performance at baseline, sociodemographic characteristics, and functional status	OR (95% CI) = 0.82 (0.63, 1.04), p = 0.08	Strong
Floud et al. (2021), (h/day),^64^ PW = 49	Reading	Hospital record	Education, area deprivation, frequency of strenuous physical activity, BMI, smoking, alcohol consumption, and use of menopausal hormones.	The RR was 3.18 (99% CI 2·61, 3·88) during years 0–4 of follow-up but declined substantially with follow-up duration and was 1.13 (0.98, 1.30) the second decade of follow-up	Good
Janoutová et al. (2021), (h/day),^102^ PW = 82	Reading and crosswords	The International Classification of Diseases, 10th Revision		There were statistically significant differences in reading and solving crosswords between case and control groups	Adequate
Jia et al. (2024), frequency,^72^ PW = 83	Computer	ICD-9 and ICD-10 codes	Demographic and socioeconomic factors, lifestyle behaviors, and medical history	High frequency of playing computer games was associated with decreased risk of incident dementia (HR, 0.81 [95% CI: 0.69, 0.94]).	Strong
Lindstrom et al. (2005), (h/day),^98^ PW = 52	Watching television	Evaluated by neuropsychological, laboratory and neurological examination.	Socio-demographic characteristics	Each hour increases in television viewing in middle-adulthood corresponded to a 1.3 times greater risk of being in the case group (*p* = 0.008).	Good
Nemoto et al. (2022), (categorical),^82^ PW = 19	Watching television	Long-term Care Insurance data	Sex, age, years of education, marital status, living status, and employment status, self-rated health, self-reported medical conditions, and frailty.	The cumulative incidence of dementia was the highest in the low physical activity category (17.5%; 95% CI: 15.7, 19.0), the low mentally active sedentary behavior category (13.8%; 95% CI: 12.0, 15.9), and the high passive sedentary behavior category (13.7%; 95% CI: 12.0, 15.5)	Strong
Raichlen et al. (2022), (frequency),^83^ PW = 44	Watching television	Hospital inpatient records were used to determine all-cause dementia diagnoses.	Age, education, socioeconomic status, the presence of the *APOE* ε4 allele, ethnicity, chronic conditions, smoking status, alcohol use, BMI, depression, adherence to a healthy diet, hours of sleep.	The highest tertile of TV viewing time was associated with increased dementia risk relative to low TV time: HR (95% CI = 1.28 (1.18, 1.39)	Good
Raichlen et al. (2022), (h/day),^83^ PW = 44	Computer use	Hospital inpatient records were used to determine all-cause dementia diagnoses.	Age, education, socioeconomic status, the presence of the *APOE* ε4 allele, ethnicity, chronic conditions, smoking status, alcohol use, BMI, depression, adherence to a healthy diet, hours of sleep.	Both medium and high computer time were associated with reduced risk of incident dementia HR (95% CI): medium computer use = 0.63 (0.58, 0.68) high computer use = 0.70 (0.65, 0.76)	Good
Shimada et al. (2018),^54^ (h/day), PW = 62	Playing card games and puzzles, reading, computer use and driving	Data were collected from the Japanese Health Insurance System	Age, sex, educational level, current smoking, chronic medical illnesses, depressive symptoms, comfortable walking speed and cognitive impairment.	Card games and puzzles: HR = 0.90 (0.67, 1.20), p = 0.457 Reading: HR = 0.83 (0.48, 1.44), p = 0.511 Computer use: HR = 1.15 (0.80, 1.66), p = 0.439 Driving: HR = 0.63 (0.45, 0.88), p = 0.007	Good
Takeuchi et al. (2022), (categorical),^86^ PW = 16	Non-occupational computer use	Hospital inpatient records and connections to death registry data	The neighborhood-level socioeconomic status at recruitment, education level at recruitment, household income, current employment status, metabolic equivalent of task hours, the number of people in the household, height, body mass index, self-reported health status, duration of sleep, systolic blood pressure, current alcohol drinking level, current tobacco smoking level, race, diagnosis of diabetes, heart attack, angina, stroke, cancer, and other severe medical conditions, driver jobs, and visuospatial memory task performance	Compared to those with 0 h non-occupational computer use, groups showed decreased risk, HR (95% CI): ≤1h = 0.626 (0.538, 0.728), p < 0.001 2–3h = 0.722 (0.601, 0.869), p = 0.001 ≥4h = 0.653 (0.476, 0.897), p = 0.009	Strong
Takeuchi et al. (2023), (h/day),^87^ PW = 21	Watching television	Hospital inpatient records and connections to death registry data	The neighborhood-level socioeconomic status at recruitment, education level at recruitment household income, current employment status, metabolic equivalent of task hours, number in household, body mass index, self-reported health status, and sleep duration	Increasing length of TV viewing by 1 h is associated with dementia: HR = 1.049 (CI: 1.027, 1.072), p < 0.001	Strong
Takeuchi et al. (2022), (categorical),^86^ PW = 16	Driving	Hospital inpatient records and connections to death registry data	The neighborhood-level socioeconomic status at recruitment, education level at recruitment, household income, current employment status, metabolic equivalent of task hours, the number of people in the household, height, body mass index, self-reported health status, duration of sleep, systolic blood pressure, current alcohol drinking level, current tobacco smoking level, race, diagnosis of diabetes, heart attack, angina, stroke, cancer, and other severe medical conditions, driver jobs, and visuospatial memory task performance	Compared to those with 0 h driving, groups showed increased risk, HR (95% CI): ≤1h = 1.544 (1.324, 1.801), p < 0.001 2–3h = 1.574 (1.307, 1.895), p < 0.001 ≥4h = 1.525 (1.023, 2.274), p = 0.038	Strong
Tarawit et al. (2021), (Binary),^57^ PW = 54	Computer use	MMSE-Thai 2002	Gender, occupation, marital status, education level, BMI, chronic disease and regular medication	B = 2.393, Coefficients SE = 0.702, β = 0.170, t = 3.324, p = 0.001	Good
Wu et al. (2023), (frequency),^91^ PW = 27	Computer use	Regular study visits	Age, sex, race and ethnicity, education, socioeconomic status, living situation, smoking status, alcohol intake, physical activities, body mass index, hypertension, diabetes, dyslipidemia, depression and Fried frailty phenotype	Increasing the frequency of participation in adult literacy (education classes, computer usage, and writing letters and journals) by 1 category (e.g., from sometimes to often) was associated with an 11.0% reduction in dementia risk (adjusted hazard ratio [AHR], 0.89 [95% CI, 0.85, 0.93]; p < 0.001).	Strong
Wu et al. (2023), (frequency),^91^ PW = 27	Playing games and puzzles	Regular study visits	Age, sex, race and ethnicity, education, socioeconomic status, living situation, smoking status, alcohol intake, physical activities, body mass index, hypertension, diabetes, dyslipidemia, depression, and Fried frailty phenotype	More frequent participation in active mental activities (playing games, cards, or chess or doing puzzles and crosswords) was associated with a 9.0% reduction in the risk of dementia (AHR, 0.91 [95% CI, 0.87, 0.96]; p < 0.001)	Strong
Wu et al. (2023), (frequency),^92^ PW = 60	Computer use and watching television	Inpatient records	Socio-demographic information, lifestyle factors, and anthropometric measurements	1. For moderate versus the lowest computer uses, the adjusted HRs (95% CIs) = 0.68 (0.64, 0.72) for dementia. 2. The multivariable HRs (95% CIs) for the highest versus the lowest group of TV viewing time were 1.28 (1.17, 1.39) for dementia.	Good
Xiong et al. (2023), (h/day),^93^ PW = 36	Watching television	Linkage to hospital admission records and death registry data	Sex, ethnicity, education, socioeconomic deprivation, depression, *APOE* ε4 carrier status, cardiometabolic disease status, and cognitive performance	< 4 h per day of television viewing time: HR = 0.87 (0.81, 0.92), p < 0.001	Good Strong
Xu et al. (2024), (categorical),^94^ PW = 33	Computer use	Self-reported data and hospital inpatient records	Age, sex, ethnicity, deprivation index, education attainment, employment status, BMI, smoking status, frequency of alcohol consumption, depressive mood, sleep duration, history of hypertension, history of diabetes, physical activity, fluid intelligence, prospective memory, and reaction time	>5 h/d: HR = 0.86 (0.66, 1.12), p = 0.402	
Yang et al. (2023), (categorical),^95^ PW = 17	Watching television	Hospital inpatient records and connections to death registry data	Age, sex, race, educational levels, Townsend Deprivation Index, smoking status, alcohol consumption, family history of dementia, and medication usage for diabetes, hypertension, and high cholesterol	Compared with participants who spent >0-<2 h/day watching TV, the adjusted HRs (95% CIs) of new-onset all-cause dementia were 1.29 (1.06, 1.57), p = 0.11 for 0 h/day and 1.22 (1.11, 1.35), p < 0.001 for ≥2 h/day	Strong
Yang et al. (2023), (categorical),^95^ PW = 17	Driving	Hospital inpatient records and connections to death registry data	Age, sex, race, educational levels, Townsend Deprivation Index, smoking status, alcohol consumption, family history of dementia, and medication usage for diabetes, hypertension, and high cholesterol	Compared with spending >0-<2 h/day driving, the adjusted HRs (95% CIs) of new-onset all-cause dementia were 1.41 (1.32, 1.51), p < 0.001 for 0 h/day and 1.33 (1.22, 1.44), P < 0.001 for ≥2 h/day	Strong
Yang et al. (2023), (categorical),^95^, PW = 17	Computer use	Hospital inpatient records and connections to death registry data	Age, sex, race, educational levels, Townsend Deprivation Index, smoking status, alcohol consumption, family history of dementia, and medication usage for diabetes, hypertension, and high cholesterol	Compared with spending >0-<2 h/day in non-occupational computer use, the adjusted HRs (95% CI) of new-onset all-cause dementia were 1.42 (1.32, 1.52), p < 0.001 for 0 h/day and 1.20 (1.11, 1.30), p < 0.001 for ≥ 2 h/day	Strong
Yuan et al. (2023), (categorical),^97^ PW = 18	Computer use	UK Biobank database algorithmically defined outcomes and the records of the outcome of the hospital diagnosis	Age, sex, BMI, education level, Townsend deprivation index (TDI), ethnicity, myocardial infarction, alcohol use, hypertension, smoking, diabetes, and stroke	Compared to ≤1 h of computer use the following associations were observed HR (95% CI): **All cause-dementia** 2–3 h = 0.96 (0.90, 1.02), p = 0.21 ≥4 h = 1.00 (0.89, 1.12), p = 0.99 **Alzheimer’s Disease** 2–3 h = 0.90 (0.81, 1.00), p = 0.051 ≥4 h = 0.96 (0.79, 1.16), p = 0.65 **Vascular Dementia** 2–3 h = 0.93 (0.81, 1.07), p = 0.31 ≥4 h = 1.11 (0.88, 1.39), p = 0.37	Good
Yuan et al. (2023), (categorical),^97^ PW = 18	Watching television	UK Biobank database algorithmically defined outcomes and the records of the outcome of the hospital diagnosis	Age, sex, BMI, education level, Townsend deprivation index (TDI), ethnicity, myocardial infarction, alcohol use, hypertension, smoking, diabetes, and stroke	Compared to ≤1 h of watching television the following associations were observed HR (95% CI): **All cause-dementia** 2–3 h = 1.09 (1.01, 1.18), p < 0.05 ≥4 h = 1.29 (1.19, 1.40), p < 0.001 **Alzheimer’s Disease** 2–3 h = 1.12 (0.99, 1.26), p = 0.08 ≥4 h = 1.25 (1.10, 1.42), p < 0.001 **Vascular Dementia** 2–3 h = 1.12 (0.94, 1.33), p = 0.202 ≥4 h = 1.24 (1.04, 1.49), p < 0.05	

*APOE*: Apolipoprotein E; BMI: body mass index; RR: relative ratio; CERAD: Consortium to Establish a Registry for Alzheimer’s Disease; CSI-D: Community Screening Instrument for Dementia; CI: confidence interval; HR: hazard ratio; OR: odds ratio

*All sedentary activities are measured by self-report.

**Table 8.** Summary of association between individual sedentary activities with cognitive impairment.

Author (year), units, reference number, PW	Sedentary activities	Tool	Adjusted Covariates	Results	Quality strength
Da Ronch et al. (2015), (categorical),^39^ PW = 50	Watching television	MMSE	Gender, age, study center, level of education, living status, work status, level of functioning, number of physical illnesses, physical activity	MMSE and TV viewing inversely related beta = −0.105 (B 95%CI −38.76 to −13.49), p < 0.001	Good
Feng et al. (2020), (binary),^63^ PW = 77	Reading, watching television, and computer use	(i)immediate recall of a 10-word list, (ii) counting backward from 20 to 1, (iii) serial 7 subtraction, and (iv) delayed recall of a 10-word list	Age, sex, smoking, drinking, ethnicity, education levels, married status, regions, gross family income, hypertension history, diabetes history, and death	1. Reading was associated with lower incidence of MCI: HR (95% CI), p = 0.718 (0.548, 0.940), p = 0.016 2. TV or computer usage was associated with higher incidence of MCI: HR (95% CI), p = 1.455 (1.040, 2.036), p = 0.029	Good
Fajersztajn et al. (2021), (h/day),^12^ PW = 6	Watching television	A list of 10 words CERAD battery and the CSI-D	Cognitive performance at baseline, sociodemographic characteristics, and functional status	OR = 1.02 (95% CI: 0.84, 1.24), p = 0.80	Strong
Jung et al. (2020), (h/day),^44^ PW = 39	Watching television	MMSE for Dementia Screening	Engagement in cognitively beneficial leisure activities, social network and social participation, health condition, health-related behaviors, and sociodemographic factors	The OR (95% CI) = 1.94 (1.18, 3.21), p < 0.01 for individuals in the viewer group approximately doubled compared with that calculated before PS matching.	Strong
Kurita et al. (2019), (binary),^46^ PW = 28	Computer use	National Center for Geriatrics and Gerontology - Functional Assessment Tool	Age, sex and education level), whether employed (yes/no), instrumental activities of daily living limitation, the number of chronic diseases (hypertension, diabetes and hyperlipidemia) and depressive symptoms	OR (95% CI) = 0.33 (0.24, 0.47)	Good
Krell-Roesch et al. (2017), (frequency),^75^ PW = 24	Computer use	Neurological evaluation, risk factor assessment interview, and neuropsychological testing	Sex, age, and educational level	HR (95% CI) = 0.70 (0.57, 0.85)	Strong
Krell-Roesch et al. (2019), (frequency),^74^ PW = 78	Reading and computer use, playing games	A neurologic examination, risk factors ascertainment, and neuropsychological testing	Age, sex, education, and *APOE* ε4 genotype status	Compared to reading less than once a month, reading everyday: HR = 0.72 (0.57, 0.91), p < 0.01 Compared to using a computer less than once a month, using a computer every day: HR = 0.67 (0.55, 0.83), p < 0.01 Compared to playing games less than once a month, playing games every day: HR = 0.61 (0.48, 0.78), p < 0.01	Strong
Nemoto et al. (2018), (categorical),^49^ PW = 11	Watching television	Kihon Checklist (KCL)	Sex, age, educational attainment, residential status, alcohol status, and smoking status, medical history, stress, loss-event experience, and depression	**Cognitive complaints** OR (95% CI), p <1 h/day = reference 1–2 h/day = 1.21 (1.00, 1.47), p = 0.05 2–3 h/day = 1.00 (0.83, 1.22), p = 0.97 ≥3 h/day = 1.09 (0.90, 1.32), p = 0.36	Strong
Nemoto et al. (2018), (categorical),^49^ PW = 11	Reading (books or newspapers)	Kihon Checklist (KCL)	Sex, age, educational attainment, residential status, alcohol status, and smoking status, medical history, stress, loss-event experience, and depression	Cognitive complaints *adjusted OR (95% CI), p <10 min/day = reference 10–20 min/day = 0.63 (0.53, 0.75), p < 0.01 20–30 min/day = 0.59 (0.49, 0.71), p < 0.01 ≥30 min/day = 0.47 (0.39, 0.57), p < 0.01	Strong
Rawtaer et al. (2020), (frequency),^50^ PW = 38	Watching television	MMSE, MoCA, CDR, and neuropsychological test performance and via a consensus panel		There was no difference in television viewing time (min/d) between people with MCI: 219 ± 220 and those who were cognitively healthy: 174 ± 176, p = 0.52	Adequate
Ramos et al. (2021), (h/day),^99^ PW = 43	Watching television	Impairment Screen (MIS) test, Spanish version of Short Portable Mental State Questionnaire (SPMSQ), and Semantic Verbal Fluency (SVF) test.	Gender, age, history of dementia, SMC, educational level, marital status, feelings of depression and daily internet usage	OR = 1.13 (1.01, 1.27), p < 0.05	Good
Ramos et al. (2021), (binary),^99^ PW = 43	Computer use (Internet)	Impairment Screen (MIS) test, Spanish version of Short Portable Mental State Questionnaire (SPMSQ), and Semantic Verbal Fluency (SVF) test	Gender, age, history of dementia, subjective memory complaint, educational level, marital status, feelings of depression and daily internet usage	OR = 0.56 (0.33, 0.95), p < 0.05	Good
Sha et al. (2022), (h/day),^84^ PW = 20	Reading	Chinese version of MMSE	Age, gender, ethnic group, current residence area, co-residence status, current marital status, years of education and occupation before 60 years, diabetes, heart disease, cerebrovascular disease, bronchitis, emphysema, asthma, and pneumonia (BEPA), and pulmonary tuberculosis	HR = 1.24 (1.00, 1.54)	Good
Sha et al. (2022), (h/day),^84^ PW = 20	Playing games and puzzles	Chinese version of MMSE	Age, gender, ethnic group, current residence area, co-residence status, current marital status, years of education and occupation before 60 years, diabetes, heart disease, cerebrovascular disease, bronchitis, emphysema, asthma, and pneumonia (BEPA), and pulmonary tuberculosis	mahjong or other card games HR = 1.23 (1.08, 1.39)	Good
Wang et al. (2006), (frequency),^89^ PW = 55	Watching television	Chinese version of MMSE	Age, sex, education, occupation, medical conditions, smoking, drinking, depressive symptoms baseline MMSE, ADL scores, participation in other activities	**Baseline** Significant difference in television viewing time between those who did not (18.84 ± 10.82) and those who did develop cognitive impairment (37.96 ± 11.96), p < 0.001 **Longitudinal** HR = 1.07 (1.06, 1.08)	Good
Wang et al. (2006), (frequency),^89^ PW = 55	Playing games and puzzles	Chinese version of MMSE	Age, sex, education, occupation, medical conditions, smoking, drinking, depressive symptoms, baseline MMSE, ADL scores, participation in other activities	**Baseline** Significant difference in time playing board games between those who did not (7.48 ± 7.61) and those who did develop cognitive impairment (2.45 ± 3.16), p < 0.001 **Longitudinal** HR = 0.92 (0.89, 0.94)	Good
Wang et al. (2006), (frequency),^89^ PW = 55	Reading	Chinese version of MMSE	Age, sex, education, occupation, medical conditions, smoking, drinking, depressive symptoms, baseline MMSE, ADL scores, participation in other activities	**Baseline** Significant difference in time reading between those who did not (1.53 ± 2.75) and those who did develop cognitive impairment (0.39 ± 1.14), p < 0.001 **Longitudinal** HR = 0.81 (0.74, 0.87)	Good
Wei et al. (2023), (frequency),^90^ PW = 40	Watching television	MMSE	Age, gender, education, rural/urban residence, region, marital status dummy variables, and number of chronic diseases	OR (95% CI) = 0.70 (0.62, 0.79)	Good
Wei et al. (2023), (frequency),^90^ PW = 40	Playing games and puzzles	MMSE	Age, gender, education, rural/urban residence, region, marital status dummy variables, and number of chronic diseases	OR (95% CI) = 0.73 (0.64, 0.84)	Good
Zhao et al. (2015), (frequency),^101^ PW = 48	Watching television, reading	MoCA	Gender, educational level, current smoker, alcohol consumption, smoking history, suffering from chronic diseases (including hypertension, diabetes, coronary heart disease and cerebrovascular disease	Participants with longer time watching TV (OR = 0.763 (0.628, 0.928), p = 0.007) and reading (OR = 0.540 (0.379, 0.769), p = 0.001) significantly decreased the likelihood of having MCI compared with the control group	Strong

ADL: activities of daily living; *APOE*: Apolipoprotein E; CDR: Clinical Dementia Rating; CI: confidence interval; CSI-D: Community Screening Instrument for Dementia; HR: hazard ratio; MCI: mild cognitive impairment; MMSE, Mini-Mental State Examination; MoCA: Montreal Cognitive Assessment; OR: odds ratio

*All sedentary activities are measured by self-report.

### Quality assessment

One reviewer independently evaluated the quality of each study using the QualSyst checklist developed by Maddocks et al.^29^ with a second author cross-checking the accuracy of the quality assessment for 10% of the included studies. The QualSyst checklist was chosen because it is applicable to both observational and experimental studies, making it a suitable tool for assessing the diverse study designs included in this review. Each study was assessed on 14 criteria, with scores ranging from 0 (no) to 1 (partial) and 2 (yes). The total score for each study was then normalized by dividing it by the highest possible score. Any discrepancies between reviewers were resolved through discussion to ensure consensus. A QualSyst score of 80% or higher indicated strong quality, scores between 60% and 79% were deemed good quality, scores between 50% and 59% were rated as adequate quality, and scores of 50% or lower were classified as poor quality.

### Data synthesis analysis

**Figure 1.**
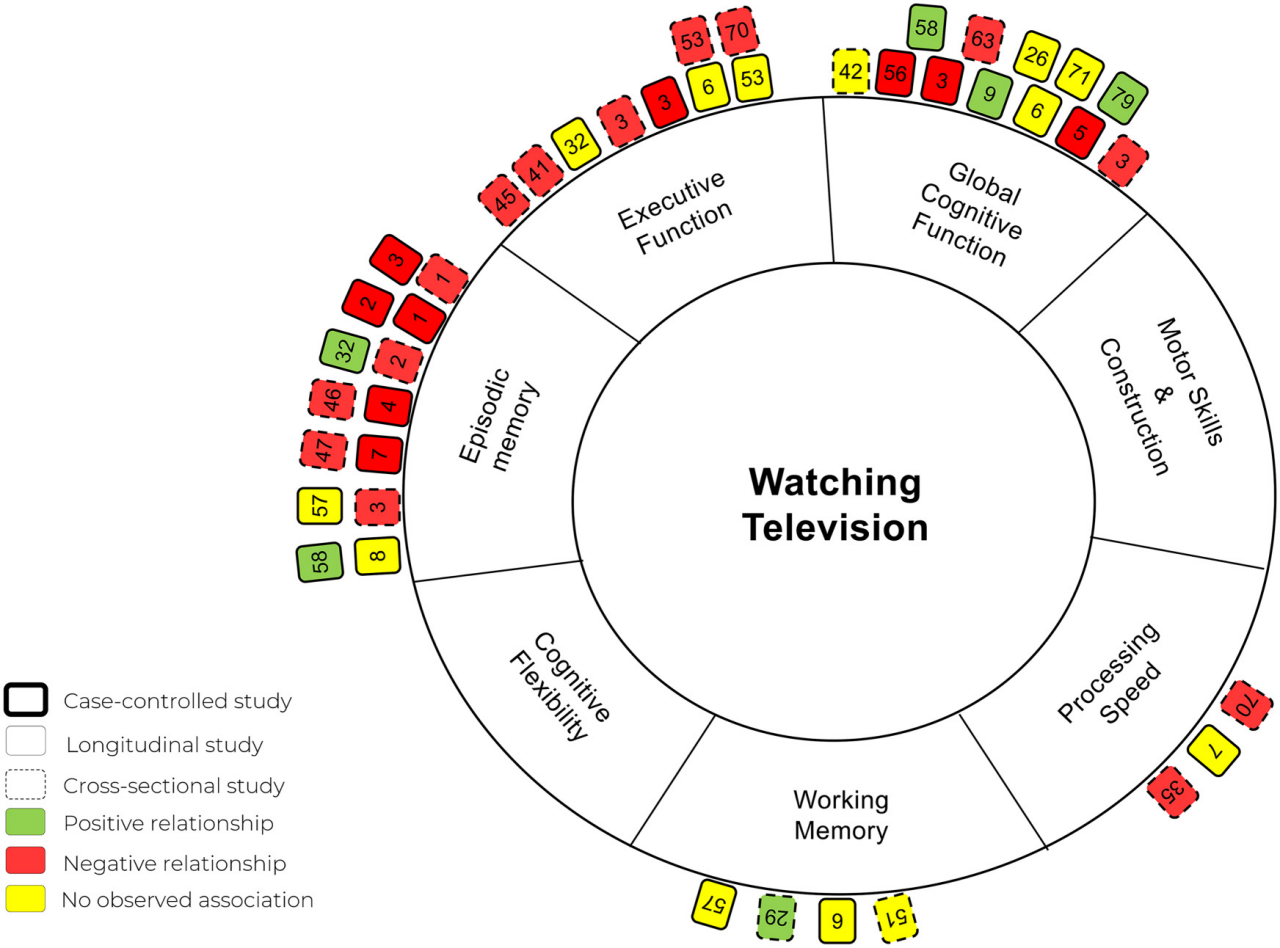
Pinwheel of all studies reporting association between watching television with seven cognitive domains.

**Figure 2.**
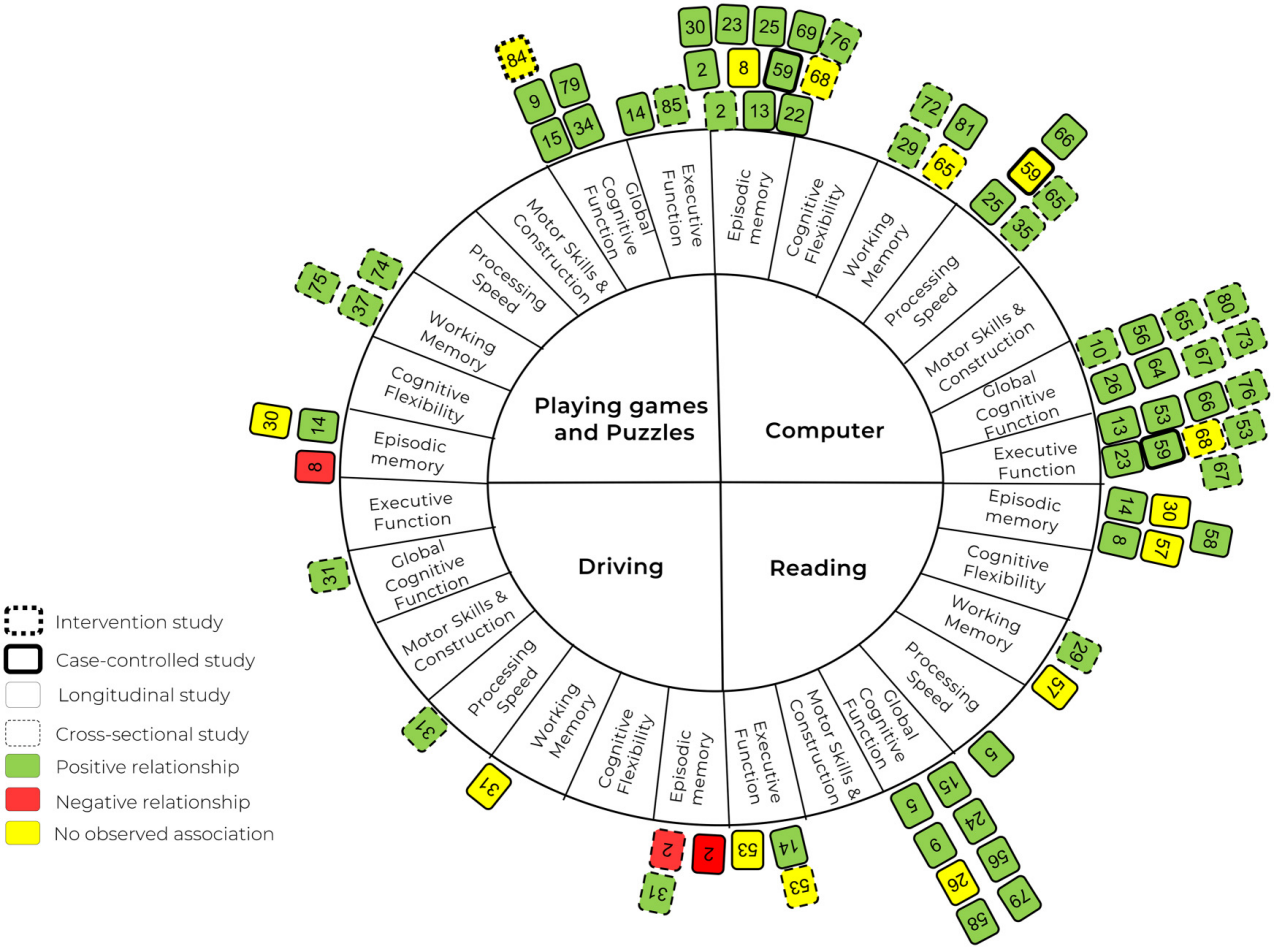
Pinwheel of all studies reporting association between Reading, computer use, playing games and puzzles, and driving with seven cognitive domains.

**Figure 3.**
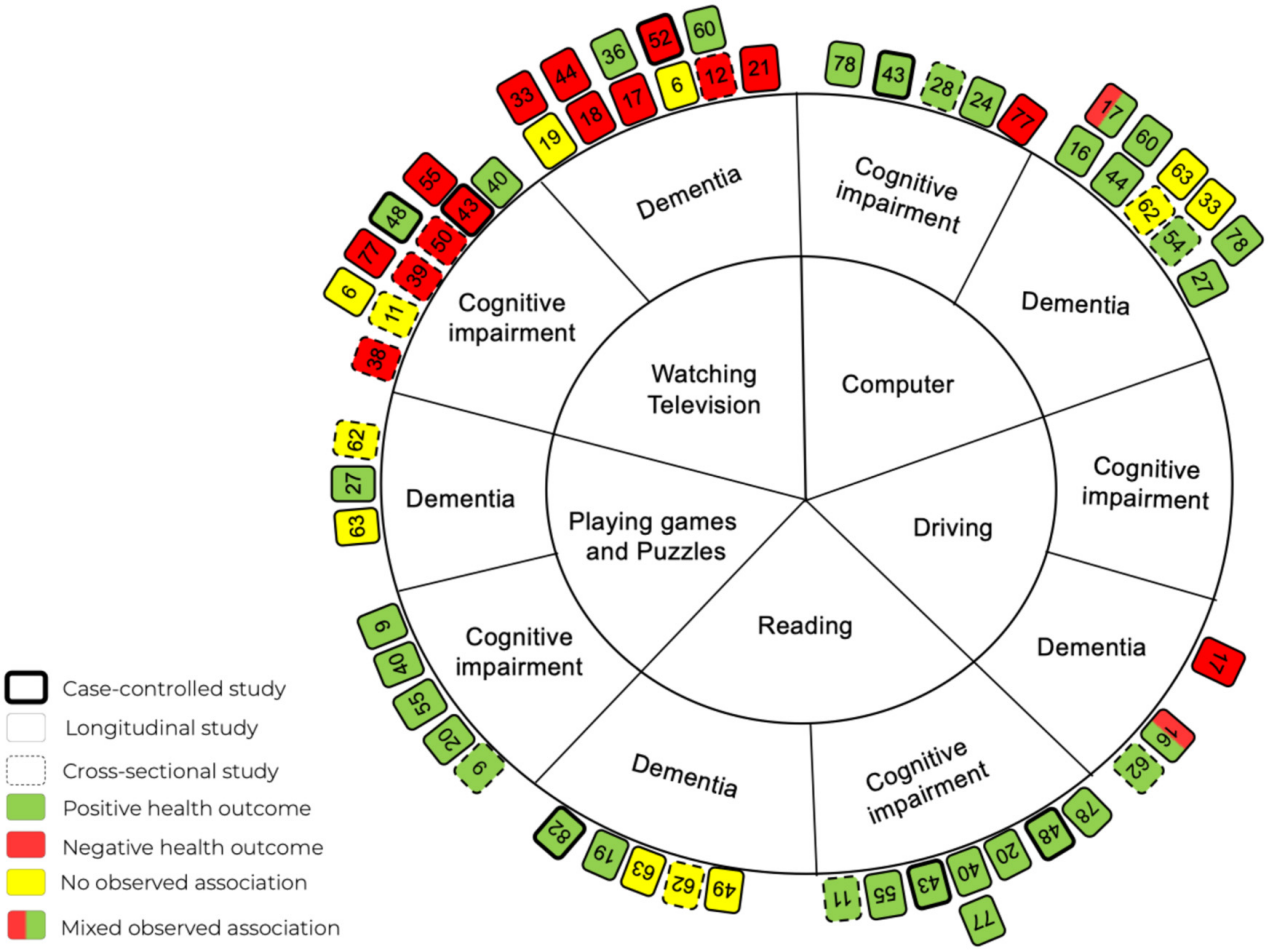
Pinwheel of all studies reporting association between individual sedentary activities with dementia and cognitive impairment.

All relevant studies reporting on individual sedentary activities and cognitive functioning were synthesized into three pinwheels based on their sedentary activity types and cognition types. Pinwheels were used as a visual representation to effectively organize and present the data, allowing for a clearer comparison of findings across different study designs and cognitive domains. Additionally, this approach facilitated the identification of research gaps by highlighting areas where evidence is limited or inconsistent. The first pinwheel represents passive sedentary activities, such as watching television (see Figure 1), and the second one representing active sedentary activities, including reading, games and puzzles, driving, and using computer (see Figure 2). Within the first two pinwheels, studies were further categorized based on their research design (cross-sectional, longitudinal studies, and case-controlled studies). Positive and negative effects were also highlighted on the pinwheels to indicate the direction of the relationship. A positive effect on the pinwheel indicates that a particular sedentary activity is associated with improvements in a cognitive domain (e.g., more reading increasing episodic memory), while a negative effect reflects a decline in cognitive function associated with that activity (e.g., more television viewing decreasing attention). Additionally, we also synthesized studies exploring the relationship between different sedentary activities and cognitive disorders in pinwheel 3, including dementia and cognitive impairment (see Figure 3). In this cognitive disorder-specific pinwheel, a positive health outcome indicates that engaging in more of a particular sedentary activity is associated with a reduced risk of dementia or improved cognitive health. Cognitive functioning was systematically categorized into seven distinct cognitive domains, allowing for a nuanced examination of how different sedentary activities influence specific cognitive functions. Moreover, we used different cognitive functions as outcome variables to examine whether different sedentary activities have different effects on a particular cognitive domain, highlighting which sedentary activities are associated with improvements or declines in each specific cognitive function. Finally, in the current systematic review, a meta-analysis was deemed inappropriate due to the heterogeneity of the associations explored, e.g., different sedentary activities and different domains of cognitive function. As meta-analyses generally require a minimum of five studies addressing the same specific research question to ensure sufficient statistical power and comparability of data, with 10 or more studies being the ideal threshold,^30^ a meta-analysis was not feasible.

## Results

### Search results and study characteristics

**Figure 4.**
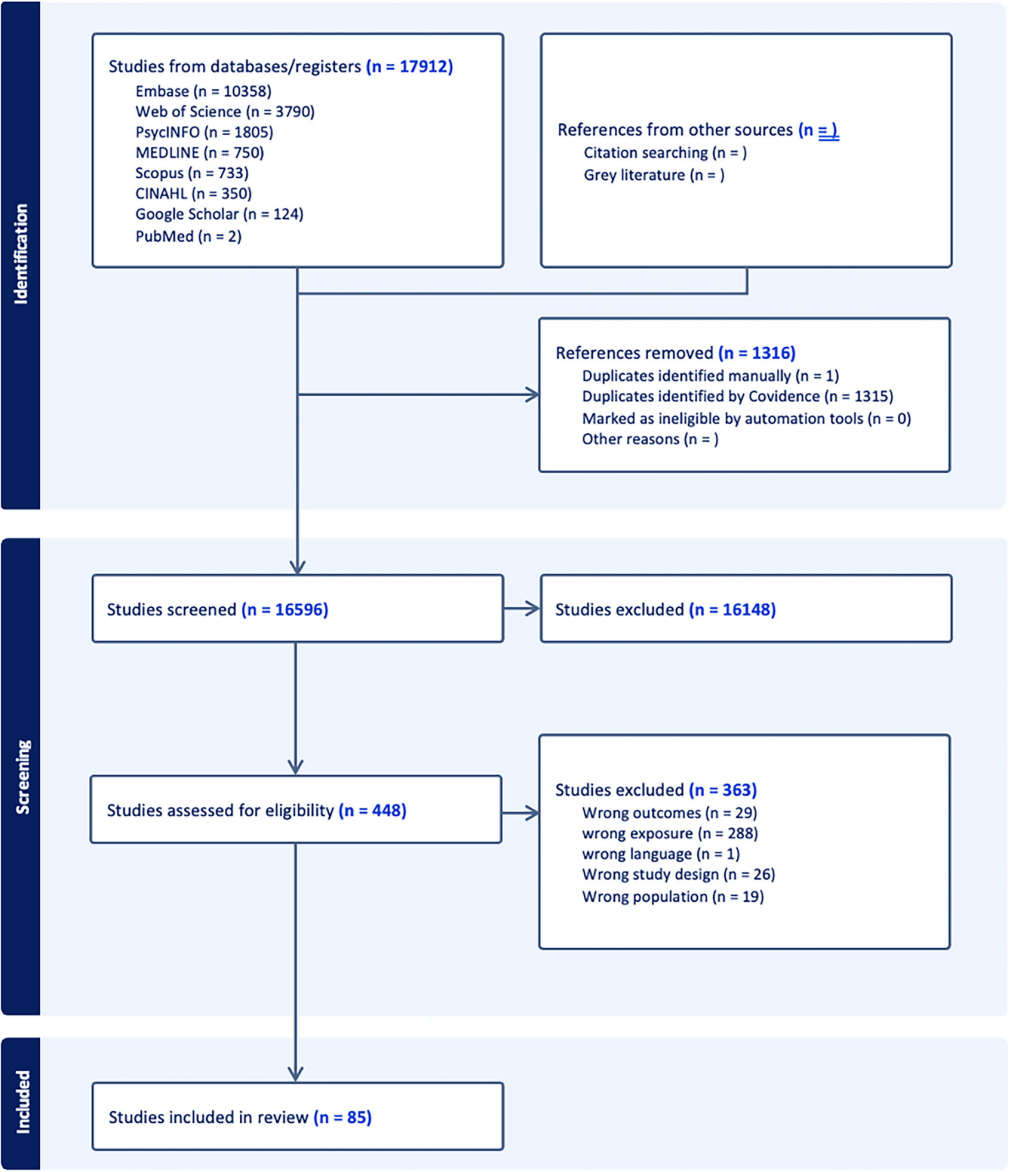
PRISMA flow diagram of the search and screening process in review of individual sedentary activities with cognitive function.

The initial electronic database search identified a total of 17912 papers. After removing 1316 duplicates, 16596 papers remained for title and abstract screening. During this stage, 16,148 studies were excluded, leaving 448 studies for full-text review. Following the full-text review, 363 papers were excluded for reasons like wrong exposure, wrong outcome, or wrong population, resulting in 85 studies that met the inclusion criteria (see Figure 4 for the PRISMA flow diagram). Among the 85 studies, 30 were cross-sectional studies,^12,31–59^ 44 were longitudinal studies,^12,16,17,25,39,58,60–97^ five employed a case-controlled design,^98–102^ one study was an intervention study,^103^ and five studies included both cross-sectional and longitudinal study data,^10,24,55,104,105^ (see Table 1). Overall, 49 studies included longitudinal analyses, exceeding the number of cross-sectional studies (n = 35), and thereby constituting the predominant study design. The length of follow-up for longitudinal studies ranges from two^104^ to 25 years.^68^ The publication years of these studies ranged from 2005^98^ to 2024, with 10 studies published in 2024.^25,35,37,48,56,60,72,73,94,106^ Among the 85 studies reviewed, several cohorts were utilized multiple times, including the UK Biobank cohort (n = 12),^31,51,56,72,83,86,87,92,93,95,97,105^ the Chinese Longitudinal Healthy Longevity Study (CLHLS) cohort (n = 6),^78,79,84,90,96,106^ the Survey of Health, Ageing and Retirement in Europe (SHARE) cohort (n = 3),^61,65,71^ the English Longitudinal Study of Ageing (ELSA) cohort (n = 3),^17,66,104^ the National Health and Aging Trends Study (NHATS) cohort (n = 3),^25,60,62^ the Health and Retirement Study (HRS) cohort (n = 2),^80,85^ the German Ageing Survey (n = 2),^80,81^ and the Pentoxifylline to Protect the Preterm Brain (PROTECT) cohort (n = 2).^34,107^

### Study population

The total sample size across the 85 studies was 1,575,657 middle-aged and older adults. Where studies were from the same cohort, e.g., the UK Biobank, the largest sample size was included in the total sample size, rather than the participant number from each study from that cohort. Individual study sample sizes ranged from 29^45^ to 851,307^64^ participants. The mean ages of participants in the included studies ranged from 36.9^45^ to 100^71^ years old, with the average percentage of female participants being 55.84, ranging from 14.6%^100^ to 100%.^64^ The majority of the studies were conducted in the United Kingdom (n = 20),^17,34,38,60,51,56,64,66,72,83,86,87,92–95^^,104,105,107,112^ the United States (n = 18),^16,25,32,33,37,43,48,52,60,62,67,68,74,80,85,98,100,113^ and China (n = 14),^53,55,59,79,42,78,84,89,90,96,100,101,106,109^ with other countries represented, including Japan (n = 7),^49,46,54,76,82,103,108^ Australia (n = 2),^47,91^ Germany (n = 2),^80,81^ Ireland (n = 2),^10,77^ the Netherlands (n = 1),^88^ Mexico (n = 1),^35^ Singapore (n = 1),^50^ South Korea (n = 2),^44,45^ Canada (n = 2),^36,41^ France (n = 2),^24,58^ Brazil (n = 1),^12^ Greece (n = 1),^110^ Iran (n = 1),^73^ Spain (n = 1),^99^ Switzerland (n = 1),^102^ and Thailand (n = 1).^57^ Four studies were conducted across multiple European countries.^39,61,65,71^

### Exposure (individual sedentary activities)

Tables 2–8 provide a detailed summary of the type of individual sedentary activities examined in the included studies. Five primary individual sedentary activities were identified: watching television, computer use, playing card games, reading, and driving. Among these, only watching television was categorized as a passive sedentary activity, due to its low cognitive engagement compared to the other activities, which involve more active mental participation.^22^ A total of 43 studies investigated the association between watching television and cognitive function, including both dementia and cognitive impairment.^10,16,17,24,25,35,36,39,41–44^^,47,49,50,52,56,59,63,66,68,73,85,87,89–91,94,95,97–99,12,77,82,83,95,104,105,108–110,114^ Twenty-five studies examined the impact of reading on cognitive function^16,24,35,42,49,54,56,61,63,64,66,71,73,74,76,79,82,84,85,89,99,90,102,109,115^. Thirty-five studies explored computer use.^10,24,31,33,35,46–51,53,56–58^^,63,65–70,70–72,74–76,80,81,83,85,87,88,91,94–96,99,100,102,110^ Seventeen studies assessed games and puzzles (including but not limited to mahjong, poker and puzzles), ^34,38,54,55,61,71,78,79,84,85,89,90,92,103,106,107,111^and six studies evaluated the impact of driving on cognitive function.^48,54,56,86,95,105^ Additionally, 29 studies investigated more than one sedentary activity.^16,24,35,44,49,51,54,56,63,71,73,74,82–86^^,88–92,94,95,99,101,105,109^ All sedentary activities across the included studies were assessed through self-reports or self-administered questionnaire, with the exception of one study that used monitoring software to assess computer use time.^32^

### Outcome (cognitive function)

Tables 2–8 present an overview of the various cognitive domains assessed in the included studies and summarize the tools used to measure cognitive function. Eleven studies assessed working memory,^12,34,35,38,45,48,58,70,73,79,107^ nine studies focused on processing speed,^16,17,48,51,58,68,76,81,100^ global cognition was evaluated in 22 studies,^10,12,16,18,31,33,42,48,53,55,56,58,66,75,78,79,81,88,103,106,109,110^ 16 studies examined executive function,^10,12,24,25,32,36,60,33,40,59,62,67,68,76,100^ and 22 studies assessed episodic memory.^10,17,25,31,41,43,48^^60–62^^,65,67,71,73,77,81,85,96,100,104,105,109^ Additionally, 20 studies evaluating more than one cognitive domain.^10,12,16,17,25,32,33,48,58,60–62^^,67,68,73,76,79,80,100,109^ Episodic memory was measured using 14 different tools, with the most common being immediate and delayed recall tests (n = 10). Global cognition was assessed using 13 different measures, with the Mini-Mental State Examination (MMSE) being the most frequently used (n = 14). Working memory was assessed with eight different tools, executive function with 13, and processing speed with seven. Table 4 summarized the association between different individual sedentary activities with impaired cognition. Twenty-one studies focused on dementia as a cognitive outcome,^12,54,56,57,63,64,72,74,82,83,86,87,91–95^^,97,98,102,108^ and 11 studies assessed cognitive impairment.^39,44,46,49,50,75,80,84,89,90,101^ Hospital inpatient records were the most common tool for measuring dementia (n = 11), while the MMSE was the most frequently used tool for assessing cognitive impairment (n = 8).

### Association of passive sedentary activities with cognitive function

The studies investigating the association between passive sedentary activities (television viewing) and cognitive function are summarized in Table 2 and illustrated in Figure 1. Four studies explored the relationship between television viewing and working memory,^12,35,73,98^ three studies examined its impact on processing speed,^17,51,68^ 11 studies assessed global cognitive function,^10,12,16,18,55,66,78,81,88,109,110^ seven investigated executive function,^10,12,17,25,36,59,68^ and 11 studies focused on episodic memory.^10,17,25,41,43,73,77,85,104,105,109^ Overall, most studies reported a negative association, indicating that increased time spent watching television was linked to poorer cognitive function. However, five studies reported a positive association,^25,35,55,78,109^ suggesting that more television viewing was associated with better cognitive outcomes. The association between television viewing and global cognition yielded mixed findings. Among the 11 studies reviewed, four reported a negative effect,^10,16,56,66^ three identified a positive effect,^55,78,109^ and the remaining four found no significant effect on global cognition.^12,47,88,110^ Television viewing demonstrated a strong association with decreased episodic memory in the 11 studies reviewed. Among these, two studies reported positive effects, two found no significant effect, and the remainder consistently reported negative effects on episodic memory. A total of 11 cross-sectional studies reported a negative relationship between television viewing and cognitive function,^10,24,36,41,43,51,59,68,97,104,105^ while seven longitudinal studies similarly identified a negative association.^10,16,17,66,77,104,105^ In contrast, only one cross-sectional study reported a positive relationship,^35^ compared to four longitudinal studies that observed a positive association^25,55,78,109^

### Association of active sedentary activities with cognitive function

The studies examining the association between active sedentary activities (computer use, reading, playing card games, and driving) and cognitive function are summarized in Tables 3–6 and illustrated in Figure 2. Regarding reading (Table 3), five studies explored its relationship with episodic memory,^36,71,73,85,109^ two studies assessed its link with working memory,^35,73^ one study investigated its effect on processing speed,^16^ eight studies evaluated global cognitive function,^55,66,79,80,12,78,88,109^ and two studies assessed executive function.^24,61^ Eleven studies investigated the relationship between computer use and episodic memory,^32,60,62,65,67,71,81,85,96,100,105^ four study assessed the link between computer use and working memory,^35,45,70,76^ five studies examined its association with processing speed,^58,76,81,93,100^ eight studies explored executive function,^24,32,33,60,62,67,76,100^ and eight studies investigated global cognitive function^31,33,42,53,58,80,88,89^ (see Table 4). Meanwhile, three studies examined the impact of games and puzzles on episodic memory,^61,71,85^ three examined working memory,^34,38,107^ five examine global cognition,^55,78,79,103,106^ and two examined executive function^40,61^ (see Table 5). For driving (Table 6), one study evaluated its association with global cognitive function,^48^ processing speed,^48^ and working memory.^48^ Overall, most studies reported a positive association, indicating that increased engagement in these active sedentary activities was linked to better cognitive function. However, two studies identified a negative association with games and puzzles (8) and driving (2), and eight studies reported no significant effect.^16,55,75,79,78,88,89,109^ Computer use showed a particularly strong positive relationship with global cognition, executive function, and episodic memory. Among the 27 studies investigating computer use across these three cognitive domains, only four showed no effect,^32,58,85,100^ with the remaining studies all reporting positive effects. Similarly, reading was strongly associated with global cognition, as seven out of eight studies identified a positive association, with only one study reporting no effect.^88^ There was no significant variation in the findings across the different study designs.

### Association of sedentary activities with impaired cognitive function

The studies evaluating the association between individual sedentary activities and impaired cognitive function is summarized in Tables 7 and 8 and illustrated in Figure 3. Three studies assessed the relationship between playing card games and dementia,^54,81,91^ Four studies examined driving and dementia,^54,56,63,74^ seven studies evaluated reading and dementia,^54,56,63,74,81,82,102^ 12 studies explored the link between television viewing and dementia.^12,62,63,82,83,87,91,93–95^^,97,98^ Regarding cognitive impairment, seven studies evaluated television viewing,^39,44,49,50,89,90,99^ six studies assessed reading,^49,84,89,90,99,101^ four studies examined playing card games,^84,89,90,101^ and three studies assessed computer use.^46,75,99^ Overall, most studies reported positive cognitive health outcomes associated with reading, playing card games, and computer use, while an increased amount of television viewing was generally associated with worse cognitive impairment. One study reported that very low (0 h/day) and high (≥2 h/day) levels of television viewing were associated with increased dementia risk compared to moderate viewing levels (>0 to <2 h/day).^95^ Another non-linear relationship was observed for driving whereby individuals who did not drive at all had a significantly higher risk of dementia (HR = 1.41, 95% CI: 1.32–1.51, p < 0.001) compared to those who drove moderately (>0 to <2 h/day).^95^ However, prolonged driving (≥2 h/day) was also associated with an increased risk (HR = 1.33, 95% CI: 1.22–1.44, p < 0.001).^95^ Reading demonstrated a strong association with positive health outcomes, as among the 13 studies examining its relationship with dementia and cognitive impairment, only three reported no effect,^54,56,64^ while the remaining studies found positive outcomes. Similarly, playing card games showed a strong relationship with improved dementia and cognitive impairment, with only two of the seven studies reporting no effect.^54,56^ In this review, cross-sectional studies typically compared sedentary time between individuals with and without dementia, assessing differences in their current cognitive state, while longitudinal studies examined how sedentary activity earlier in life influences cognitive function over time.

Results from the UK Biobank studies showed different directions and strengths of associations in the relationships of each sedentary activity and cognition/dementia. This is demonstrated in the findings of studies examining the relationship of computer use with dementia. For example, Jia et al. (2024) found that frequent computer use was linked to a 19% lower risk of dementia (HR = 0.81, 95% CI: 0.69–0.94);^72^ Xu et al. (2024) found no significant association between computer use and dementia risk (HR = 0.86, 95% CI: 0.66–1.12, p = 0.402);^94^ and, results for Yang et al. (2023) found that moderate computer use (>0–<2 h/day) appears to be beneficial for dementia risk, whereas both no use (0 h/day) and excessive use (≥2 h/day) are associated with higher dementia risk.^114^

### Quality assessment score

The overall average quality score across the studies was 75.2%, with individual study scores ranging from 45.8% to 91.7% (as detailed in Supplemental Table 5). A total of 32 studies were classified as meeting the criteria for “good quality”,^33,35,37,39–41^^,46,47,51,53,55–58,60,63–66,68,76,78,82–84,89,90,93,97,98,100,104^ 38 studies were identified as “strong quality”,^10,12,16,17,24,25,31,32,43,44,48,49,52,54,59,62,67,72–75^^,77,80,81,85–88,91,94–96,101,105,106,108–110^ indicating a high level of methodological rigor, seven studies were identified as “adequate quality”,^32,34,38,45,50,102,107^ and two studies were categorized as “poor quality”.^42,103^ Associations of sedentary activities with cognitive outcomes from studies categorized as of adequate quality^32,34,38,45,50,102,107^ were in the same direction as the majority of other studies in those sections of the pinwheel.^32,86,90,100,101^

## Discussion

The purpose of this systematic review was to describe and synthesize existing evidence on the associations between different types of sedentary activities and cognitive outcomes. Specifically, this review aimed to identify how passive and active sedentary activities were differentially associated with key cognitive domains (e.g., episodic memory, executive function, and working memory) to elucidate the cognitive domains that are most susceptible to these activities. In addition, we explored the role of sedentary activities in cognitive decline or cognitive protection in older adults. Overall, the results of this systematic review highlight significant associations between different sedentary activities and cognitive function. Across study designs, the passive sedentary activities (e.g., watching television) were most consistently associated with negative cognitive outcomes, including increased risk of dementia and cognitive impairment, as evidenced by multiple studies with predominantly negative associations. In contrast, active sedentary activities (e.g., reading, playing card games, and using a computer) showed overwhelmingly positive associations with cognitive health, enhancing cognitive functions such as executive function, situational memory, and working memory. Importantly, longitudinal studies, which provide stronger evidence for causality, generally supported these patterns. Effect sizes typically were small in cross-sectional studies but small to medium for the longitudinal studies, underscoring the greater strength of the latter.

There was some preliminary evidence to suggest that the relationship between the sedentary activities of both driving and TV viewing with cognitive outcomes, particularly incident dementia, may not be linear.^95^ Specifically, one large prospective cohort study reported U-shaped associations for TV-watching and driving time, and a reversed J-shaped association for nonoccupational computer use, with the lowest dementia risk observed at >0–<2 h/day for each behavior. These findings indicate that both inactivity (complete absence of an activity) and excessive engagement may increase dementia risk, highlighting a potential U-shaped or threshold effects.^95^ This non-linearity underscores the need for further longitudinal research to determine whether specific sedentary activities have an optimal engagement level that minimizes cognitive risks and whether these effects vary across cognitive domains.

### Passive sedentary activities

Our findings consistently show significant negative associations between passive sedentary behaviors (e.g., television viewing) and a variety of cognitive domains. Cross-sectional studies frequently identified negative associations, particularly with episodic memory, but these designs are limited by the inability to establish temporal direction. Importantly, longitudinal studies provide stronger support for these associations, with several showing that higher television viewing predicts greater risk of dementia and cognitive decline over time. The most significant association observed in our analyses was in episodic memory, with 10 of the 14 studies reporting a negative association between higher TV viewing time and situational memory performance. Taken together, the longitudinal evidence provides the most compelling case that prolonged television viewing may harm cognition, though further research is needed to clarify the role of program content, viewing patterns, and contextual factors.

One potential mechanism for this decline is that TV watching is often associated with continuous TV watching behavior (e.g., binge watching), and this pattern of sedentary behavior negatively impacts memory consolidation.^86^ Memory consolidation is the process of converting short-term memories into long-term memories.^86^ This process is designed to transfer memories from the hippocampus (the part of the brain associated with short-term gains and instincts) to the cerebral cortex (the part of the brain associated with rational/deeper thinking) and to build long-term memories by increasing the complexity, distribution, and connectivity of the cortex relative to the rest of the brain.^86^ Continuous television viewing interferes with this process because it does not provide enough time for the formation and transfer of clear memories. This is further explained by the theory of temporal uniqueness, which suggests that memory recall can be enhanced when there is sufficient spacing between events.^116^ However, it is unclear from our findings whether watching TV interrupts the consolidation of short-term memory from other periods of the day by preventing the successful transfer of memories to the cerebral cortex. This lack of “downtime” may disrupt the consolidation of neutral memories, preventing them from performing properly.^117^ Thus, the same cognitive principles that apply to episodic memory formation in TV viewing can be generalized to other memory-related tasks. Whether individuals are engaging in educational activities, professional work, or personal learning, incorporating breaks, and allowing time for reflection between episodes is essential for effective memory consolidation. This reinforces the importance of spaced repetition techniques and underscores how passive, continuous exposure to information—without adequate intervals for reflection—can diminish long-term memory performance across different domains.

The negative effects of television viewing on executive functioning stem from the lack of cognitive engagement required for passive activities. Executive functioning refers to cognitive processes that rely on higher-order cognitive processing, such as planning, decision-making, and task-switching, which require frequent activation of the prefrontal cortex, the area of the brain responsible for higher-order thinking.^118,119^ In contrast, during television viewing, especially prolonged continuous television viewing, cognitive processes that typically rely on active engagement are underutilized, and in the absence of such cognitively engaged stimuli, neural pathways associated with executive functioning are weakened over time, leading to atrophy.^87^ Simply put, just as a muscle weakens without exercise, the areas of the brain responsible for complex thinking deteriorate without active participation. Executive function requires constant use of these cognitive “muscles” through activities that require planning, problem solving, and quick thinking. Watching television provides little cognitive stimulation, leading to a decline in these functions, especially in older adults who are already facing natural cognitive aging.

While our review confirms much of the existing literature, it also highlights research gaps that warrant further exploration. Specifically, contextual factors, such as whether television programs are educational and whether individuals take regular breaks during viewing, have not been adequately studied. In addition to the lack of studies on television content and breaks during viewing, other contextual factors such as snacking may also influence cognitive outcomes. Snacking during television viewing could contribute to mindless eating, potentially affecting overall health and cognitive function indirectly through dietary patterns and weight gain, both of which are linked to cognitive decline.^120,121^ Addressing these factors may provide more comprehensive insights into how passive sedentary behavior affects cognitive function.

### Active sedentary activities

Our data indicate that most studies demonstrate a positive correlation between active sedentary activities and cognitive functioning, which contrasts with the negative associations often observed in passive activities such as television viewing. Here again, longitudinal studies provide the strongest evidence: reading, computer use, and puzzle play were associated with better executive function, episodic memory, and overall cognition over time, even after accounting for baseline performance. Cross-sectional and case-control designs supported these benefits, reinforcing the robustness of these findings. The cognitive engagement required for these active activities appears to play a critical role in maintaining or even improving cognitive performance across multiple domains, including executive function, situational memory, and overall cognitive function. Reading is associated with positive outcomes in almost all cognitive domains. Studies have shown that reading is positively correlated with situational memory, executive functioning, and overall cognitive functioning, with no negative correlations observed.^122^ This is because reading typically requires active participation through comprehension, memory recall, and mental visualization, challenging the brain to process complex narratives and ideas.^122^

In addition, reading involves multiple regions of the brain, including the temporal lobe, Broca's area, and the angular gyrus, all of which are involved in language comprehension, memory recall, and mental visualization.^123^ Reading also stimulates white matter pathways, enhancing the brain's ability to process information and form neural connections, which supports overall cognitive health and reduces the risk of cognitive decline.^123^ Research shows that reading rewires your brain, creating new neural networks and strengthening white matter in the corpus callosum, which enhances communication between the two brain hemispheres.^124^ These pathways improve overall cognitive function, which in turn improves your ability to actively recall information. The relationship between reading and reduced risk of cognitive decline may reflect the effects of education.^125^ Previous research on early educational achievement and cognitive functioning in later life is controversial. Specifically, some studies supporting the cognitive reserve (CR) hypothesis suggest that higher levels of education provide a buffer against age-related cognitive decline and dementia. The CR hypothesis posits that individuals with higher education develop more robust neural networks, which allow them to better compensate for brain changes associated with aging, thus delaying the onset of dementia or cognitive impairment.^126^ However, other research challenges this idea, suggesting while education is positively associated with cognitive performance at baseline, it does not appear to slow the rate of cognitive decline over time, meaning that education contributes to a higher starting point in cognitive abilities but does not alter the trajectory of cognitive aging.^127^ However, although our data support the cognitive benefits of reading, this review did not consider stratified analyses for educational level; therefore, future research should explore the relationship between education and cognitive function more thoroughly to understand this link more clearly.

Similarly, data on playing games and puzzles show mostly positive associations with cognitive functioning. This is largely because card games involve strategic thinking, problem solving, and often social interaction. These games require the brain to switch rapidly between different cognitive tasks. To win or play well in the game, players need to coordinate and work with a variety of abilities, such as attention, observation, alertness, memory, recall, calculation, language, and communication, which, when used repeatedly in games, can improve and maintain cognitive functioning in older adults.^19,128^ Interpersonal interactions and emotional stimulation during play can further support the reactivation of neural circuits, positively affecting decision-making and cognitive health.^129^ This dynamic mental stimulation may contribute to positive effects on executive function and memory, as these areas are closely linked to the brain's ability to adapt and multitask. In contrast to passive activities, playing card games provides sustained cognitive engagement that strengthens neural circuits related to decision-making, strategy development, and working memory. In addition, playing games and puzzles requires a certain amount of upper-limb physical activity in addition to rich emotional and environmental stimuli, which may increase cerebral blood flow, stimulate central nervous system excitation, improve brain tissue metabolism, and promote the establishment of brain neural networks, leading to adaptive changes in brain structure and function.^130–132^ Furthermore, playing games and puzzles helps to improve social support, reduce psychological stress, and protect stress-related neurons, all of which benefit cognitive function.^132,133^

The use of computers is another sedentary activity that is closely associated with cognitive benefits in the areas of executive function, overall cognitive function, and situational memory. Tasks performed on computers are often complex—whether for work, communication, or recreation—and typically require sustained attention, memory use and decision-making, all of which require cognitive abilities. Thus, this activity, although sedentary, can mobilize multiple cognitive processes simultaneously. Unlike passive activities, computer use encourages higher levels of cognitive engagement, such as problem solving and information processing, both of which contribute to better cognitive outcomes.^134^ In addition, the use of computers and the Internet may provide older adults with opportunities to step out of their comfort zones and engage in more alternative activities, which in turn may stimulate cognitive functioning and provide cognitive benefits.^135^ Computer use is also an interactive activity that poses special challenges for older adults, including increased psychomotor abilities when using a computer mouse, as well as sensory and cognitive abilities (e.g., speed of searching for icons), and problems with learning, memory, and executive functioning, all of which may be affected with age. Computer use may also have specific links to executive function. Neuroimaging has shown that older adults utilize prefrontal cortical areas during functional tasks and that middle aged and older adults with more computer experience have greater brain activation in frontal areas of the brain during Internet search tasks.^136^

The cognitive advantages of driving may be related to processing speed and working memory. Driving is a complex activity with multitasking, spatial awareness, decision making, and problem solving, all of which require executive functions of the brain.^137^ While driving, people must constantly process visual and auditory information, respond to environmental stimuli, and make split-second decisions that activate brain areas associated with attention, working memory, and cognitive flexibility. Longitudinal evidence provides mixed findings in this regard. One study reported that greater driving time was associated with small but significant improvements in visual-spatial and short-term numeric memory, suggesting that the cognitive demands of driving may help maintain cognitive flexibility and executive functioning.^105,114^ In contrast, two studies found that both no driving and higher levels of daily driving were linked to increased dementia risk, indicating a potentially non-linear relationship.^86^ These inconsistent results highlight the complexity of driving as a cognitively stimulating activity, where benefits may depend on the level of engagement and individual context. Although fewer studies have focused on driving compared to other activities such as reading or computer use, the available evidence suggests that the cognitive demands of driving may help maintain cognitive flexibility and executive functioning, which are crucial for cognitive health in aging individuals. However, it is also important to consider the possibility of reverse causation in this relationship. It could be that individuals who continue to drive as they age are doing so because they already possess better cognitive function, rather than driving itself being the cause of maintained cognition. In other words, people with higher cognitive abilities may be more capable of continuing to drive, which makes it difficult to determine whether driving actively preserves cognitive function or simply reflects the individual's pre-existing cognitive status. Further research is needed to disentangle these effects and clarify whether driving directly supports cognitive health or is merely a marker of better cognition​.

Differences in study results, particularly those from the UK Biobank studies, suggest that variations in sample characteristics, follow-up duration, and study design may influence findings. For example, some UK biobank studies included younger participants at baseline, whereas other studies primarily focused on older populations already at risk for cognitive decline. This age difference is critical, as cognitive impairment and dementia typically develop over long periods. Younger participants may not yet show cognitive deficits**,** potentially leading to weaker or non-significant associations compared to studies with older adults who are more susceptible to cognitive decline. Longer follow-up periods in Biobank studies allow for a more comprehensive assessment of long-term cognitive trajectories. In contrast, some UK Biobank studies with shorter follow-up periods may only capture intermediate cognitive changes, missing the gradual impact of sedentary behaviors on cognition. As a result, studies with longer follow-ups are more likely to detect delayed effects of sedentary behaviors on cognitive decline, while shorter studies may report weaker associations. Meanwhile, UK Biobank studies typically involve large population-based cohorts, whereas some other studies included smaller, more targeted samples. The larger sample sizes in Biobank studies provide greater statistical power, but the broad population may dilute the effects seen in studies focusing on high-risk groups (e.g., older adults or those with existing cognitive impairment).

### Strengths and limitations of the review

This systematic evaluation has several significant strengths. First, it distinguishes between passive and active sedentary activities and examines in detail how individual sedentary activities are differentially associated with cognitive functioning in older adults. Second, the inclusion of different study designs—including cross-sectional, longitudinal, intervention, and case-control studies—increases the robustness and breadth of the findings. Longitudinal, case-control, and cross-sectional studies yielded consistent findings regarding active sedentary activities, indicating similar effects on cognition. However, the findings on the relationship of TV with cognitive function were more mixed across study types with more longitudinal studies demonstrating positive associations. Thirdly, several of the included longitudinal studies controlled for baseline cognitive function. This methodological feature helps reduce the risk of reverse causation, where early cognitive decline may lead individuals to disengage from cognitively stimulating activities and increase their engagement in passive behaviors such as television viewing. By adjusting for pre-existing cognitive status, these longitudinal designs provide stronger evidence for a potential causal relationship between sedentary activities and cognitive outcomes, thereby offering greater confidence in the observed associations compared to cross-sectional studies, where temporal ordering cannot be established. Importantly, the length of follow-up across these studies ranged from two to 25 years, allowing for both short-term and long-term trajectories of cognitive change to be examined. Another strength is the use of large population-based datasets from multiple countries and that we followed the PRISMA guideline. The use of the pinwheel also identified a lack of evidence that no studies have examined the impact of sedentary activities on motor skills and coordination or cognitive flexibility.

This systematic evaluation also has some concurrent limitations. While in this review, cognitive domains were assessed by several different measures, methods for assessing cognitive function in the included in the studies were all well validated which supports the robustness of our findings. Another limitation of this systematic review is the assessment of sedentary activities. Data were not available regarding posture, content of the activities, e.g., type of television program, and social interactions. A proposed taxonomy suggests also collecting data around choice and novelty which may provide further information on the mechanisms of the associations of sedentary activities with cognition.^138^ The current literature precluded conducting a meta-analysis which may provide further evidence to quantify the relationships described in this review. Future research should focus on conducting meta-analyses to strengthen the evidence base and quantify these relationships more robustly, provided that a sufficient number of appropriate studies are available. Moreover, another limitation is the uncertainty regarding whether the effects of individual sedentary activities were adequately adjusted for other concurrent sedentary activities. We therefore do not know whether any of the active sedentary activities may confer protective benefits against the impacts on cognition of watching television. Furthermore, the studies were limited in adjusting for other risk factors for dementia listed in the Lancet Commission paper. Future studies should ensure that such adjustments are made to better understand the distinct effects of each sedentary activity.

In addition, many of the included studies, although prospective, relied on only one or two measurement time points, which makes it difficult to conclude whether the observed associations are predictive of cognitive decline or instead reflect early indicators of preclinical impairment. Sedentary activities may act as more sensitive markers than cognitive assessment tools, and although this review highlighted reverse causation primarily in relation to driving, it is also reasonable to consider that reverse causation may influence other sedentary activities, such as TV viewing or computer use, where early cognitive decline could alter participation patterns.^139^ Taken together, while the long follow-up periods in some cohorts strengthen causal inference, the reliance on limited time points in others suggests that changes in behavior may sometimes be better interpreted as indicators of emerging impairment rather than causes of decline. Additionally, most of the studies in this review were from the UK (UK Million Women Study and UK Biobank cohort). Furthermore, all included studies were conducted in high-income countries, meaning that populations from low- and middle-income countries were not represented. This geographic limitation is important, as socioeconomic and cultural differences can influence both sedentary behavior patterns and cognitive health outcomes. The lack of studies from diverse regions restricts the applicability of the findings to global populations, highlighting the need for future research to explore these associations in underrepresented geographic areas and diverse socioeconomic contexts. The last limitation is that studies were only published in English.

### Practical implications

The broader public health implications of these findings suggest that existing guidelines should be updated to reflect the cognitive risks associated with sedentary activities. However, current public health campaigns tend to focus on the physical health effects of sedentary activities, such as cardiovascular disease, cognitive effects are less emphasized. For example, during the World Health Organization's 2020 Guidelines on Physical Activity and Sedentary Behavior development process, the Guideline Development Group (GDG) identified all-cause mortality and cardiovascular mortality as the most critical outcomes to be addressed.^140^ These were followed by other clinical outcomes, including falls, depression, cognition, and health-related quality of life. While cognition was included, it was not given the same priority as physical health outcomes, reflecting the broader tendency to focus on physical risks such as heart disease and mortality, rather than the cognitive consequences of sedentary activities.^140^ With the trend towards 24-h movement behavior guidelines, such as the Canadian 24-Hour Movement Guidelines for adults and older adults, it is important to carefully consider which activities constitute the sedentary time component of the day.^141^ Our findings suggest that it may not be beneficial to group television time and computer use together as “screen time,” given the differing associations with brain health. For instance, passive activities like television viewing are more strongly associated with cognitive decline, whereas active sedentary activities, such as computer use, may provide cognitive benefits. Therefore, future public health guidelines should place a greater emphasis on the cognitive health impacts of sedentary activities and distinguishing between passive and active sedentary activities. Additionally, given the growing trend of obsessive behaviors around television public health messages could emphasize the cognitive risks of prolonged time spent on television viewing and provide strategies to integrate more rest and cognitive engagement during these periods. For example, public health campaigns could recommend limiting daily television consumption and encouraging physical activity or cognitive activity during breaks, such as puzzles, social interactions, or brief exercise.

### Research gaps and future directions

This systematic review reveals several key research gaps and opportunities for future research. First, the need for experimental studies is clear, as most studies to date have been observational, which limits our understanding of the causal relationship between sedentary activities and cognitive decline. Experimental designs can more rigorously test how interventions mitigate the deleterious effects of chronic passive activities. Another key area for further exploration is the intersection of physical and cognitive health. Sedentary behaviors often coincide with a lack of physical activity, and it is critical to understand how different levels of physical activity can mitigate the cognitive risks associated with sedentary lifestyles. Research should explore whether increased physical activity can counteract the negative cognitive consequences associated with passive behaviors, potentially identifying protective factors that maintain neuroplasticity and cognitive reserve. In addition to sedentary activities, the role of cognitive training deserves further attention. We excluded more than 200 studies on cognitive training, and these interventions have shown great potential to enhance cognitive functioning, particularly in the areas of executive function, memory, and attention.^26–28^ Future research should investigate whether cognitive training can be effectively combined with recommendations to reduce passive sedentary activities, resulting in a multifaceted approach to protecting cognitive health in older adults. Such integrated interventions could provide a more comprehensive solution to slowing or mitigating age-related cognitive decline. Another notable gap in the current literature is the lack of consensus regarding the distinction between active and passive sedentary activities, which can obscure the unique effects each type may have on cognitive and health outcomes. To address this, future research could benefit from investigating the cognitive load of different sedentary activities and developing consensus using a Delphi study methodology, where a panel of experts systematically classifies sedentary activities into active and passive categories.

Additionally, the review calls for more research on content-specific sedentary behaviors, particularly in the context of television viewing. While existing studies did not differentiate program type, it is plausible that more intellectually stimulating content (e.g., documentaries or educational programs) may confer different cognitive effects compared to purely entertainment-focused content. Examining these distinctions, along with the role of social interactions during viewing, will provide a more detailed understanding of how different contexts of sedentary activities contribute to or mitigate cognitive risk. Another important research gap is the role of social media use. Although not examined in the studies included in this review, social media represents a rapidly growing sedentary activity that may differ cognitively from both passive television viewing and active computer use. Future research should investigate whether the interactive features of social media confer cognitive benefits or, conversely, contribute to cognitive overload and distraction. Future research should also include the opportunity to explore these associations in individuals residing in aged care facilities. This population typically experiences more comorbidities, increased sedentary time, poorer cognition, and decreased physical activity, making them an important group for studying the cognitive impacts of sedentary behaviors.^142^ Understanding these associations in such a vulnerable population could offer valuable insights into how sedentary behavior interventions might be tailored to improve cognitive outcomes and overall health in aged care settings. Future studies also need to examine the associations between sedentary activities and other cognitive domains, such as cognitive flexibility, which were not included in this review due to the lack of studies reporting outcomes in this area. Finally, only one of the included studies used a device to measure sedentary activities, the rest relied on self-reported data, which is prone to recall bias and inaccuracy. This is because older adults’ physical activity participation is often intermittent, sporadic, or unstructured, which makes recall extremely difficult, and older adults may inadvertently exaggerate their sedentary activities. Moreover, a previous review reported that studies that used devices to measure sedentary time showed a significant negative association with cognition, while studies relying on self-reporting found a positive association between sedentary time and cognitive function.^20^ Therefore, future research on the association between sedentary activities and cognitive function should use both objective and subjective measures whenever possible, although using device is very difficult and expensive.

### Conclusion

In conclusion, this systematic review comprehensively examined the differential impacts of passive and active sedentary activities on cognitive function in middle-aged and older adults. The findings indicate that passive sedentary activities (e.g., watching television) consistently correlates with negative cognitive outcomes, including cognitive decline and dementia risk. In contrast, active sedentary activities, such as reading, playing card games, and computer use relate to positive cognitive outcomes, particularly in preserving executive function, memory, and cognitive flexibility. These results suggest that public health guidelines should emphasize reducing time spent on passive sedentary activities and promoting cognitively stimulating activities to protect against cognitive decline. Additionally, future research should incorporate experimental studies to change sedentary activities, including cognitive training. These trials could also examine how physical activity might mitigate the cognitive risks associated with sedentary activities. Together, this body of research would provide evidence to gain a more comprehensive understanding of how to improve cognitive health in aging populations.

## Supplemental Material

sj-docx-1-alz-10.1177_13872877251394751 - Supplemental material for Individual sedentary activities and cognitive function in middle-aged and older adults: A systematic review Supplemental material, sj-docx-1-alz-10.1177_13872877251394751 for Individual sedentary activities and cognitive function in middle-aged and older adults: A systematic review by Jiatong Chen, Kirsten Dillon-Rossiter, Lily Grigsby-Duffy, Anisa Morava, Adam Novic, Babac Salmani, Siobhan Smith, Harry Prapavessis and Paul A Gardiner in Journal of Alzheimer's Disease
