# Supplementary material for: Individual sedentary activities and cognitive function in middle-aged and older adults: A systematic review
Source: J Alzheimers Dis. 2025 Dec 22;109(2):545–93. doi: 10.1177/13872877251394751 (PMC12932704; doi:10.1177/13872877251394751)
Supplement: sj-docx-1-alz-10.1177_13872877251394751 - Supplemental material for Individual sedentary activities and cognitive function in middle-aged and older adults: A systematic review [file sj-docx-1-alz-10.1177_13872877251394751.docx]

**Supplemental Material**

**Individual sedentary activities and cognitive function in middle-aged and older adults: A systematic review**

**Supplemental Table 1. PRISMA 2020 Checklist**

| **Section and Topic** | **Item #** | **Checklist item** | **Location where item is reported** |
| --- | --- | --- | --- |
| **TITLE** | | |  |
| Title | 1 | Identify the report as a systematic review. | 1 |
| **ABSTRACT** | | |  |
| Abstract | 2 | See the PRISMA 2020 for Abstracts checklist. | 2-3 |
| **INTRODUCTION** | | |  |
| Rationale | 3 | Describe the rationale for the review in the context of existing knowledge. | 4-7 |
| Objectives | 4 | Provide an explicit statement of the objective(s) or question(s) the review addresses. | 7 |
| **METHODS** | | |  |
| Eligibility criteria | 5 | Specify the inclusion and exclusion criteria for the review and how studies were grouped for the syntheses. | 8-12 |
| Information sources | 6 | Specify all databases, registers, websites, organisations, reference lists and other sources searched or consulted to identify studies. Specify the date when each source was last searched or consulted. | 8 |
| Search strategy | 7 | Present the full search strategies for all databases, registers and websites, including any filters and limits used. | Supplemental file 2 |
| Selection process | 8 | Specify the methods used to decide whether a study met the inclusion criteria of the review, including how many reviewers screened each record and each report retrieved, whether they worked independently, and if applicable, details of automation tools used in the process. | 11-12 |
| Data collection process | 9 | Specify the methods used to collect data from reports, including how many reviewers collected data from each report, whether they worked independently, any processes for obtaining or confirming data from study investigators, and if applicable, details of automation tools used in the process. | 12 |
| Data items | 10a | List and define all outcomes for which data were sought. Specify whether all results that were compatible with each outcome domain in each study were sought (e.g. for all measures, time points, analyses), and if not, the methods used to decide which results to collect. | 10-11 |
|  | 10b | List and define all other variables for which data were sought (e.g. participant and intervention characteristics, funding sources). Describe any assumptions made about any missing or unclear information. | 8-10 |
| Study risk of bias assessment | 11 | Specify the methods used to assess risk of bias in the included studies, including details of the tool(s) used, how many reviewers assessed each study and whether they worked independently, and if applicable, details of automation tools used in the process. | 13-14 |
| Effect measures | 12 | Specify for each outcome the effect measure(s) (e.g. risk ratio, mean difference) used in the synthesis or presentation of results. |  |
| Synthesis methods | 13a | Describe the processes used to decide which studies were eligible for each synthesis (e.g. tabulating the study intervention characteristics and comparing against the planned groups for each synthesis (item #5)). | 13-14 |
|  | 13b | Describe any methods required to prepare the data for presentation or synthesis, such as handling of missing summary statistics, or data conversions. | 13-14 |
|  | 13c | Describe any methods used to tabulate or visually display results of individual studies and syntheses. | 13-14 |
|  | 13d | Describe any methods used to synthesize results and provide a rationale for the choice(s). If meta-analysis was performed, describe the model(s), method(s) to identify the presence and extent of statistical heterogeneity, and software package(s) used. | 13-14 |
|  | 13e | Describe any methods used to explore possible causes of heterogeneity among study results (e.g. subgroup analysis, meta-regression). |  |
|  | 13f | Describe any sensitivity analyses conducted to assess robustness of the synthesized results. |  |
| Reporting bias assessment | 14 | Describe any methods used to assess risk of bias due to missing results in a synthesis (arising from reporting biases). |  |
| Certainty assessment | 15 | Describe any methods used to assess certainty (or confidence) in the body of evidence for an outcome. |  |
| **RESULTS** | | |  |
| Study selection | 16a | Describe the results of the search and selection process, from the number of records identified in the search to the number of studies included in the review, ideally using a flow diagram. | 14-15, Figure 1 |
|  | 16b | Cite studies that might appear to meet the inclusion criteria, but which were excluded, and explain why they were excluded. |  |
| Study characteristics | 17 | Cite each included study and present its characteristics. | Table 1 |
| Risk of bias in studies | 18 | Present assessments of risk of bias for each included study. | 32, Table 9 |
| Results of individual studies | 19 | For all outcomes, present, for each study: (a) summary statistics for each group (where appropriate) and (b) an effect estimate and its precision (e.g. confidence/credible interval), ideally using structured tables or plots. | Tables 2-8 |
| Results of syntheses | 20a | For each synthesis, briefly summarise the characteristics and risk of bias among contributing studies. | Table 9 |
|  | 20b | Present results of all statistical syntheses conducted. If meta-analysis was done, present for each the summary estimate and its precision (e.g. confidence/credible interval) and measures of statistical heterogeneity. If comparing groups, describe the direction of the effect. | Tables 2-8 |
|  | 20c | Present results of all investigations of possible causes of heterogeneity among study results. | Tables 2-8 |
|  | 20d | Present results of all sensitivity analyses conducted to assess the robustness of the synthesized results. | 32 |
| Reporting biases | 21 | Present assessments of risk of bias due to missing results (arising from reporting biases) for each synthesis assessed. |  |
| Certainty of evidence | 22 | Present assessments of certainty (or confidence) in the body of evidence for each outcome assessed. |  |
| **DISCUSSION** | | |  |
| Discussion | 23a | Provide a general interpretation of the results in the context of other evidence. | 32-78 |
|  | 23b | Discuss any limitations of the evidence included in the review. | 76-79 |
|  | 23c | Discuss any limitations of the review processes used. | 76-79 |
|  | 23d | Discuss implications of the results for practice, policy, and future research. | 76-82- |
| **OTHER INFORMATION** | | |  |
| Registration and protocol | 24a | Provide registration information for the review, including register name and registration number, or state that the review was not registered. | 8 |
|  | 24b | Indicate where the review protocol can be accessed, or state that a protocol was not prepared. | 8 |
|  | 24c | Describe and explain any amendments to information provided at registration or in the protocol. | 8 |
| Support | 25 | Describe sources of financial or non-financial support for the review, and the role of the funders or sponsors in the review. | 83 |
| Competing interests | 26 | Declare any competing interests of review authors. | 83 |
| Availability of data, code and other materials | 27 | Report which of the following are publicly available and where they can be found: template data collection forms; data extracted from included studies; data used for all analyses; analytic code; any other materials used in the review. | 83 |

**Supplemental Table 2. Search strategies applied to select studies**

| CINAHL | MH "Dementia+" OR MH "Intelligence+" OR MH "Memory+" OR "Alzheimer's Disease" OR MH" Language" OR "Spatial Processing" OR MH ""Psychomotor Performance+"OR MH "Attention+" OR "mental deterioration" OR "mental decline" OR "cognitive deterioration" OR "cognitive impairment" OR "cognitively impaired" OR "cognitive deficit" OR "cognitive dysfunction" OR cognition OR cognitive OR dementia OR "Alzheimer’s disease" OR "brain function" OR "executive function" OR "processing speed" OR memory OR "psychomotor speed" OR sensorimotor OR "Visual-spatial" OR Visuospatial OR attention OR language OR intelligence OR intellect  MH "Aging+" OR MH "aged+” OR MH "Middle Age" OR Elderly OR Senior OR seniors OR “older adult” OR “older adults” OR Elderly OR geriatric OR geriatrics OR “old people” OR “older age” OR “old age” OR TI adult OR TI adults OR “older people” OR elders OR “middle aged”  MH "Television" OR "driving time" OR “video gaming” OR “video game” OR “computer gaming” OR “video game time” OR “computer game” OR MH "Video Games+" OR “screen time” OR “computer time” OR “screen entertainment” OR “screen behaviour” OR screen behavior OR screen-based entertainment OR television viewing OR television watching” OR “television time” OR “TV viewing” OR “TV watching” OR “TV time” OR “watch television” OR “view television” OR “watch TV” OR “screen watching” OR “screen time” OR “screen entertainment” OR “screen behaviour” OR “screen behavior” OR “screen-based entertainment” OR Sudoku OR crossword OR “arts and crafts” OR “card games” OR puzzles OR “Board games” OR “sedentary behavior” OR “sedentary behaviour” OR “sedentary lifestyle” OR “prolonged sitting” OR “sitting time” OR “lying time” OR “lying down” OR “office work” OR MH "Life Style, Sedentary" |
| --- | --- |
| Web of Science | TS = ("aging" OR "middle aged" OR Elderly OR Senior OR seniors OR "older adult" OR "older adults" OR Elderly OR geriatric OR geriatrics OR "old people" OR "older age" OR "old age" OR "older people" OR elders OR "middle aged" OR adult OR adults)  TS= (dementia OR Intelligence OR Memory OR "Alzheimer Disease" OR Language OR "Spatial Processing" OR "Psychomotor Performance" OR Attention OR "mental deterioration" OR "mental decline" OR "cognitive deterioration" OR "cognitive impairment" OR "cognitively impaired" OR "cognitive deficit" OR "cognitive dysfunction" OR cognition OR cognitive OR dementia OR "alzheimers disease" OR "brain function" OR "executive function" OR "processing speed" OR sensorimotor OR "Visual-spatial" OR Visuospatial OR attention OR intellect)  TS= (television OR "driving time" OR "video gaming" OR “video game” OR “computer gaming” OR “computer game” OR “video game time” OR computers OR "screen time" OR "computer time" OR "screen entertainment" OR "screen behavior" OR "screen behaviour" OR "screen-based entertainment" OR "television viewing" OR "television watching" OR "television time" OR "TV viewing" OR "TV watching" OR "TV time" OR "watch television" OR "view television" OR "watch TV" OR "screen watching" OR "screen time" OR "screen entertainment" OR "screen behavior" OR "screen behavior" OR "screen-based entertainment" OR sudoku OR crossword OR "arts and crafts" OR "card games" OR puzzles OR "Board games" OR "sedentary behavior" OR "sedentary behavior" OR "sedentary lifestyle" OR "prolonged sitting" OR "sitting time" OR "lying time" OR "lying down" OR "office work" OR "sedentary lifestyle") |
| SCOPUS | ( TITLE-ABS-KEY ( "aging"  OR  "aged"  OR  "middle aged"  OR  elderly  OR  senior  OR  seniors  OR  "older adult"  OR  "older adults"  OR  elderly  OR  geriatric  OR  geriatrics  OR  "old people"  OR  "older-age"  OR  "old age"  OR  adult  OR  adults  OR  "older people"  OR  elders  OR  middle  AND  aged ) )  AND  ( TITLE-ABS-KEY ( car  OR  automobile*  OR  riding  OR  car  OR  automobile*  OR  television  OR  "driving time"  OR  "video gaming"  OR  video  AND  game  OR  computer  AND  gaming  OR  video  AND  game  AND  time  OR  computer  AND  game  OR  computers  OR  "Video Games"  OR  "screen time"  OR  "computer time"  OR  "screen entertainment"  OR  "screen behavior"  OR  "screen behavior"  OR  "screen-based entertainment"  OR  "television viewing"  OR  "television watching"  OR  "television time"  OR  "TV viewing"  OR  "TV watching"  OR  "TV time"  OR  "watch television"  OR  "view television"  OR  "watch TV"  OR  "screen watching"  OR  "screen time"  OR  "screen entertainment"  OR  "screen behavior"  OR  "screen behavior"  OR  "screen-based entertainment"  OR  sudoku  OR  crossword  OR  "arts and crafts"  OR  "card games"  OR  puzzles  OR  "Board games"  OR  "sedentary behavior"  OR  "sedentary behavior"  OR  "sedentary lifestyle"  OR  "prolonged sitting"  OR  "sitting time"  OR  "lying time"  OR  "lying down"  OR  "office work"  OR  "sedentary lifestyle" ) )  AND  ( TITLE-ABS-KEY ( dementia  OR  intelligence  OR  memory  OR  "Alzheimer Disease"  OR  "Spatial Processing"  OR  "Psychomotor Performance"  OR  attention  OR  "mental deterioration"  OR  "mental decline"  OR  "cognitive deterioration"  OR  "cognitive impairment"  OR  "cognitively impaired"  OR  "cognitive deficit"  OR  "cognitive dysfunction"  OR  cognition  OR  cognitive  OR  dementia  OR  "alzheimer's disease"  OR  "brain function"  OR  "executive function"  OR  "processing speed"  OR  memory  OR  "psychomotor speed"  OR  sensorimotor  OR  "Visual-spatial"  OR  visuospatial  OR  attention  OR  language  OR  intellect ) )  AND  ( LIMIT-TO ( LANGUAGE ,  "English" ) )  AND  ( LIMIT-TO ( SRCTYPE ,  "j" ) ) |
| SPORTDiscus | "aging" OR "middle aged" OR Elderly OR Senior OR seniors OR "older adult" OR "older adults" OR Elderly OR geriatric OR geriatrics OR "old people" OR "older-age" OR "old age" OR "older people" OR elders OR "middle aged" OR adult OR adults  dementia OR Intelligence OR Memory OR "Alzheimer Disease" OR Language OR "Spatial Processing" OR "Psychomotor Performance" OR Attention OR "mental deterioration" OR "mental decline" OR "cognitive deterioration" OR "cognitive impairment" OR "cognitively impaired" OR "cognitive deficit" OR "cognitive dysfunction" OR cognition OR cognitive OR dementia OR "alzheimers disease" OR "brain function" OR "executive function" OR "processing speed" OR sensorimotor OR "Visual-spatial" OR Visuospatial OR attention OR intellect  Television OR "driving time" OR "video gaming" OR “video game” OR “computer gaming” OR “computer game” OR “video game time” OR computers OR "screen time" OR "computer time" OR "screen entertainment" OR "screen behavior" OR "screen behaviour" OR "screen-based entertainment" OR "television viewing" OR "television watching" OR "television time" OR "TV viewing" OR "TV watching" OR "TV time" OR "watch television" OR "view television" OR "watch TV" OR "screen watching" OR "screen time" OR "screen entertainment" OR "screen behavior" OR "screen behavior" OR "screen-based entertainment" OR sudoku OR crossword OR "arts and crafts" OR "card games" OR puzzles OR "Board games" OR "sedentary behavior" OR "sedentary behavior" OR "sedentary lifestyle" OR "prolonged sitting" OR "sitting time" OR "lying time" OR "lying down" OR "office work" OR "sedentary lifestyle" |
| APA PsychINFO: | (Aging/ or TI "middle aged" OR AB "middle aged" or TI " elders" OR AB "elders" or TI " old age" OR AB "old age" or Geriatrics/ or Geriatric Patients or older Adulthood/ or TI" older people' or AB"older people" or TI " older age" OR AB "older age" or TI" geriatric" or AB geriatric" or Geriatrics/ or TI"geriatrics" or AB"geriatrics" or TI"older adults" or AB"older adults" or TI"older adult" or AB"older adult" or TI"seniors" or AB"seniors" or TI"senior" OR AB"senior" or TI"elderly" or AB"elderly" or TI"aged" or AB"aged" or TI"ageing" or AB"ageing") and (Sedentary Behavior/ or TI"sedentary behaviour" or AB"sedentary behaviour" or Posture/ or TI"prolonged sitting" or AB" prolonged sitting" or TI"sitting time" or AB"sitting time" or TI"lying time" or AB"lying time" or TI"lying down" or AB"lying down" or TI"office work" or AB"office work" or TI"sedentary lifestyle" or AB"sedentary lifestyle" or TI"sedentary time" or AB"sedentary time" or TI"sitting position" or AB"sitting position" or TI"sedentary behavior" or AB "sedentary behavior") and (Intelligence/ or TI"intelligence" or AB "intelligence" or TI"memory" or AB "memory" or exp Wechsler Memory Scale/ or exp Spatial Memory/ or exp Episodic Memory/ or exp Implicit Memory/ or exp Memory/ or exp Short Term Memory/ or exp Verbal Memory/ or exp Long Term Memory/ or exp Associative Memory/ or exp Visuospatial Memory/ or exp Prospective Memory/ or exp Semantic Memory/ or exp Visual Memory/ or TI"language" or AB "language" or Language/ or Spatial Ability/ or Spatial Perception/ or Cognitive processes/ or Visual Perception/ or Attention/ or Visuospatial Ability/ or TI"spatial processing" or AB "spatial processing" or Cognitive Ability/ or Motor Performance/ or reaction Time/ or TI"psychomotor performance" or AB" psychomotor performance" or Neuropsychology/ or TI" mental deterioration" or AB " mental deterioration" or exp Dementia/ or exp Cognitive Impairment/ or exp Neurodegenerative Diseases/ or exp Alzheimer's Disease/ or TI" mental decline" or AB" mental decline" or TI"cognitive dysfunction" or AB"cognitive dysfunction" or TI"cognition" or AB "cognition" or Cognition/ or Brain/ or Cognitive Processes/ or brain function.mp. or Cognitive Ability/ or TI"processing speed" or AB"processing speed" or Neuropsychological Assessment/ or TI"psychomotor speed" or AB" psychomotor speed" or TI"Visuospatial" or AB "Visuospatial" or alzheimer's disease/ or neurodegenerative diseases/ or TI"executive function" or AB "executive function" or exp Executive Function/) and (TI"automobile" or AB automobile" or Automobiles/ or Transportation/ or Drivers/ TI"car" or AB "car" or Television Viewing/ or Television/ or TI"television" or AB "television" or TI"driving time" or AB "driving time" or Digital Gaming/ or Computer Games/ or TI"video gaming" or AB "video gaming" or TI"computer" or AB "computer" or TI"computer time" or AB "computer time" or technology/ or Screen Time/ or TI"screen time" OR AB" screen time" or Mobile Applications/ or TI"screen-based entertainment" or AB"screen-based entertainment" or television/ or TI"TV" or AB "TV" or Games/ or TI"sudoku" or AB "sudoku" or TI"crossword" or AB "crossword" or TI"arts and crafts" or AB" arts and crafts" or TI"card games" or AB "card games" or TI"puzzles" or AB "puzzles" or TI"board games" or AB "board games") |
| MEDLINE | (Sedentary behavior.mp. or Sedentary behavior/ or sedentary behaviour.mp. or Sitting Position/ or Posture/ or prolonged sitting.mp. or sitting time.mp. or lying time.mp. or lying down.mp. or Workplace/ or office work.mp. or sedentary lifestyle.mp. or sitting position.mp. or Sitting Position/ or lying position.mp.) and (driving.mp. or Automobile Driving/ or riding.mp. or car.mp. or Automobiles/ or Television.mp. or Television/ or driving time.mp. or Video Games/ or video gaming.mp. or Computers/ or computer gaming.mp. or television viewing.mp. or television watching.mp. or TV viewing.mp. or screen watching.mp. or screen time.mp. or Screen Time/ or screen entertainment.mp. or sodoku.mp. or Games, Recreational/ or card games.mp. or puzzles.mp. or board games.mp. or crossword.mp.) and (middle aged.mp. or Middle Aged/ or Middle Aged/ or elders.mp. or Humans/ or Aging/ or Adult/ or Aged/ or Middle Aged/ or Aged/ or Aging/ or Humans/ or older people.mp. or old age.mp. or older-age.mp. or old people.mp. geriatrics.mp. or Geriatrics/ or geriatric.mp. or Geriatrics/ or older adult.mp. or older adults.mp. or Middle Aged/ or senior.mp. or Humans/ or Aged/ or Senior Centers/ or Adult/ or seniors.mp. or elderly.mp. or Aged/ or aged.mp. or Middle Aged/ or Aged/ or Aging/ or aging.mp. or Healthy Aging/ or Aging/ or ageing.mp.) and (Intelligence/ or Intelligence.mp. or Memory, Short-Term/ or " Memory and Learning Tests"/ or memory.mp. or Memory, Episodic/ or Memory Consolidation/ or Memory, Long-Term/ or Alzheimer Disease.mp. or Alzheimer Disease/or Language/ or language.mp. or Orientation/ or Visual Perception/ or spatial processing.mp. or Spatial Processing/ or Psychomotor Performance.mp. or Psychomotor Performance/ or attention.mp. or Attention/ or mental deterioration.mp. or Cognitive Dysfunction/ or Dementia/ or mental decline.mp. or Cognition/ or Cognition Disorders/ or Brain/ or cognitive deterioration.mp. or cognitive impairment.mp. or cognitively impaired.mp. or Cognition Disorders/ or cognitive deficit.mp. Cognition/ or cognition.mp. or cognitive.mp. or brain function.mp. or executive function.mp. or executive Function/ or Neuropsychological Tests/ or processing speed.mp. or Psychomotor Performance/ or psychomotor speed.mp. or sensorimotor.mp. or Visual Perception/ or Visual-spatial.mp. or Space Perception/ Visuospatial.mp.) |
| Embase | (Middle aged.mp. or middle aged/ or aging/ or elders.mp. or aged/ or elderly care/ or older people.mp. or old age.mp. or adult/ or aged/ or older-age.mp. or age/or aging/ or very elderly/ or old people/mp. or geriatric.mp. or geriatrics/ or geriatric.mp. or geriatrics/ or older adult.mp. or older adults.mp. or seniros.mp. or senior centre/ or residents/ or senior.mp. or elderly.mp. or aged/ or aging.mp. or aging/ or ageing.mp) and (sedentary behaviour.mp. or sedentary lifestyle/ or sedentary behavior.mp. or sitting position.mp. or sitting/ prolonged sitting.mp. or body posture/ sitting time.mp. or sedentary time/ or lying time.mp. or lying down.mp. or office worker/ or office work.mp. or sitting/) and (intelligence test/ or Wechsler adult intelligence scale/ or intelligence. or intelligence.mp. or Wechsler intelligence scale/ or memory assessment/ or retrospective memory/ or word memory test/ or semantic memory/ or associative memory/ or Wechsler memory scale/ or sensory memory/ or visual memory/ or spatial memory/ or verbal memory/ or nonverbal memory test/ or verbal memory test/ or implicit memory/ or declarative memory/ or long-term memory/ or procedural memory/ or prospective memory/ or non-spatial memory test/ or episodic memory/ or memory.mp. or short-term memory/ or “learning and memory test”/ or spatial memory test/ or memory/ or memory test/ or working memory/ or explicit memory/ or Alzheimer Disease.mp. or Alzheimer disease/ or language test/ or language.mp. or language ability/ or language. or language processing/ or language delay/ or spatial processing.mp. or spatial behaviour/ psychomotor performance.mp. or psychomotor performance/ or attention test/ or attention/ or attention.mp. or visual attention/ mental deterioration.mp. or mental deterioration/ cognitive defect/ or dementia/ or mental decline.mp. or cognitive deterioration.mp. or cognitive impairment.mp. or cognitively impaired.mp. or cognitive deficit.mp. or cognitive dysfunction.mp. or cognition.mp. cognition assessment/ or cognition/ or cognitive function test/ or cognitive aging/ or cognitive.mp. or cognitive neuroscience/ or mild cognitive impairment/ or brain function.mp. or brain function/ or executive function. mp. or executive function/or cognition/ or information processing/ or processing speed.mp. processing speed/ reaction time or working memory/ or psychomotor performance/ or psychomotor speed.mp. or sensorimotor function/ or sensorimotor.mp. or Visual-spatial.mp. or visuospatial.mp. or task performance/) and driving.mp. or car driving/ or riding.mp. or car.mp. or car/ or automobile.mp. or television viewing/ or television/ or television.mp. or driving time.mp. or video gaming.mp. or video game/ or computer/ or computer.mp. or video game time.mp. or computer game.mp. or television/ or TV viewing.mp. or television viewing/ TV time.mp. or screen time.mp. or screen time/ crossword.mp. sudoku.mp. or card games.mp. puzzles.mp. board games.mp. ) |

**Supplemental Table 3. Categorization of cognitive domain**

| Domain | Definition | Cognitive tests |
| --- | --- | --- |
| Processing Speed | Processing speed refers to assessments of cognitive functioning that require individuals to complete tasks swiftly, ranging from very basic to more complex activities. Common tasks to measure processing speed include coding tasks like Symbol Coding and tests that involve quickly connecting numbers or alternating sequences of letters and numbers, such as the Trail Making Test. The key aspect of these tasks is that participants are explicitly instructed to complete them as fast as possible. Performance is typically measured by either the time taken to complete the task or the number of correct responses. Although these tasks are generally simple, they may involve some elements of learning (e.g., matching symbols to digits) or executive function challenges, such as alternating between different types of information ^1^. | Animal fluency  Block Design  Cognitrone test  Detection test  Digit Symbol  Digit symbol Coding  Digit symbol substitution task  Digits forward  Four-choice RT  Identification test  Letter comparison  Letter fluency  Paced auditory serial addition test  Paced Auditory Serial Addition Test  Pattern comparison  Semantic fluency  Stroop 1  Symbol digit modalities test  Trail making test A  Vegetable fluency  Word fluency |
| Executive function | Executive function, often referred to as reasoning and problem-solving, encompasses a set of cognitive processes that govern and control other mental abilities, enabling individuals to efficiently use their cognitive resources to solve problems and plan for the future. Tasks like problem-solving, planning, navigating mazes, and other complex activities that require the coordination of multiple cognitive skills fall within this domain. Executive function is characterized as a top-down process because it requires the integration of simpler cognitive abilities for success in real-world scenarios. It also involves cognitive flexibility, as solving novel problems often demands the ability to adapt strategies and quickly abandon ineffective ones. Classic tests of executive function, such as the Wisconsin Card Sorting Test (WCST), assess problem-solving in response to feedback. A common issue in such tasks is the production of perseverative errors, where individuals continue to make the same mistakes despite feedback that indicates their approach is incorrect ^1^. | Groton maze learning test  Attention network test  Attentional performance battery  Colour word inhibition  Executive function score from the following: trail making test, verbal fluency from the Delis Kaplan Executive Function System, digit span backwards and mental arithmetic from Wecshler Adult Intelligence Scale, Wisconsin Card Sorting Test.  Flanker or Erikson flanker test  Flexibility Score from the Computer-based Test of Attentional Performance  Mazes  Stroop  Symbol cancellation |
| Working memory | Working memory refers to the capacity to retain information in active awareness for adaptive purposes. It involves integrating input from various sensory modalities, including both verbal and nonverbal data. Additionally, working memory is understood to consist of two distinct components: the ability to maintain information and the ability to manipulate that information for use ^1^. | Automated operation span  Colour word inhibition  Computer based task  Design memory  Digit span backwards  Digits backwards  Letter number sequencing  List sorting  N-back  Symbol trials  Trail making test B |
| Cognitive flexibility | Cognitive flexibility refers to the capacity to modify one’s behavior in response to changing circumstances. It allows individuals to efficiently shift focus from a prior task, adopt a new set of responses, and apply this new approach to the current task. Enhanced cognitive flexibility has been linked to various positive outcomes across the lifespan, including improved reading skills in childhood, greater resilience to stress and negative life events in adulthood, increased creativity, and a higher quality of life in older age. Despite the significant implications of cognitive flexibility throughout life, its in-depth study has been challenging ^2^. | Letter number sequencing  Symbol cancellation  Symbol Trials  Task Switching  Task switching paradigm  The dimensional change card sort test  Trial making test B  Wisconsin card sorting task |
| Global Cognitive Function | Global cognition refers to an overall measure of cognitive ability, calculated by combining performance across various cognitive domains. Comprehensive assessments such as the Wechsler Adult Intelligence Scale are often used, while smaller tools like the Mini-Mental State Examination (MMSE) are applied to evaluate mental status ^3^. | 3MS  Addenbrooke’s Cognitive Examination  Addenbrooke’s Cognitive Examination III  Alzheimer’s Disease Assessment Scale-Cognitive plus  Chinese version of the Ascertain Dementia 8-item questionnaire  Cognitive function overall score  Combined score from: Digit Symbol Coding, Hopkins Verbal Learning Test, n-back, Task switching paradigm)  g-factor test battery: Mini-Mental State Examination, Stroop, letter-digit substitution task, Verbal Fluency 15-word learning test, Purdue pegboard test)  General cognitive ability factor (g) computed from 6 tests taken from the Wecshler Adult Intelligence Scale (Matrix Reasoning, Block Design, Letter-Number Sequencing, Symbol Search, Digit Span Backwards, and Digit Symbol), Moray Houst Test No. 12, Alice Heim 4 test)  Global composite: Digit Symbol Coding, Hopkins Verbal Learning Test, n-back, Task switching paradigm  Hong Kong version of Montreal Cognitive Assessment  Items from Montreal Cognitive Assessment  Mini-Mental State Examination  Modified Mini-Mental State Examination  Montreal Cognitive Assessment  Recommendations of the National Institute on Aging- Alzheimer’s Association  Telephone assessment and interview for Cognitive Status (orientation, serial subtraction, word recall, semantics, sentence repetition, linguistic skills, and attention) |
| Episodic memory | This component of the memory system works in conjunction with working memory to encode, store, and retrieve information from long-term memory. It handles input from various sensory modalities, including both verbal and nonverbal information. For example, episodic memory encompasses the recollection of personal experiences, such as remembering what you did the previous evening or recalling the details of your last meal ^1^. | Brief Spanish English Verbal learning Test  Brief Visuospatial Memory Test- Revised  California Verbal Learning Test  California Verbal Learning Test- II  Face naming score  Faces subtest from the Wechsler Memory Scale-Third Edition  Free Recall  Hopkins Verbal Learning Test - Revised  Immediate and delayed recall  Logical memory delayed  Logical memory immediate  Logical memory recall  Picture sequence memory test  Selective reminding scores  Verbal memory  Verbal memory Z score  Verbal paired association  Wechsler Memory Scale- revised  Word Recognition |
| Motor skills and construction | Motor skills consist of several fundamental aspects of motor activity, including fine motor skills such as manual dexterity and motor speed, as well as reaction time. Additionally, they involve broader abilities like balance and coordination ^1^. | Dear- Leiwald Reaction task  Grooved Pegboard Test  Reaction Test  Reaction time from the computer- based Test of Attentional Performance  Rey Complex Figure  Simple Reaction Time |

**Supplemental Table 4. Categorization of impaired cognition**

| Domain | Definition | Cognitive tests |
| --- | --- | --- |
| Dementia | Dementia refers to a group of diseases that impair memory, cognitive function, and the ability to perform daily tasks ^4^. | A list of 10 words CERAD battery and the CSI-D  Hospital inpatient records  MMSE |
| Cognitive impairment | Cognitive impairment refers to difficulties with thinking, learning, remembering, exercising judgment, and making decisions^5^. | A list of 10 words CERAD battery and the CSI-D  Immediate recall of a 10-word list  MMSE  MoCA  Impairment Screen (MIS) test  Spanish version of Short Portable Mental State Questionnaire  Semantic Verbal Fluency (SVF) test  Kihon Checklist (KCL) |

**Supplemental Table 5. Quality assessment of included studies**

| References | 1.  Question/  objective sufficiently described. | 2.  Appropriate  study design | 3.  Appropriate method/  source of selection | 4.  Characteristic sufficiently described | 5.  Random allocation described. | 6.  Blinding  of  investigator | 7.  Blinding  of subjects | 8a.  Outcome measures defined and robust | 8b.  Exposure measures  Defined  and robust | 9.  Appropriate sample size | 10.  Appropriate analytical methods | 11.  Estimate of variance reported | 12.  Controlled  for confounding | | 13.  Results reported sufficiently | | 14.  Conclusions supported by results | | 15.  Quality score  (%) | | 16.  Quality  strength | |  |
| --- | --- | --- | --- | --- | --- | --- | --- | --- | --- | --- | --- | --- | --- | --- | --- | --- | --- | --- | --- | --- | --- | --- | --- |
| Allen et al. ^6^ | 1 | 2 | 2 | 1 | n/a | n/a | n/a | 1 | 1 | 2 | 2 | 1 | | 2 | | 2 | | 2 | | 79.2 | | Good | |
| Anaraky et al. ^7^ | 2 | 1 | 2 | 2 | n/a | n/a | n/a | 1 | 1 | 2 | 2 | 1 | | 2 | | 1 | | 2 | | 79.2 | | Good | |
| Anaturk et al. ^8^ | 2 | 1 | 2 | 2 | n/a | n/a | n/a | 1 | 1 | 2 | 2 | 2 | | 2 | | 1 | | 2 | | 83.3 | | Strong | |
| Bakrania et al. ^9^ | 2 | 2 | 1 | 2 | n/a | n/a | n/a | 1 | 1 | 2 | 2 | 2 | | 2 | | 2 | | 2 | | 87.5 | | Strong | |
| Bernstein et al. ^10^ | 2 | 2 | 2 | 1 | n/a | n/a | n/a | 1 | 1 | 1 | 1 | 1 | | 0 | | 1 | | 2 | | 62.5 | | Good | |
| Bernstein et al. ^11^ | 2 | 1 | 1 | 2 | n/a | n/a | n/a | 1 | 1 | 1 | 1 | 1 | | 0 | | 1 | | 2 | | 58.3 | | Adequate | |
| Brooker et al. ^12^ | 1 | 1 | 1 | 1 | n/a | n/a | n/a | 1 | 1 | 2 | 1 | 1 | | 1 | | 1 | | 2 | | 58.3 | | Adequate | |
| Brooker et al. ^13^ | 1 | 1 | 1 | 1 | n/a | n/a | n/a | 1 | 1 | 2 | 1 | 1 | | 1 | | 1 | | 2 | | 58.3 | | Adequate | |
| Cansino et al. ^14^ | 2 | 2 | 2 | 2 | n/a | n/a | n/a | 1 | 1 | 2 | 2 | 1 | | 1 | | 2 | | 2 | | 83.3 | | Strong | |
| Cegolon et al. ^15^ | 2 | 1 | 2 | 1 | n/a | n/a | n/a | 1 | 1 | 2 | 2 | 1 | | 2 | | 2 | | 2 | | 79.2 | | Good | |
| Chen et al. ^16^ | 2 | 1 | 2 | 2 | n/a | n/a | n/a | 1 | 1 | 2 | 2 | 2 | | 2 | | 2 | | 2 | | 87.5 | | Strong | |
| Choi et al. ^17^ | 2 | 2 | 1 | 2 | n/a | n/a | n/a | 1 | 1 | 2 | 2 | 2 | | 2 | | 2 | | 2 | | 83.3 | | Strong | |
| Coelho et al.^18^ | 2 | 2 | 1 | 1 | n/a | n/a | n/a | 1 | 1 | 1 | 1 | 1 | | 1 | | 1 | | 2 | | 70.8 | | Good | |
| Covey et al. ^19^ | 2 | 1 | 1 | 2 | n/a | n/a | n/a | 1 | 1 | 1 | 2 | 1 | | 1 | | 1 | | 2 | | 66.7 | | Good | |
| Cutting et al. ^20^ | 1 | 1 | 2 | 0 | n/a | n/a | n/a | 1 | 1 | 2 | 1 | 1 | | 0 | | 1 | | 2 | | 54.2 | | Adequate | |
| Da Ronch et al. ^21^ | 1 | 2 | 2 | 1 | n/a | n/a | n/a | 1 | 1 | 2 | 1 | 2 | | 2 | | 1 | | 2 | | 75 | | Good | |
| Fajersztajn et al. ^22^ | 2 | 2 | 2 | 2 | n/a | n/a | n/a | 1 | 1 | 2 | 2 | 1 | | 2 | | 1 | | 2 | | 83.3 | | Strong | |
| Fancourt et al. ^23^ | 2 | 2 | 2 | 2 | n/a | n/a | n/a | 1 | 1 | 2 | 2 | 1 | | 2 | | 1 | | 2 | | 83.3 | | Strong | |
| Feng et al. ^24^ | 2 | 2 | 2 | 2 | n/a | n/a | n/a | 1 | 1 | 1 | 1 | 1 | | 1 | | 1 | | 2 | | 70.8 | | Good | |
| Floud et al. ^25^ | 1 | 2 | 2 | 2 | n/a | n/a | n/a | 1 | 1 | 2 | 1 | 1 | | 2 | | 1 | | 1 | | 70.8 | | Good | |
| Green et al. ^26^ | 2 | 2 | 2 | 2 | n/a | n/a | n/a | 1 | 1 | 1 | 1 | 1 | | 1 | | 1 | | 2 | | 62.5 | | Good | |
| Hamer et al. ^27^ | 2 | 2 | 2 | 1 | n/a | n/a | n/a | 1 | 1 | 2 | 2 | 2 | | 2 | | 1 | | 1 | | 79.2 | | Good | |
| Hartanto et al. ^28^ | 2 | 2 | 2 | 2 | n/a | n/a | n/a | 1 | 1 | 2 | 2 | 2 | | 2 | | 1 | | 2 | | 87.5 | | Strong | |
| Heisz et al. ^29^ | 1 | 1 | 1 | 1 | n/a | n/a | n/a | 1 | 1 | 1 | 2 | 2 | | 1 | | 1 | | 2 | | 66.7 | | Good | |
| Hoang et al. ^30^ | 1 | 2 | 2 | 1 | n/a | n/a | n/a | 1 | 1 | 2 | 1 | 1 | | 2 | | 2 | | 2 | | 75 | | Good | |
| Hou et al. ^31^ | 1 | 1 | 1 | 1 | n/a | n/a | n/a | 1 | 1 | 1 | 1 | 1 | | 0 | | 1 | | 1 | | 45.8 | | Poor | |
| Ihle et al. ^32^ | 2 | 1 | 1 | 1 | n/a | n/a | n/a | 1 | 1 | 2 | 1 | 2 | | 1 | | 2 | | 2 | | 70.8 | | Good | |
| Ivleva et al. ^33^ | 1 | 2 | 1 | 1 | n/a | n/a | n/a | 1 | 1 | 2 | 2 | 2 | | 2 | | 1 | | 2 | | 75 | | Good | |
| Janoutová et al. ^34^ | 1 | 1 | 1 | 1 | n/a | n/a | n/a | 1 | 1 | 1 | 1 | 1 | | 1 | | 1 | | 2 | | 54.2 | | Adequate | |
| Jia et al. ^35^ | 2 | 2 | 2 | 2 | n/a | n/a | n/a | 1 | 1 | 2 | 2 | 1 | | 1 | | 1 | | 2 | | 79.2 | | Strong | |
| Jopp et al. ^36^ | 2 | 2 | 1 | 2 | n/a | n/a | n/a | 1 | 1 | 2 | 2 | 1 | | 2 | | 2 | | 2 | | 83.3 | | Strong | |
| Jung et al. ^37^ | 2 | 1 | 2 | 2 | n/a | n/a | n/a | 1 | 1 | 2 | 2 | 2 | | 2 | | 2 | | 2 | | 87.5 | | Strong | |
| Karsazi et al. ^38^ | 2 | 2 | 2 | 2 | n/a | n/a | n/a | 1 | 1 | 2 | 2 | 2 | | 1 | | 2 | | 2 | | 87.5 | | Strong | |
| Kesse-guyot et al. ^39^ | 1 | 2 | 2 | 2 | n/a | n/a | n/a | 1 | 1 | 2 | 2 | 2 | | 2 | | 1 | | 2 | | 83.3 | | Strong | |
| Kim et al. ^40^ | 2 | 1 | 1 | 1 | n/a | n/a | n/a | 1 | 1 | 1 | 2 | 1 | | 0 | | 1 | | 2 | | 58.3 | | Adequate | |
| Krell-Roesch et al. ^41^ | 2 | 2 | 2 | 1 | n/a | n/a | n/a | 1 | 1 | 2 | 1 | 1 | | 2 | | 1 | | 2 | | 75 | | Strong | |
| Krell-Roesch et al. ^42^ | 2 | 2 | 2 | 1 | n/a | n/a | n/a | 1 | 1 | 2 | 1 | 1 | | 2 | | 1 | | 2 | | 75 | | Strong | |
| Kurita et al. ^43^ | 2 | 2 | 2 | 2 | n/a | n/a | n/a | 1 | 1 | 2 | 1 | 1 | | 1 | | 1 | | 2 | | 75 | | Good | |
| Kurita et al. ^44^ | 1 | 2 | 1 | 2 | n/a | n/a | n/a | 1 | 1 | 2 | 2 | 1 | | 2 | | 1 | | 2 | | 75 | | Good | |
| Lindstrom et al. ^45^ | 1 | 2 | 2 | 1 | n/a | n/a | n/a | 1 | 1 | 2 | 2 | 2 | | 1 | | 1 | | 1 | | 70.8 | | Good | |
| Lin et al. ^46^ | 2 | 2 | 2 | 2 | n/a | n/a | n/a | 1 | 1 | 2 | 2 | 2 | | 2 | | 1 | | 2 | | 87.5 | | Strong | |
| Massakkers et al. ^47^ | 1 | 2 | 2 | 2 | n/a | n/a | n/a | 1 | 1 | 2 | 2 | 2 | | 2 | | 2 | | 2 | | 87.5 | | Strong | |
| Maasakkers et al. ^48^ | 2 | 2 | 2 | 2 | n/a | n/a | n/a | 1 | 1 | 2 | 2 | 2 | | 2 | | 1 | | 2 | | 87.5 | | Strong | |
| Mai et al. ^49^ | 2 | 2 | 2 | 1 | n/a | n/a | n/a | 1 | 1 | 1 | 2 | 2 | | 1 | | 1 | | 2 | | 75 | | Good | |
| Major et al. ^50^ | 2 | 2 | 2 | 1 | n/a | n/a | n/a | 1 | 1 | 2 | 2 | 1 | | 2 | | 2 | | 2 | | 83.3 | | Strong | |
| Mao et al. ^51^ | 2 | 1 | 2 | 2 | n/a | n/a | n/a | 1 | 1 | 2 | 2 | 2 | | 2 | | 2 | | 2 | | 87.5 | | Strong | |
| Mariano et al. ^52^ | 2 | 2 | 1 | 2 | n/a | n/a | n/a | 1 | 1 | 2 | 2 | 1 | | 2 | | 2 | | 2 | | 83.3 | | Strong | |
| Mariano et al. ^53^ | 2 | 2 | 2 | 1 | n/a | n/a | n/a | 1 | 1 | 2 | 2 | 2 | | 1 | | 1 | | 2 | | 79.2 | | Strong | |
| Mellow et al. ^54^ | 2 | 1 | 1 | 2 | n/a | n/a | n/a | 1 | 1 | 2 | 2 | 1 | | 2 | | 1 | | 2 | | 75 | | Good | |
| Miller et al. ^55^ | 2 | 2 | 2 | 2 | n/a | n/a | n/a | 1 | 1 | 2 | 2 | 2 | | 2 | | 1 | | 2 | | 87.5 | | Strong | |
| Nemoto et al. ^56^ | 2 | 1 | 1 | 2 | n/a | n/a | n/a | 1 | 1 | 2 | 2 | 2 | | 1 | | 1 | | 2 | | 75 | | Good | |
| Nemoto et al. ^57^ | 2 | 2 | 1 | 2 | n/a | n/a | n/a | 1 | 1 | 2 | 2 | 2 | | 2 | | 2 | | 1 | | 83.3 | | Strong | |
| Olanrewaju et al. ^58^ | 2 | 2 | 2 | 2 | n/a | n/a | n/a | 1 | 1 | 2 | 2 | 1 | | 2 | | 2 | | 2 | | 87.5 | | Strong | |
| Raichlen et al. ^59^ | 1 | 1 | 1 | 2 | n/a | n/a | n/a | 1 | 1 | 2 | 2 | 1 | | 1 | | 1 | | 2 | | 66.7 | | Good | |
| Ramos et al. ^60^ | 2 | 1 | 1 | 1 | n/a | n/a | n/a | 1 | 1 | 2 | 1 | 1 | | 2 | | 1 | | 2 | | 66.7 | | Good | |
| Rawtaer et al. ^61^ | 2 | 2 | 1 | 2 | n/a | n/a | n/a | 1 | 1 | 1 | 1 | 0 | | 0 | | 1 | | 2 | | 58.3 | | Adequate | |
| Ringin et al. ^62^ | 2 | 2 | 2 | 1 | n/a | n/a | n/a | 1 | 1 | 2 | 2 | 1 | | 2 | | 1 | | 2 | | 79.2 | | Good | |
| Rosenberg et al. ^63^ | 2 | 2 | 2 | 2 | n/a | n/a | n/a | 1 | 1 | 2 | 1 | 2 | | 2 | | 2 | | 2 | | 83.3 | | Strong | |
| Schaham et al. ^64^ | 2 | 2 | 1 | 1 | n/a | n/a | n/a | 1 | 1 | 1 | 2 | 2 | | 0 | | 2 | | 2 | | 79.2 | | Good | |
| Sha et al. ^65^ | 2 | 2 | 1 | 2 | n/a | n/a | n/a | 1 | 0 | 2 | 2 | 2 | | 2 | | 1 | | 2 | | 79.2 | | Good | |
| Shi et al. ^66^ | 1 | 1 | 1 | 1 | n/a | n/a | n/a | 1 | 1 | 2 | 2 | 2 | | 2 | | 1 | | 2 | | 70.8 | | Good | |
| Shimada et al. ^67^ | 1 | 1 | 1 | 2 | n/a | n/a | n/a | 1 | 1 | 2 | 1 | 2 | | 2 | | 1 | | 2 | | 70.8 | | Good | |
| Shin et al. ^68^ | 2 | 2 | 1 | 2 | n/a | n/a | n/a | 1 | 1 | 2 | 2 | 2 | | 2 | | 2 | | 2 | | 87.5 | | Strong | |
| Shuai et al. ^69^ | 2 | 1 | 2 | 1 | n/a | n/a | n/a | 1 | 1 | 2 | 2 | 1 | | 2 | | 2 | | 2 | | 79.2 | | Good | |
| Takeuchi et al. ^70^ | 2 | 1 | 2 | 2 | n/a | n/a | n/a | 1 | 1 | 2 | 2 | 2 | | 2 | | 2 | | 2 | | 87.5 | | Strong | |
| Takeuchi et al. ^71^ | 2 | 1 | 1 | 2 | n/a | n/a | n/a | 1 | 1 | 2 | 2 | 2 | | 2 | | 2 | | 2 | | 83.3 | | Strong | |
| Tan et al. ^72^ | 1 | 1 | 2 | 1 | n/a | n/a | n/a | 1 | 1 | 2 | 2 | 1 | | 1 | | 1 | | 2 | | 66.7 | | Good | |
| Tarawit et al. ^73^ | 2 | 2 | 1 | 2 | n/a | n/a | n/a | 1 | 1 | 1 | 1 | 2 | | 2 | | 1 | | 2 | | 75 | | Good | |
| Wanders et al.^74^ | 2 | 2 | 2 | 2 | n/a | n/a | n/a | 1 | 1 | 2 | 2 | 2 | | 2 | | 2 | | 2 | | 91.7 | | Strong | |
| Wang et al. ^75^ | 2 | 2 | 1 | 2 | n/a | n/a | n/a | 1 | 1 | 2 | 2 | 2 | | 2 | | 2 | | 0 | | 79.2 | | Good | |
| Wei et al. ^76^ | 2 | 2 | 1 | 1 | n/a | n/a | n/a | 1 | 1 | 2 | 2 | 2 | | 1 | | 1 | | 2 | | 75 | | Good | |
| Wingood et al. ^77^ | 2 | 2 | 2 | 2 | n/a | n/a | n/a | 1 | 1 | 2 | 2 | 2 | | 2 | | 2 | | 2 | | 91.7 | | Strong | |
| Woods et al. ^78^ | 2 | 1 | 1 | 2 | n/a | n/a | n/a | 1 | 1 | 1 | 1 | 2 | | 1 | | 1 | | 2 | | 66.7 | | Good | |
| Wu et al. ^79^ | 2 | 2 | 2 | 2 | n/a | n/a | n/a | 1 | 1 | 1 | 1 | 1 | | 1 | | 1 | | 2 | | 70.8 | | Good | |
| Wu et al. ^80^ | 2 | 2 | 2 | 2 | n/a | n/a | n/a | 1 | 1 | 2 | 2 | 2 | | 2 | | 2 | | 2 | | 91.7 | | Strong | |
| Wu et al. ^81^ | 1 | 1 | 1 | 2 | n/a | n/a | n/a | 1 | 1 | 2 | 1 | 1 | | 1 | | 1 | | 2 | | 62.5 | | Good | |
| Xiong et al. ^82^ | 1 | 2 | 2 | 2 | n/a | n/a | n/a | 1 | 1 | 2 | 2 | 1 | | 1 | | 1 | | 2 | | 75 | | Good | |
| Xu et al.^83^ | 2 | 2 | 2 | 2 | n/a | n/a | n/a | 1 | 1 | 2 | 2 | 2 | | 2 | | 1 | | 2 | | 87.5 | | Strong | |
| Yang et al. ^84^ | 2 | 1 | 2 | 2 | n/a | n/a | n/a | 1 | 1 | 2 | 2 | 2 | | 2 | | 2 | | 2 | | 87.5 | | Strong | |
| Yuan et al. ^85^ | 2 | 2 | 1 | 2 | n/a | n/a | n/a | 1 | 1 | 2 | 2 | 2 | | 2 | | 2 | | 2 | | 87.5 | | Strong | |
| Yuan et al. ^86^ | 1 | 1 | 2 | 2 | n/a | n/a | n/a | 1 | 1 | 2 | 1 | 1 | | 2 | | 2 | | 2 | | 75 | | Good | |
| Yu et al. ^87^ | 1 | 2 | 2 | 2 | n/a | n/a | n/a | 1 | 1 | 2 | 2 | 1 | | 2 | | 1 | | 2 | | 79.2 | | Strong | |
| Zhao et al. ^88^ | 1 | 2 | 1 | 2 | n/a | n/a | n/a | 1 | 1 | 2 | 1 | 2 | | 2 | | 2 | | 2 | | 79.2 | | Strong | |
| Zhang et al. ^89^ | 1 | 1 | 1 | 1 | n/a | n/a | n/a | 1 | 1 | 1 | 1 | 1 | | 0 | | 1 | | 1 | | 45.8 | | Poor | |
| Zhu et al. ^90^ | 2 | 2 | 2 | 2 | n/a | n/a | n/a | 1 | 1 | 2 | 2 | 1 | | 2 | | 1 | | 2 | | 83.3 | | Strong | |

**List of acronyms and abbreviations**

ADL: Activities of Daily Living; AD: Alzheimer’s disease; APOE: Apolipoprotein E; BMI: body mass index; B: Beta-coefficient; BDI: Beck Depression Inventory; CERAD: Consortium to Establish a Registry for Alzheimer’s Disease; CSI-D: Community Screening Instrument for Dementia; CI: Confidence Interval; CDT: Centre for Doctoral Training; CES-D: Centre for Epidemiologic Studies Depression Scale; CS: Cross-Sectional; FDR: False Discovery Rate; FDR: False Discovery Rate; F: F-distribution; HR: Hazard Ratio; HIV: Human Immunodeficiency; ICD-9: International Classification of disease 10^th^ Revision; IADL: Instrumental Activities of Daily Living; LO: Longitudinal Observational; MMSE: Mini-Mental State Examination; MCI: Mild Cognitive Impairment; MoCA: Montreal Cognitive Assessment; M: Mean; OR: Odds Ratio; PA: Physical Activity; r: correlation coefficient; RR: Relative ratio; SD: Standard Deviation; SES: Socioeconomic Status; SE: Standard Error; UKB: UK Biobank; TMI: Training in Mental Imagery; WM: Working Memory; WMC: Working Memory Capacity; z: Standard score.

**References**

1. Harvey PD. Domains of cognition and their assessment. *Dialogues Clin Neurosci* 2019; 21: 227-237.

2. Diamond A. Executive functions. *Annu Rev Psychol* 2013; 64: 135-168.

3. Riello M, Rusconi E and Treccani B. The role of brief global cognitive tests and neuropsychological expertise in the detection and differential diagnosis of dementia. *Front Aging Neurosci* 2021; 13: 648310-648310.

4. Henderson AS and Jorm AF. *Dementia*. Chichester, UK: John Wiley & Sons, Ltd, 2000, pp.1-68.

5. Douglas KM, Gallagher P, Robinson LJ, et al. Prevalence of cognitive impairment in major depression and bipolar disorder. *Bipolar Disord* 2018; 20: 260-274.

6. Allen MS, Laborde S and Walter EE. Health-related behavior mediates the association between personality and memory performance in older adults. *J Appl Gerontol* 2019; 38: 232-252.

7. Anaraky RG, Schuster AM and Cotten SR. Can changes in older adults' technology use patterns be used to detect cognitive decline? *Gerontologist* 2024; 64: gnad158.

8. Anatürk M, Suri S, Smith SM, et al. Leisure activities and their relationship with MRI measures of brain structure, functional connectivity, and cognition in the UK Biobank cohort. *Front Aging Neurosci* 2021; 13: 734866.

9. Bakrania K, Edwardson CL, Khunti K, et al. Associations between sedentary behaviors and cognitive function: cross-sectional and prospective findings from the UK Biobank. *Am J Epidemiol* 2018; 187: 441-454.

10. Bernstein JPK, Dorociak K, Mattek N, et al. Unobtrusive, in-home assessment of older adults' everyday activities and health events: associations with cognitive performance over a brief observation period. *Neuropsychol Dev Cogn B Aging Neuropsychol Cogn* 2022; 29: 781-798.

11. Bernstein JPK, Dorociak KE, Mattek N, et al. Passively-measured routine home computer activity and application use can detect mild cognitive impairment and correlate with important cognitive functions in older adulthood. *J Alzheimers Dis* 2021; 81: 1053-1064.

12. Brooker H, Wesnes KA, Ballard C, et al. An online investigation of the relationship between the frequency of word puzzle use and cognitive function in a large sample of older adults. *Int J Geriatr Psychiatry* 2019; 34: 921-931.

13. Brooker H, Wesnes KA, Ballard C, et al. The relationship between the frequency of number-puzzle use and baseline cognitive function in a large online sample of adults aged 50 and over. *Int J Geriatr Psychiatry* 2019; 34: 932-940.

14. Cansino S, Torres-Trejo F, Estrada-Manilla C, et al. Effects of different types of leisure activities on working memory across the adult lifespan. *Psychol Res* 2024; 88: 1981-1995.

15. Cegolon A and Jenkins A. Older adults, cognitively stimulating activities and change in cognitive function. *Int J Lifelong Educ* 2022; 41: 405-419.

16. Chen F and Yoshida H. Lifestyle habits and the risk factors of dementia: Evidence from Japan. *Geriatr Gerontol Int* 2021; 21: 203-208.

17. Choi EY, Wisniewski KM and Zelinski EM. Information and communication technology use in older adults: a unidirectional or bi-directional association with cognitive function? *Comput Human Behav* 2021; 121: 106813.

18. Coelho L, Hauck K, McKenzie K, et al. The association between sedentary behavior and cognitive ability in older adults. *Aging Clin Exp Res* 2020; 32: 2339-2347.

19. Covey TJ, Shucard JL, Bukoskey E, et al. History of playing video games is associated with better neuropsychological performance in people with multiple sclerosis. *J Cogn Enhanc* 2024; 8: 107-117.

20. Cutting J, Copeland B and McNab F. Higher working memory capacity and distraction-resistance associated with strategy (not action) game playing in younger adults, but puzzle game playing in older adults. *Heliyon* 2023; 9: e19098.

21. Da Ronch C, Canuto A, Volkert J, et al. Association of television viewing with mental health and mild cognitive impairment in the elderly in three European countries, data from the MentDis_ICF65+ project. *Ment Health Phys Act* 2015; 8: 8-14.

22. Fajersztajn L, Di Rienzo V, Nakamura CA, et al. Watching tv and cognition: The SPAH 2-year cohort study of older adults living in low-income communities. *Front Neurol* 2021; 12: 628489.

23. Fancourt D and Steptoe A. Television viewing and cognitive decline in older age: findings from the english longitudinal study of ageing. *Sci Rep* 2019; 9: 2851.

24. Feng TD, Feng ZY, Jiang LL, et al. Associations of health behaviors, food preferences, and obesity patterns with the incidence of mild cognitive impairment in the middle-aged and elderly population: an 18-year cohort study. *J Affect Disord* 2020; 275: 180-186.

25. Floud S, Balkwill A, Sweetland S, et al. Cognitive and social activities and long-term dementia risk: the prospective UK million women study. *Lancet Public Health* 2021; 6: e116-e123.

26. Green CP, Mao LK and O'Sullivan V. Internet usage and the cognitive function of retirees. *J Econ Behav Organ* 2021; 190: 747-767.

27. Hamer M and Stamatakis E. Prospective study of sedentary behavior, risk of depression, and cognitive impairment. *Med Sci Sports Exerc* 2014; 46: 718-723.

28. Hartanto A, Yong JC, Toh WX, et al. Cognitive, social, emotional, and subjective health benefits of computer use in adults: a 9-year longitudinal study from the midlife in the united states (MIDUS). *Comput Human Behav* 2020; 104: 1-11.

29. Heisz JJ, Vandermorris S, Wu J, et al. Age differences in the association of physical activity, sociocognitive engagement, and tv viewing on face memory. *Health Psychol* 2015; 34: 83-88.

30. Hoang TD, Reis J, Zhu N, et al. Effect of early adult patterns of physical activity and television viewing on midlife cognitive function. *JAMA Psychiatry* 2016; 73: 73-79.

31. Hou HY, Jia XZ, Wang P, et al. Intrinsic resting-state activity in older adults with video game experience. *Front Aging Neurosci* 2019; 11: 119.

32. Ihle A, Bavelier D, Maurer J, et al. Internet use in old age predicts smaller cognitive decline only in men. *Sci Rep* 2020; 10: 8969.

33. Ivleva V, Kairys A and Jurkuvenas V. Internet use, leisure activities, and memory performance among 65+ residents of Baltic states. *Socialine Teorija Empirija Politika Praktika* 2023; 27: 84-99.

34. Janoutová J, Kovalová M, Machaczka O, et al. Risk factors for Alzheimer’s disease: an epidemiological study. *Curr Alzheimer Res* 2021; 18: 372-379.

35. Jia Y, Shi M, Yang P, et al. Associations of computer gaming with incident dementia, cognitive functions, and brain structure: a prospective cohort study and Mendelian randomization analysis. *Alzheimers Res Ther* 2024; 16: 131.

36. Jopp D and Hertzog C. Activities, self-referent memory beliefs, and cognitive performance: evidence for direct and mediated relations. *Psychol Aging* 2007; 22: 811-825.

37. Jung MS and Chung E. Television viewing and cognitive dysfunction of Korean older adults. *Healthcare* 2020; 8: 547.

38. Karsazi H, Rezapour T, Ghamsari ASM, et al. Which intellectual activities are related to cognitive reserve? Introduction and testing a three-dimensional model. *Psychol Res* 2024; 88: 1081-1091.

39. Kesse-Guyot E, Charreire H, Andreeva VA, et al. Cross-sectional and longitudinal associations of different sedentary behaviors with cognitive performance in older adults. *PLoS One* 2012; 7: e47831-e47831.

40. Kim S, Koo M and Nam K. Game experience leads to improvement in cognitive functioning of the early middle-aged adults in contrast with the young-aged adults. *Comput Human Behav* 2022; 129: 107153.

41. Krell-Roesch J, Syrjanen JA, Vassilaki M, et al. Quantity and quality of mental activities and the risk of incident mild cognitive impairment. *Neurology* 2019; 93: e548-e558.

42. Krell-Roesch J, Vemuri P, Pink A, et al. Association between mentally stimulating activities in late life and the outcome of incident mild cognitive impairment, with an analysis of the APOE ϵ4 genotype. *JAMA Neurol* 2017; 74: 332-338.

43. Kurita S, Doi T, Tsutsumimoto K, et al. Cognitive activity in a sitting position is protectively associated with cognitive impairment among older adults. *Geriatr Gerontol Int* 2019; 19: 98-102.

44. Kurita S, Doi T, Tsutsumimoto K, et al. Computer use and cognitive decline among Japanese older adults: a prospective cohort study. *Arch Gerontol Geriatr* 2021; 97: 104488.

45. Lindstrom HA, Fritsch T, Petot G, et al. The relationships between television viewing in midlife and the development of Alzheimer’s disease in a case-control study. *Brain Cogn* 2005; 58: 157-165.

46. Lin YK, Peters K and Chen IH. Television watching, reading, cognition, depression and life satisfaction among middle-aged and older populations: a group-based trajectory modelling analysis of national data. *Health Soc Care Community* 2022; 30: E5661-E5672.

47. Maasakkers CM, Claassen JAHR, Gardiner PA, et al. The association of sedentary behaviour and cognitive function in people without dementia: a coordinated analysis across five cohort studies from COSMIC. *Sports Med* 2020; 50: 403-413.

48. Maasakkers CM, Claassen JAHR, Scarlett S, et al. Is there a bidirectional association between sedentary behaviour and cognitive decline in older adults? Findings from the Irish Longitudinal Study on Ageing. *Prev Med Rep* 2021; 23: 101423.

49. Mai S, Cai J, Zheng W, et al. Path analysis of social engagement, loneliness and cognitive function among Chinese older adults. *Psychol Health Med* 2023; 28: 1087-1099.

50. Major L, Simonsick EM, Napolitano MA, et al. Domains of sedentary behavior and cognitive function: The health, aging, and body composition study, 1999/2000 to 2006/2007. *J Gerontol A Biol Sci Med Sci* 2023; 78: 2035-2041.

51. Mao C, Li ZH, Lv YB, et al. Specific leisure activities and cognitive functions among the oldest-old: The chinese longitudinal healthy longevity survey. *J Gerontol A Biol Sci Med Sci* 2020; 75: 739-746.

52. Mariano J, Marques S, Ramos MR, et al. Cognitive functioning mediates the relationship between self-perceptions of aging and computer use behavior in late adulthood: Evidence from two longitudinal studies. *Comput Human Behav* 2021; 121: 106807.

53. Mariano J, Marques S, Ramos MR, et al. Internet use by middle-aged and older adults: Longitudinal relationships with functional ability, social support, and self-perceptions of aging. *Psychol Aging* 2021; 36: 983-995.

54. Mellow ML, Crozier AJ, Dumuid D, et al. How are combinations of physical activity, sedentary behaviour and sleep related to cognitive function in older adults? A systematic review. *Exp Gerontol* 2022; 159: 111698.

55. Miller LR, Reed C, Divers R, et al. Baseline differences in driving frequency as a predictor of cognitive decline and Alzheimer’s disease. *J Geriatr Psychiatry Neurol* 2024; 37: 14-23.

56. Nemoto Y, Sato S, Kitabatake Y, et al. Do the impacts of mentally active and passive sedentary behavior on dementia incidence differ by physical activity level? A 5-year longitudinal study. *J Epidemiol* 2023; 33: 410-418.

57. Nemoto Y, Sato S, Takahashi M, et al. The association of single and combined factors of sedentary behavior and physical activity with subjective cognitive complaints among community-dwelling older adults: Cross-sectional study. *PLoS One* 2018; 13: e0195384.

58. Olanrewaju O, Koyanagi A, Tully M, et al. Sedentary behaviours and cognitive function among community dwelling adults aged 50+years: Results from the Irish longitudinal study of ageing. *Ment Health Phys Act* 2020; 19: 100344.

59. Raichlen DA, Klimentidis YC, Sayre MK, et al. Leisure-time sedentary behaviors are differentially associated with all-cause dementia regardless of engagement in physical activity. *Proc Natl Acad Sci U S A* 2022; 119: e2206931119.

60. Ramos H, Alacreu M, Guerrero MD, et al. Lifestyle variables such as daily internet use, as promising protective factors against cognitive impairment in patients with subjective memory complaints. Preliminary results. *J Pers Med* 2021; 11: 1366.

61. Rawtaer I, Mahendran R, Kua EH, et al. Early detection of mild cognitive impairment with in-home sensors to monitor behavior patterns in community-dwelling senior citizens in Singapore: Cross-sectional feasibility study. *J Med Internet Res* 2020; 22: e16854.

62. Ringin E, Dunstan DW, McIntyre RS, et al. Differential associations of mentally-active and passive sedentary behaviours and physical activity with putative cognitive decline in healthy individuals and those with bipolar disorder: Findings from the UK Biobank cohort. *Ment Health Phys Act* 2023; 24: 100514.

63. Rosenberg DE, Bellettiere J, Gardiner PA, et al. Independent associations between sedentary behaviors and mental, cognitive, physical, and functional health among older adults in retirement communities. *J Gerontol A Biol Sci Med Sci* 2016; 71: 78-83.

64. Schaham NG, Buckman Z and Rand D. The effect of daily practice of puzzle-game apps on cognition in two groups of older adults: A pre-post experimental study. *Int J Environ Res Public Health* 2022; 19: 15454.

65. Sha F, Zhao ZY, Wei C, et al. Modifiable factors associated with reversion from mild cognitive impairment to cognitively normal status: A prospective cohort study. *J Alzheimers* 2022; 86: 1897-1906.

66. Shi JG, Liu ML, Fu GQ, et al. Internet use among older adults: Determinants of usage and impacts on individuals' well-being. *Comput Human Behav* 2023; 139: 107538.

67. Shimada H, Makizako H, Lee S, et al. Lifestyle activities and the risk of dementia in older Japanese adults. *Geriatr Gerontol Int* 2018; 18: 1491-1496.

68. Shin SH, Park S, Wright C, et al. The role of polygenic score and cognitive activity in cognitive functioning among older adults. *Gerontologist* 2021; 61: 319-329.

69. Shuai Z, Jingya Z, Qing W, et al. Associations between sedentary duration and cognitive function in older adults: A longitudinal study with 2-Year follow-up. *J Nutr Health Aging* 2023; 27: 656-662.

70. Takeuchi H and Kawashima R. A prospective study on the relationship between driving and non-occupational computer use with risk of dementia. *Front Aging Neurosci* 2022; 14: 854177.

71. Takeuchi H and Kawashima R. Effects of television viewing on brain structures and risk of dementia in the elderly: Longitudinal analyses. *Front Neurosci* 2023; 17: 984919.

72. Tan WY, Hargreaves CA, Kandiah N, et al. Association of multi-domain factors with cognition in the UK biobank study. *J Prev Alzheimers Dis* 2024; 11: 13-21.

73. Tarawit T, Tatree B and Thanakamon L. The study of prevalence and associated factors of dementia in the elderly. *Siriraj Med J* 2021; 73: 224–235.

74. Wanders L, Bakker EA, van Hout HPJ, et al. Association between sedentary time and cognitive function: A focus on different domains of sedentary behavior. *J Prev Med* 2021; 153: 106731.

75. Wang JYJ, Zhou DHD, Li J, et al. Leisure activity and risk of cognitive impairment: The Chongqing aging study. *Neurology* 2006; 66: 911-913.

76. Wei Z, Qiushi F, Fong JH, et al. Leisure participation and cognitive impairment among healthy older adults in China. *Res Aging* 2023; 45: 185-197.

77. Wingood M, Gell NM, Rosenberg DE, et al. Associations of cognitively active versus passive sedentary behaviors and cognition in older adults. *J Phys Act Health* 2024; 21: 928-938.

78. Woods SP, Thompson JL and Benge JF. Computer use: a protective factor for cognition in aging and HIV disease? *Aging Clin Exp Res* 2023; 35: 1711-1720.

79. Wu H, Gu Y, Du W, et al. Different types of screen time, physical activity, and incident dementia, Parkinson’s disease, depression and multimorbidity status. *Int J Behav Nutr Phys Act* 2023; 20: 130.

80. Wu YH, Lewis M and Rigaud AS. Cognitive function and digital device use in older adults attending a memory clinic. *Gerontol Geriatr Med* 2019; 5: 2333721419844886.

81. Wu Z, Pandigama DH, Wrigglesworth J, et al. Lifestyle enrichment in later life and its association with dementia risk. *JAMA Network Open* 2023; 6: e2323690.

82. Xiong SZ, Hou NX, Tang FF, et al. Association of cardiometabolic multimorbidity and adherence to a healthy lifestyle with incident dementia: a large prospective cohort study. *Diabetol Metab Syndr* 2023; 15: 208.

83. Xu CJ, Cao Z, Lu ZL, et al. Associations between recreational screen time and brain health in middle-aged and older adults: a large prospective cohort study. *J Am Med Dir Assoc* 2024; 25: 104990.

84. Yang SS, Ye ZL, Liu MY, et al. Variety and duration of different sedentary behaviors, inflammation, genetic susceptibility, and new-onset dementia in the older population. *J Am Med Dir Assoc* 2023; 24: 1396-1404.

85. Yuan M, Chen J, Han Y, et al. Associations between modifiable lifestyle factors and multidimensional cognitive health among community-dwelling old adults: Stratified by educational level. *Int Psychogeriatr* 2018; 30: 1465-1476.

86. Yuan S, Li W, Ling Y, et al. Associations of screen-based sedentary activities with all cause dementia, Alzheimer's disease, vascular dementia: a longitudinal study based on 462,524 participants from the UK Biobank. *BMC Public Health* 2023; 23: 2141.

87. Yu DD and Fiebig DG. Internet use and cognition among middle-aged and older adults in China: A cross-lagged panel analysis. *J Econ Ageing* 2020; 17: 100262.

88. Zhao X, Yuan L, Feng L, et al. Association of dietary intake and lifestyle pattern with mild cognitive impairment in the elderly. *J Nutr Health Aging* 2015; 19: 164-168.

89. Zhang J, Katsuta N, Takayama T, et al. Tangram puzzles in patients with neurocognitive disorders: A pilot study. *Psychiatry Int* 2023; 4: 404-415.

90. Zhu L, Wang YX, Wu YJ, et al. Longitudinal associations between the frequency of playing Mahjong and cognitive functioning among older people in China: evidence from CLHLS, 2008-2018. *Front Public Health* 2024; 12: 1352433.
